# Cell-type signatures of Alzheimer’s disease shared across population groups

**DOI:** 10.1038/s41586-026-10793-0

**Published:** 2026-07-15

**Authors:** Tain Luquez, Jonathan Algoo, Rebecca Chiu, Jason A. Mares, Archana Yadav, Matti Lam, Pallavi Gaur, Xiaoying Lai, Dylan I. Lee, Fahad Paryani, Rafe Batchelor, Irla Belli, Jordan Henry, Bat Hoter-Ishay, Courteney Mattison, Lindsey Starr, Tsering Lama, Berke Karaahmet, Wenqing Cao, Philip L. De Jager, Mariko Taga, Lisa L. Barnes, David X. Marquez, David A. Bennett, Ya Zhang, Vilas Menon

**Affiliations:** 1 Center for Translational and Computational Neuroimmunology, Department of Neurology, Columbia University Irving Medical Center, New York, NY USA; 2 Rush Alzheimer’s Disease Center, Rush University Medical Center, Chicago, IL USA; 3 Department of Kinesiology and Nutrition, University of Illinois Chicago, Chicago, IL USA

**Keywords:** Neurodegeneration, Molecular neuroscience, Neuroimmunology

## Abstract

Genomic studies at single-cell resolution have identified several cell types associated with clinical and pathological traits in Alzheimer’s disease^1–9^, but have not examined associations that are shared across populations. To bridge this gap, here we use single-nucleus RNA sequencing and assay for transposase-accessible chromatin with sequencing to profile cortical and subcortical regions in post-mortem brain-tissue samples from Latin, white (excluding Latin) and African American (excluding Latin) individuals. Using discrete and continuous dissections of molecular programs, we identify cell-type-specific clusters associated with Alzheimer’s disease in a region-specific manner across all three population groups, including microglial (*GPNMB*^+^ and *CD74*^+^ subgroups), astrocytic (*SERPINH1*^+^, *CD44*^+^ and *WIF1*^+^ subgroups) and neuronal (*SST*^+^ GABAergic and superficial-layer glutamatergic) signatures. We also report continuous gene-expression factors in astrocytes and oligodendrocytes that are not captured by discrete cluster assignments, but which show strong associations with disease phenotypes; these factors are enriched for genes associated with annotated functions such as lipid processing and neurotransmitter reuptake. Finally, we find that molecular programs reveal six distinct subgroups of individuals with cognitive impairment that span all three populations, are not captured by neuropathology, and are instead distinguished by molecular signatures that are not universally present but are nonetheless associated with ante-mortem impairment. Overall, our study identifies key cell types and gene programs implicated in Alzheimer’s disease that are shared across population groups, and underscores how representative sampling can capture both shared signatures and disease heterogeneity, thereby enabling better prioritization of key cell types for further investigation.

## Abstract (web summary)

Single-nucleus RNA-seq and ATAC-seq analyses on post-mortem brain samples from African American, Latin and white individuals identify cell-type-specific molecular signatures that are associated with cognitive impairment and/or Alzheimer’s disease across diverse population groups.

## Main

Alzheimer’s disease (AD), the most common cause of dementia in older individuals, has a complex aetiology that results in neurodegeneration and memory loss. Studies of post-mortem human tissue have identified amyloid-β aggregates and hyperphosphorylated tau tangles as the major pathologies associated with the disease, leading to a hypothesized cascade of pathology spreading, cellular alterations, circuit dysregulation and cognitive impairment^10^. Transcriptomics and proteomics studies at the bulk^11–17^ and single-cell^1–9^ levels have shed light on how almost all major cell classes in the brain seem to be altered in the disease. However, most of these studies have profiled human tissue from individuals of European descent, and given that the prevalence of AD differs across racial and ethnic groups^18^, it is unclear which previously reported cell-type-specific disease associations are generalizable to other populations. In addition, only a handful of studies have investigated multiple brain regions across large numbers of individuals and determined the extent to which AD associations differ across brain regions^6^.

In light of these gaps, we generated joint single-nucleus RNA sequencing (snRNA-seq) and assay for transposase-accessible chromatin with sequencing (snATAC-seq) data for three brain regions in 167 individuals who self-identified as African American (excluding Latin), white (excluding Latin) or Latin (of any race, as Latin is categorized as an ethnicity in the study). All of the individuals were participants in longitudinal studies, with prospective brain autopsy, based at the Rush University Alzheimer’s Disease Center: the Religious Orders Study and Memory and Aging Project (ROSMAP)^19^, the Minority Aging Research Study (MARS)^20^ and the Latino Core Study^21^. We used this dataset to address two key questions: (1) which cell-type-specific signatures are consistently associated with disease in all three population groups; and (2) which molecular subtypes of disease exist across this multi-ethnic set of individuals. We focused on associations with self-reported race and ethnicity, with a secondary analysis examining associations shared across groups defined by genetic ancestry. We identified a subset of cell-type-specific signals—mostly in microglia, astrocytes, neurons and oligodendrocytes—associated with AD phenotypes across all three population groups, with differential effects in each brain region. Some, but not all, of these signatures have been implicated in previous studies. Furthermore, our representative population sampling enabled the identification of six preliminary transcriptomically defined groups—three among individuals with mild cognitive impairment and three among individuals with dementia—building on previous evidence of molecular heterogeneity in older individuals with cognitive decline. Overall, this study highlights a set of candidate cell types and gene signatures that are altered in broad and specific groups of patients with AD.

## Cell-type and cluster overview

**Fig. 1:**
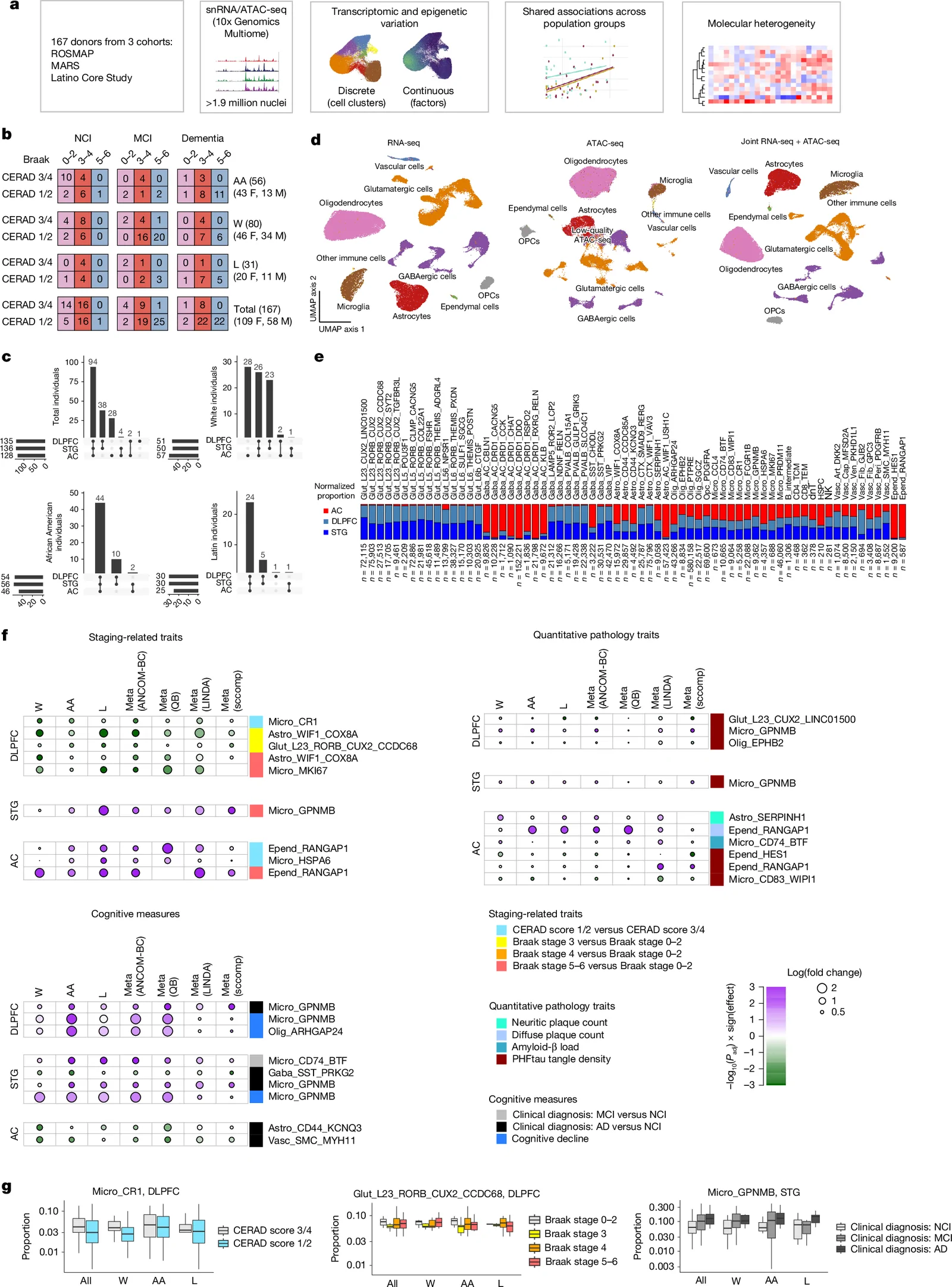
Shared associations of discrete clusters with clinical and pathological traits across population groups. **a**,Overview of the experimental and analytical workflow. **b**, Breakdown of pathological stages, cognitive diagnosis and self-reported racial and ethnic groups of individuals in this study. AA, African American (excluding Latin), L, Latin; W, white (excluding Latin). F, female; M, male. **c**, Upset plot showing the total number of individuals and number of individuals per population group with combinations of brain regions profiled. **d**, Uniform manifold approximation and projection (UMAP) visualization of nuclei (after quality control) based on snRNA-seq (left), snATAC-seq (middle) and joint snRNA-seq + snATAC-seq (right) data. OPCs, oligodendrocyte precursor cells. **e**, Relative abundance of each cell-type subcluster across each of the three regions (double-normalized to sum to 1 for each cluster). Astro, astrocytes; dnT, double-negative T cells; Epend, ependymal cells; Gaba, GABAergic neurons; Glut, glutamatergic neurons; HSPC, haematopoietic stem and progenitor cells; Micro, microglia; NK, natural killer cells; Olig, oligodendrocytes; TCM, T central memory; TEM, T effector memory; Vasc, vascular cells. **f**, *P* values and effect sizes for all subclusters with statistically significant (meta-analysis FDR-adjusted *P* < 0.1, consistent directionality and meta-analysis *I*^2^ < 50% in at least two out of four compositional analysis methods) associations with pathological and cognitive phenotypes across all three population groups. For visualization, effect sizes and FDR-adjusted *P* values for each population group are from the ANCOM-BC method. QB, quasibinomial logistic regression. ‘Cognitive decline’ refers to the steepness of the slope of ante-mortem cognitive decline (positive values indicate worse decline, as described in the Methods). **g**, Distributions of specific clusters showing associations with AD phenotypes. Left, the *CR1*^+^ myeloid cluster is consistently less abundant in the DLPFC in individuals with CERAD scores 1 and 2 than in those with scores 3 and 4. Middle, the *RORB*^+^*CUX2*^+^*CCDC68*^+^ glutamatergic neuronal cluster is less abundant in the DLPFC in individuals at Braak stage 3 than in those at Braak stages 0–2. Right, the *GPNMB*^+^ microglia cluster is more abundant in the STG in individuals with a clinical diagnosis of MCI or AD. Box and whisker plots show the median (centre line), the interquartile range (IQR; box limits), and whiskers extending to the most extreme values no further than 1.5 times the IQR from the top or bottom of the box limits.

**Extended Data Fig. 1:**
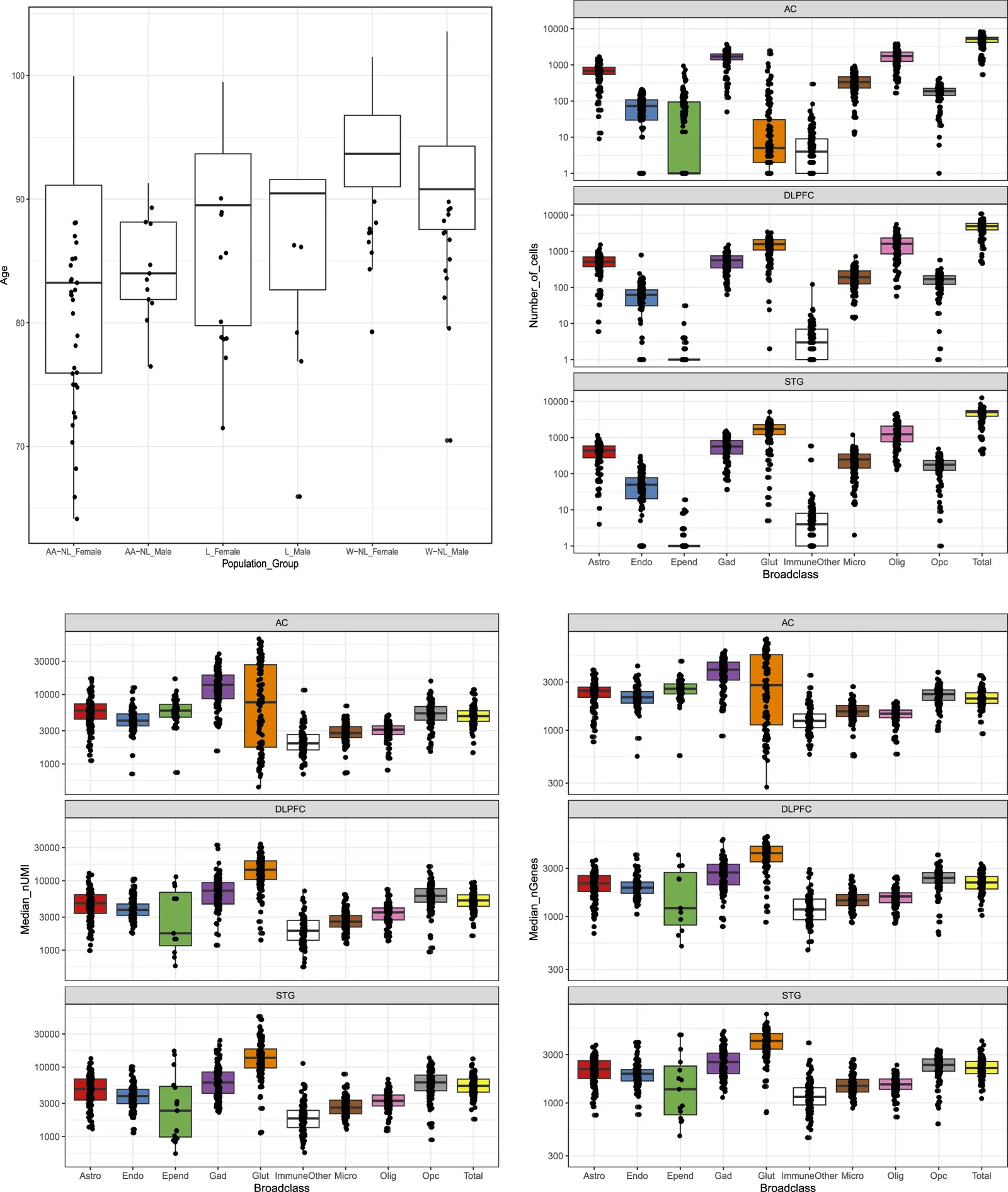
Demographics and gene and transcript detection. Distributions of age (top left) across donor groups, nuclei numbers per cell type per region (top right), total gene counts per nucleus per cell type per region (bottom left), and total transcript (Unique Molecular Identifier) counts per nucleus per cell type per region. For age, the box plot distribution includes all participants whose brains were profiled, but individual points for those older than 90 have been removed from the visualization.

**Extended Data Fig. 2:**
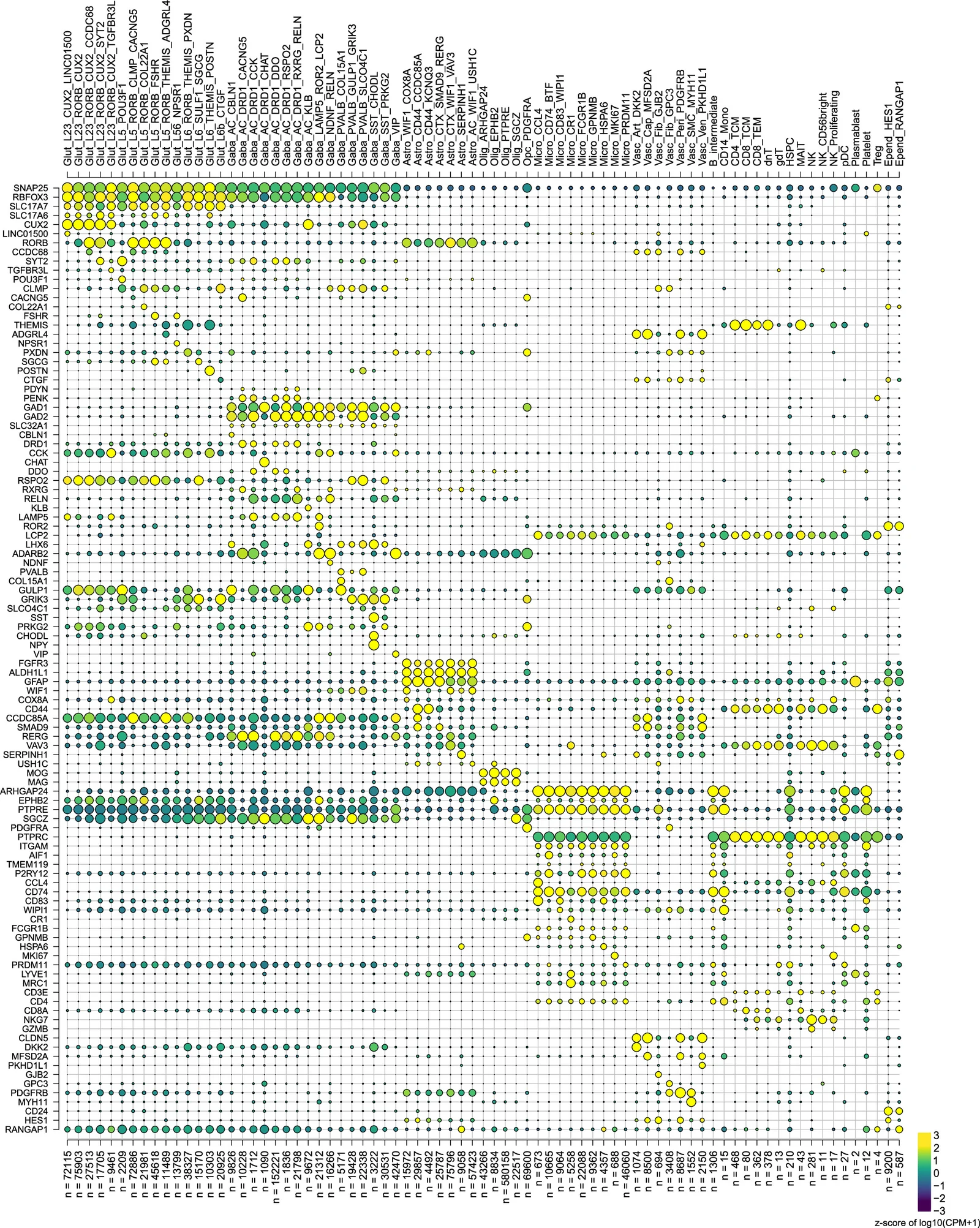
Cluster-specific combinatorial marker genes. Dot plot showing the fraction of nuclei and mean gene expression in each cluster of broad cell class markers as well as key distinguishing genes between subclusters within each broad class.

To identify differences in individuals with AD-related pathology and/or cognitive impairment relative to cognitively healthy individuals, we performed joint snRNA-seq and snATAC-seq on nuclei from 399 samples, derived from 56 self-identified African American participants, 31 self-identified Latin participants and 80 self-identified white participants (Fig. 1a,b). Three brain regions—the dorsolateral prefrontal cortex (DLPFC), superior temporal gyrus (STG) and anterior caudate (AC)—were profiled from 94 individuals, with the remaining individuals having one or two regions profiled (Fig. 1c) according to tissue availability. After sample demultiplexing, ambient RNA subtraction and quality control filtering, we iteratively clustered around 1.9 million nuclei using snRNA-seq data (Methods). The first clustering round separated the major cell-type classes, and three subsequent rounds of clustering identified groups of nuclei comprising doublets. The final round of clustering identified subpopulations within each major class (Fig. 1d,e and Extended Data Figs. 1 and 2), with the optimal number of subclusters determined by two clustering robustness metrics (Methods). The final set of nuclei had a median of 2,156 genes and 5,149 unique molecular identifiers (transcripts) detected (Extended Data Fig. 1). We did not find further subdivisions among these transcriptomic subclusters on the basis of snATAC-seq data. This de novo clustering approach allowed us to generate a unified set of clusters across all three of our brain regions.

As expected, neuronal clusters showed regional specificity, with glutamatergic neurons found mainly in the two cortical regions. GABAergic neuronal clusters showed minimal overlap between the cortex and the caudate, except for one subclass marked by the expression of somatostatin (*SST*) and chondrolectin (*CHODL*) (Fig. 1e). Among glia, four clusters showed strong regional specificity (Fig. 1e): ependymal cells (found mainly in the caudate) and astrocytes (with *SMAD9*^+^*RERG*^+^ and *WIF1*^+^*VAV3*^+^ astrocytes in the cortex and *WIF1*^+^*USH1C*^+^ astrocytes in the caudate). Overall, we identified 61 clusters across all major classes (Extended Data Fig. 2), not including non-microglial immune cells, whose numbers were too low for robust clustering and were thus subject to label transfer (Methods).

Our primary goal was to identify cell subgroups that were consistently differentially abundant in relation to AD phenotypes across all three population groups. We examined discrete and continuous measures of AD phenotypes, including CERAD (Consortium to Establish a Registry for Alzheimer’s Disease) neuritic plaque score, neuritic plaque count, diffuse plaque count, amyloid-β load, Braak stage and paired helical filamental tau (PHFtau) tangle density, as well as clinical diagnoses of no cognitive impairment (NCI), mild cognitive impairment (MCI) or Alzheimer’s dementia, and the slope of cognitive decline^19^ (Methods). Sex, post-mortem interval and age at death were included as covariates, and education as an additional covariate for cognitive measures. We used a stratified approach in which we assessed cluster proportion associations in each group and then performed a meta-analysis across the three groups, keeping only results that had a false discovery rate (FDR)-adjusted *P* < 0.1, a consistent sign of association in all three groups and *I*^2^ < 50% (Methods and Supplementary Table 1); these criteria had to be met by at least two distinct compositional methods out of the four that were implemented (ANCOM-BC, quasibinomial logistic regression, LINDA and sccomp; Methods). It is important to note that this stratified approach can identify shared signatures associated with AD phenotypes despite the three population groups not being matched on demographic variables—most notably, age at death (Extended Data Fig. 1).

The strongest consistent signal across population groups was the higher proportion of *GPNMB*^+^ microglia in both cortical regions in individuals with a clinical diagnosis of Alzheimer’s dementia and/or a steeper slope of cognitive decline (Fig. 1f and Supplementary Table 1). Higher proportions of this microglial subgroup were also associated with a higher PHFtau tangle density in both cortical regions, and with later Braak stages in the STG. This latter finding is consistent with previous single-nucleus studies of the DLPFC^8^. *GPNMB*^+^ microglia show high expression of genes associated with lipid processing, cell migration and vascular reorganization (Supplementary Table 2). In addition, individuals with a clinical diagnosis of MCI have greater proportions of *CD74*-high microglia (enriched for mitochondrial-process-associated genes) in the STG (Fig. 1f and Supplementary Table 2). Finally, a *CR1*^+^ group of myeloid cells, which also expresses *MRC1* and other macrophage markers (Extended Data Fig. 2), is less abundant in the DLPFC in individuals with more severe CERAD scores (Fig. 1f,g). These associations are robust across a range of thresholds for minimum unique molecular identifiers (UMIs) and confidence of demultiplexing parameters (Supplementary Fig. 1 and Supplementary Table 3). Overall, these findings highlight a range of myeloid signatures across different brain regions that are shared across population groups.

Astrocyte clusters exhibited region-specific shared signatures associated with AD phenotypes, particularly in the caudate (Fig. 1f). *SERPINH1*^+^ astrocyte proportions were higher in the AC in individuals with higher neuritic plaque counts; this cluster is enriched in genes associated with lipid processing (Supplementary Table 2). By contrast, *CD44*^+^*KCNQ3*^+^ astrocytes were less abundant in the AC in individuals with a clinical diagnosis of AD dementia. Whereas *CD44*^+^ cortical astrocytes exhibit fibrous morphology^22^, *CD44*^+^ astrocytes in the caudate are less well studied, and their gene-expression profile shows enrichment for processes associated with immune-response signalling (Supplementary Table 2). The lower abundance of this astrocyte cluster in individuals with AD dementia might point to a protective role in the AC. In the DLPFC, *WIF1*^+^*COX8A*^+^ astrocytes were less abundant in individuals at Braak stages 3 and 5/6 than they were in individuals at Braak stages 0–2 (Fig. 1f). Thus, we identify multiple region-specific astrocytic signatures associated with AD phenotypes consistently across all three population groups.

We also confirmed two previous findings of selective neuronal vulnerability in AD. Cortical *SST*^+^ GABAergic neurons and a subset of superficial-layer glutamatergic neurons have both been identified as less prevalent in individuals with dementia and AD pathology^5,6,8,9,23^; here we confirm that both of these phenomena are shared across population groups (Fig. 1f and Supplementary Table 1), underscoring the robust nature of this selective neuronal loss in AD. Other reported findings, such as the selective loss of *LAMP5*^+^*RELN*^+^ GABAergic neurons in the cortex, are not shared across population groups to the same extent (Supplementary Table 1), suggesting that some neuronal associations with AD show greater variability across population groups.

## Continuous variation and factor analysis

**Fig. 2:**
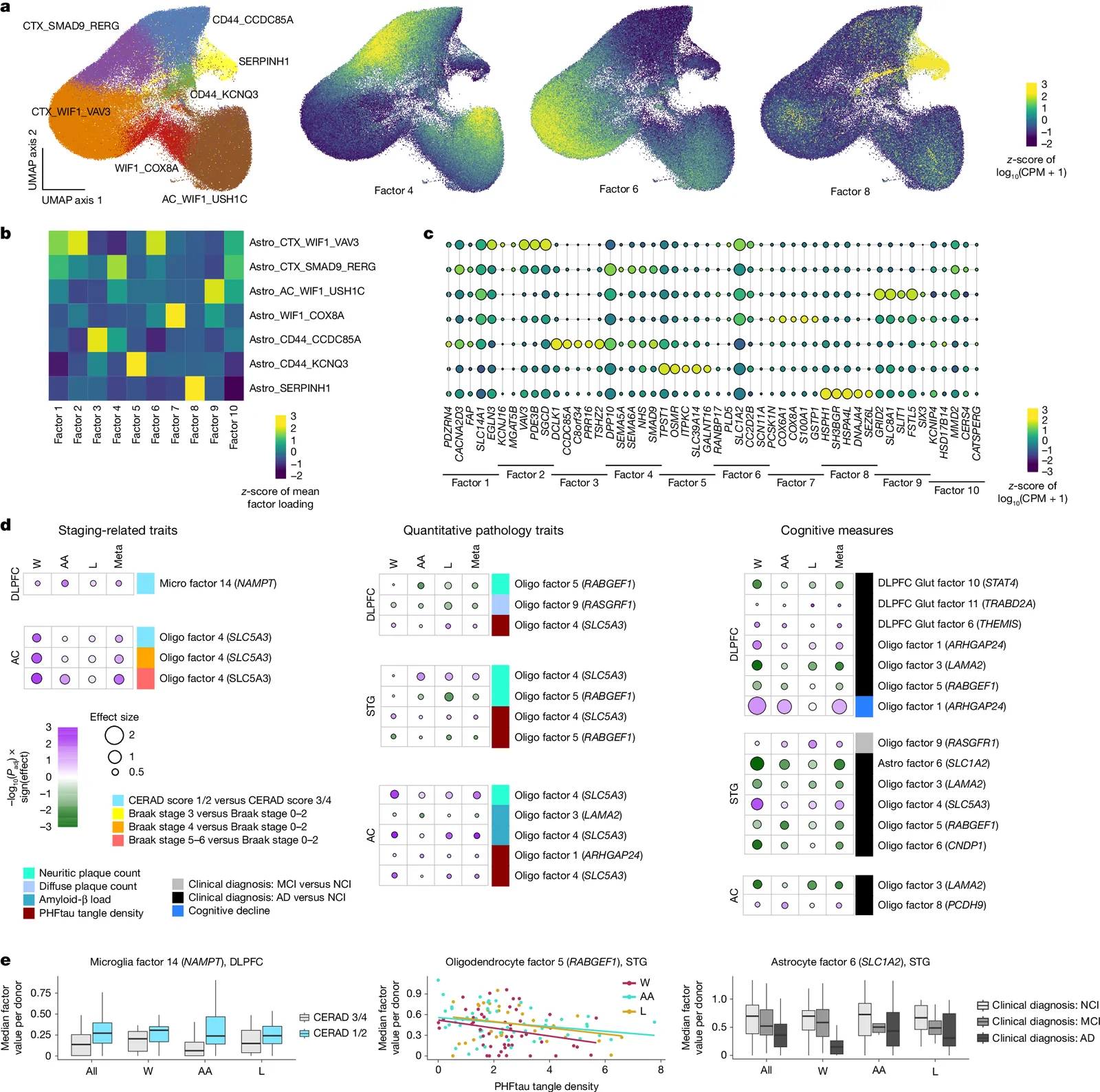
Continuous variation in cell-type signatures and shared associations with clinical and pathological traits across population groups. **a**, Visualization of scHPF-derived factor scores versus cluster identities in astrocytes. CPM, counts per million mapped reads. **b**, Heat map showing the median factor score in each cluster. Whereas some factor scores (for example, factor 8) are high in nuclei belonging to a specific cluster, others (for example, factors 1, 4 and 6) are represented in multiple clusters. **c**, Expression of genes with the top five weights in each factor, plotted by discrete cluster. Consistent with the representations in **a**,**b**, some factors are cluster-specific, whereas others span multiple clusters. **d**, Effect sizes and *P* values (from ANCOM-BC) of factor associations with consistent and significant (meta-analysis FDR-adjusted *P* < 0.05, consistent directionality of effect and meta-analysis *I*^2^ < 50%) signals in all population groups, in the same format as Fig. 1f. Unlike with the discrete clustering results in Fig. 1, oligodendrocytes are highly represented in the factor-based analysis, especially factor 4 (higher in individuals with disease) and factors 3, 5 and 6 (lower in individuals with disease). **e**, Examples of factors with associations with AD traits, in the same format as Fig. 1g. Box and whisker plots show the median (centre line), the IQR (box limits), and whiskers extending to the most extreme values no further than 1.5 times the IQR from the top or bottom of the box limits.

Previous work by us and others^5,24^ has suggested that assigning nuclei to discrete clusters might mean that continuous signatures associated with disease are missed. This is particularly the case for glial cells, in which distinct and dynamic signatures can exist in the same major class without being pre-specified by developmental lineage. Using the single-cell hierarchical Poisson factorization (scHPF) workflow^25^, we identified factors capturing continuous modes of correlated gene expression in each of our major cell classes. As an illustrative case, we identified ten factors in astrocytes (Fig. 2a). Some factors recapitulated the separation of nuclei into discrete clusters (for example, factor 8 was restricted to the *SERPINH1*^+^ cluster), whereas others captured coordinated gene expression spanning multiple clusters (Fig. 2b,c and Supplementary Fig. 2). Notably, some factors revealed signatures associated with AD phenotypes that are not well captured by our assignment of nuclei to discrete clusters. This was particularly the case for oligodendrocytes: oligodendrocyte factor 4 (with high expression of *SLC5A3*, and enriched in genes associated with immune signalling and lipid processing; Supplementary Table 2) was more prevalent in individuals with higher neuritic plaque counts, higher PHFtau tangle density and a clinical diagnosis of Alzheimer’s dementia, in both the STG and the AC (Fig. 2d and Supplementary Table 4). By contrast, oligodendrocyte factor 3 (high expression of *LAMA2*, and enriched for genes associated with synaptic-vesicle recycling) and factor 5 (high expression of *RABGEF1*, and enriched for cytoskeleton-associated genes) were expressed at lower levels in both cortical regions in individuals with a clinical diagnosis of Alzheimer’s dementia (Fig. 2d,e and Supplementary Table 4). In addition, astrocyte factor 6 (with high expression of *SLC1A2*) was expressed at lower levels in the STG in individuals with a clinical diagnosis of Alzheimer’s dementia (Fig. 2d,e). *SLC1A2* encodes the principal transporter that clears glutamate from the perisynaptic space, and its lower expression in AD suggests disruption at the synapse. As with the cluster-based analysis described above, these associations are robust to UMI and nucleus assignment thresholds (Supplementary Fig. 1 and Supplementary Table 4). Finally, we also ran weighted gene co-expression analysis (WGCNA)^26^ as an alternative approach to assess gene module associations (Supplementary Fig. 3 and Supplementary Table 5); oligodendrocyte module ME2 (containing many factor-3, factor-5 and factor-6 genes) showed lower expression in individuals with worse clinical phenotypes, whereas oligodendrocyte module ME4 (containing many factor-4 genes) showed the opposite trend. Astrocyte modules ME15 and ME18 (containing genes associated with the *SERPINH1*^+^ astrocytes and the *CD44*^+^*KCNQ3*^+^ astrocytes, respectively) both had higher expression in the AC in individuals with worse clinical outcomes. Collectively, these results highlight additional candidate gene-expression programs that might be depleted in individuals with cognitive impairments. This lends further credence to the suggestion that both discrete and continuous models of gene-expression variation should be considered when assessing disease associations—particularly in the case of glial cells, in which discrete taxonomies might not fully capture transcriptional variation.

## Regional specificity of associations

Certain cell-type associations are consistent across population groups in all three brain regions, whereas others seem to be region-specific. These region-specific findings could be genuine, or could be due to significance thresholds and differences in statistical power. By examining both the significance and the effect size for cell-type and factor associations across pairs of regions (Supplementary Fig. 4), we find that the associations of *GPNMB*^+^ microglia and oligodendrocyte factors with AD phenotypes have consistent effect sizes in all three brain regions, even if they do not reach statistical significance. By contrast, the associations of *CD74*-high microglia and *SST*^+^ GABAergic neurons with dementia have larger effect sizes in the STG, and the relative depletion of astrocyte factor 6 (*SLC1A2*-high) in dementia is restricted to the cortex (Supplementary Fig. 4). Although larger sample sizes will allow better distinction between cross-region effects and statistical power issues, this study already highlights multi-region and region-specific signatures shared across population groups.

## Associations based on genetic ancestry

**Extended Data Fig. 3:**
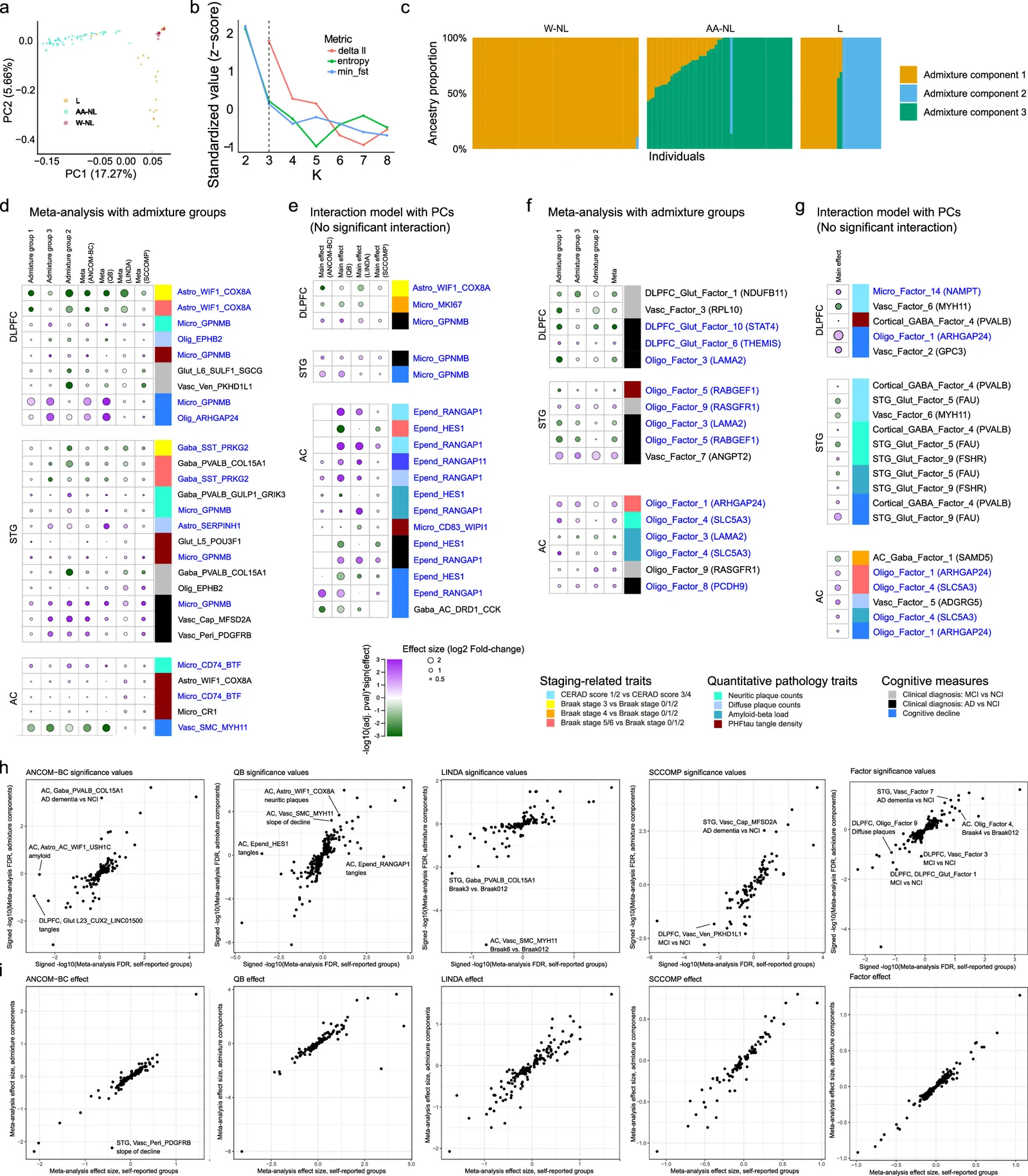
Shared signatures across ancestry groupings. Overview of genetic ancestry groupings and associations between clusters/factors and clinical and pathological traits across population groups defined by genetic ancestry. **a**, Distribution of individual donors along genetic ancestry PCs 1 and 2. Colours indicate self-reported race and ethnicity groups, showing an overall concordance with genetic ancestry variation. **b**, Assessment of optimal number of admixture components (k) based on multiple information theory metrics. k = 3 showed the biggest changes in log-likelihood, normalized ancestry entropy and minimum pairwise Fst. **c**, Stacked bar plots showing the relative contribution of admixture components (k = 3) in each donor, with donors grouped by self-reported race and ancestry. **d**, Summary of p-values and effect sizes for all cell clusters with statistically significant (meta-analysis FDR-adjusted p-value < 0.1, consistent directionality, and meta-analysis I^2^ < 50% in at least two out of four compositional analysis methods) and consistent associations with pathological and cognitive phenotypes across all three population groups defined by dominant admixture component. For visualization, effect sizes and FDR-adjusted p-values for each population group are from the ANCOM-BC method to assess cluster proportion associations. NCI: No cognitive impairment, MCI: Clinical diagnosis of Mild cognitive Impairment, AD: Clinical diagnosis of Alzheimer’s dementia. **e**, Summary of p-values and main effect sizes from models relating cluster proportions to phenotypes including interactions with genetic ancestry PCs 1 and 2. All visualizations are only for region–cluster–phenotype models in which the main effect (with phenotype of interest) was significant and neither the interaction with PC1 nor the interaction with PC2 was significant; this combination highlights cluster–phenotype associations that are not differential across genetic ancestry PCs 1 and 2. Cell clusters in blue were also found to have associations shared across groups based on self-reported race and ethnicity (Fig. 1). **f**,**g**, Same as **d**,**e**, but using the linear model for factor association with a meta-analysis on admixture-defined population groups (**f**) and interaction model with genetic ancestry PCs (**g**). **h**, Visualization comparing meta-analysis p-values for cluster/factor associations with phenotypes using self-reported race and ethnicity groupings (x-axis) versus genetic admixture-defined groupings (y-axis). From left to right, panels represent ANCOM-BC cluster associations, the quasibinomial regression model cluster associations, LINDA cluster associations, sccomp cluster associations, and linear model for factor associations. Labelled combinations of region, cell type, and phenotype represent associations with substantially different meta-analyses p-values between the x- and y-axes. **i**, Same as **h**, but showing effect sizes instead of p-values.

A key question is whether the molecular signatures that show shared association across self-identified population groups are also shared when considering groups defined by genetic ancestry. To address this question, we used existing whole-genome sequencing (WGS) data from the AD Knowledge Portal for 151 of the 167 individuals (see ‘Data availability’). We computed both genetic principal components (PCs) and admixture components (Extended Data Fig. 3a,b). We observed that PCs 1 and 2 explain a large portion of the variation (around 23%), and roughly align with self-reported race and ethnicity (Extended Data Fig. 3a). Similarly, an admixture analysis with three admixture components captured the primary ancestry structure, beyond which additional components reflected increasingly fine-scale subdivision (Extended Data Fig. 3b). Self-reported white individuals had mostly component 1; self-reported African American individuals had mostly component 3, with some individuals also showing varying levels of component 1; and self-reported Latin individuals had components 1 and 2 (Extended Data Fig. 3c).

We reran the associations of cell clusters and factors with both the WGS PCs and the admixture components, using an interaction model for the PCs (which have continuous values) and the same categorical approach for admixtures (based on the most dominant component) that was used for self-reported race and ethnicity in Figs. 1 and 2. We observed that most associations that were shared across self-reported race and ethnicity groups were also shared when donors were grouped by dominant admixture component or represented by their continuous genetic ancestry PCs (Extended Data Fig. 3d–g and Supplementary Tables 6–9). However, grouping individuals by genetic ancestry did highlight additional signatures that did not cross the FDR correction threshold in our self-reported race–ancestry analysis. Specifically, some vascular cell clusters and glutamatergic neuron factors show associations with cognitive phenotypes (Extended Data Fig. 3d–g). Overall, a comparison of the FDR-adjusted *P* values and effect sizes for associations using self-reported race and ethnicity, and genetic ancestry (Extended Data Fig. 3h,i), shows that these differences are likely to be due to statistical power rather than to environmental or genetic effects. However, we cannot definitely rule out the effects of environmental or unmeasured variables for some associations that differ between self-reported and genetic ancestry analyses.

## Potential driver genes from snATAC-seq

**Fig. 3:**
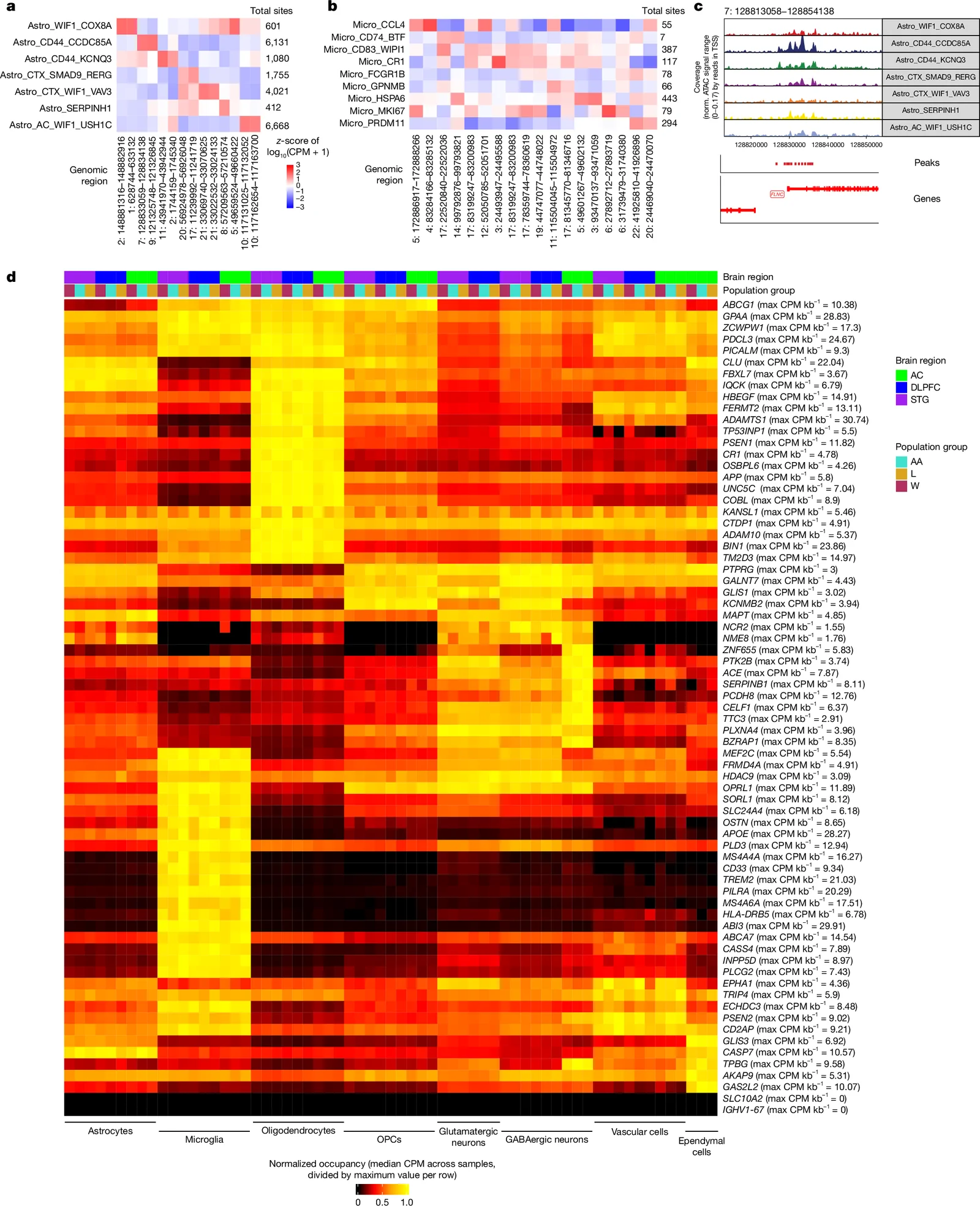
Differential chromatin occupancy across RNA-derived cell types and AD risk loci. **a**, Occupancy values (normalized counts per pseudobulked cluster) of the top two differential sites (columns) for each astrocyte cluster (rows). Numbers on the right of the heat map indicate the total number of differential sites (FDR-adjusted *P* < 0.05 using DESeq2 Wald test) per astrocyte cluster, in a one-versus-all comparison. Peak regions on the *x* axis are labelled as chromosome-start site–end site. **b**, Same as **a**, but for microglial clusters. **c**, Peak coverage tracks for one specific site shown to be differentially accessible across astrocyte clusters in **a**. TSS, transcription start site. **d**, Normalized chromatin occupancy values at each of around 70 AD-associated GWAS loci. Colours represent the total snATAC-seq counts over all nuclei for a given cell type, donor population group and brain region, which are then normalized to the maximum value for each locus across all combinations of donor, cell type and region. Several loci show visible differences in occupancy across cell types, whereas only GABAergic neurons exhibit notable region-specific differences, as expected given their distinct regional expression signatures. Only one locus (containing the *KANSL1* gene) shows consistent differences across the three population groups.

**Extended Data Fig. 4:**
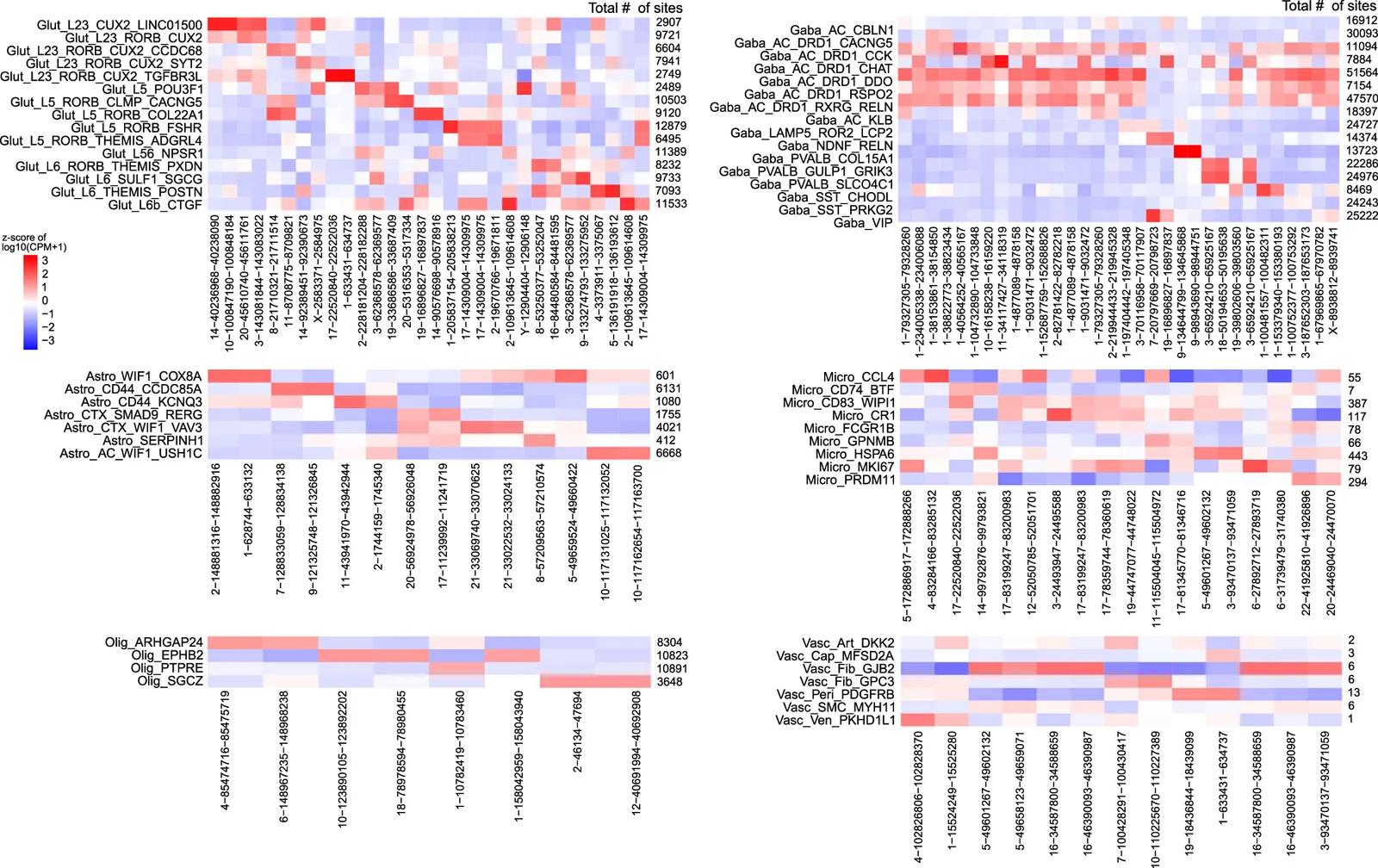
Cluster-enriched snATAC-seq occupancy. snATAC-seq-based occupancy for key cluster-enriched peaks, with clusters defined based on snRNA-seq; this is analogous to Fig. 3a,b. For each major cell class, the heat map represents the z-score of the mean peak CPM (pseudobulked over all nuclei of each cluster per sample). For each cluster, the top 2 differential peaks (versus all other clusters of that cell class) are included. Numbers on the right of each heat map indicate the total # of differential sites per cluster.

We next examined matched snATAC-seq data from the same nuclei to uncover putative drivers of cell signatures. Overall, our snATAC-seq data alone were not sufficient to identify robust subclusters in major cell types, and the data did not yield robust, statistically significant cell-type-specific snATAC-seq peaks that were directly associated with clinical phenotypes across all three population groups. This is consistent with previous studies, and the sparseness of the data could be a result of relatively shallow sequencing depth (around 30,000 reads per nucleus). By contrast, we identified tens to hundreds of peaks that differed significantly across our transcriptomically defined subclusters (Fig. 3a–c and Extended Data Fig. 4), consistent with previous studies^2^, and some chromatin regions near AD loci that are cell-type specific (Fig. 3d and Supplementary Table 10). Sequence motif enrichment analysis of these differential peaks yielded putative transcription-factor-binding sites enriched in each cluster. Cross-referencing these with transcription-factor expression in the relevant cell types then yielded a smaller set of transcription factors that might drive some aspect of cluster identity (Supplementary Table 11). Putative drivers included *HSF1* and *HSF2* in the *SERPINH1*^+^ astrocyte cluster, *EGR1* in the *GPNMB*^+^ microglia cluster and *PATZ1* and *ELF5* in the *WIF1*^+^*COX8A*^+^ astrocyte cluster. Similarly, putative drivers for factors included *WT1* in astrocyte factor 8, *RORA* and *ZNF449* in microglia factor 14 and several others in the oligodendrocyte factors. Notably, some putative transcription-factor drivers were shared across cell-type clusters and factors. For instance, *SREBF1* in the *RORB*^+^*CUX2*^+^*CCDC68*^+^ L23 glutamatergic cluster, *WIF1*^+^*USH1C*^+^ AC astrocyte cluster and oligodendrocyte factor 4 (Supplementary Table 11), underscoring potential co-regulation across several cell states.

## Consistency of open chromatin regions

In addition to identifying putative drivers of cell signatures associated with AD, we examined chromatin accessibility in each population at genetic loci that have been implicated in AD through genome-wide association studies (GWASs)^27–37^ (Fig. 3d and Supplementary Table 10). As expected, microglia showed the highest accessibility at previously described immune-gene loci (including loci containing *TREM2*, *CD33* and *HLA-DRB5*). Oligodendrocytes showed the highest accessibility at many loci, including those containing early-onset AD genes, such as *APP* and *PSEN1*, whereas neurons had the highest accessibility in loci containing *PLXNA4* and *NCR2*.

Despite the greater genetic diversity afforded by examining individuals from multiple backgrounds, we found only one AD-associated locus with potential differential occupancy across our population groups. This locus is on chromosome 17 (46,029,916–46,223,808 in GRCh38), proximal to the locus containing *MAPT*, and contains the *KANSL1* gene. It was identified in GWASs that studied mainly white individuals^27^ and African American individuals^38^. In our data, this locus is less accessible overall in African American individuals than it is in white individuals (Fig. 3d and Supplementary Table 12). This difference in accessibility is consistent across all major cell classes. However, it does not correspond to gene-expression differences in *KANSL1* itself, suggesting that the effect of regulatory elements in this locus acts elsewhere in a population-specific manner.

## Validation in intact tissue sections

**Fig. 4:**
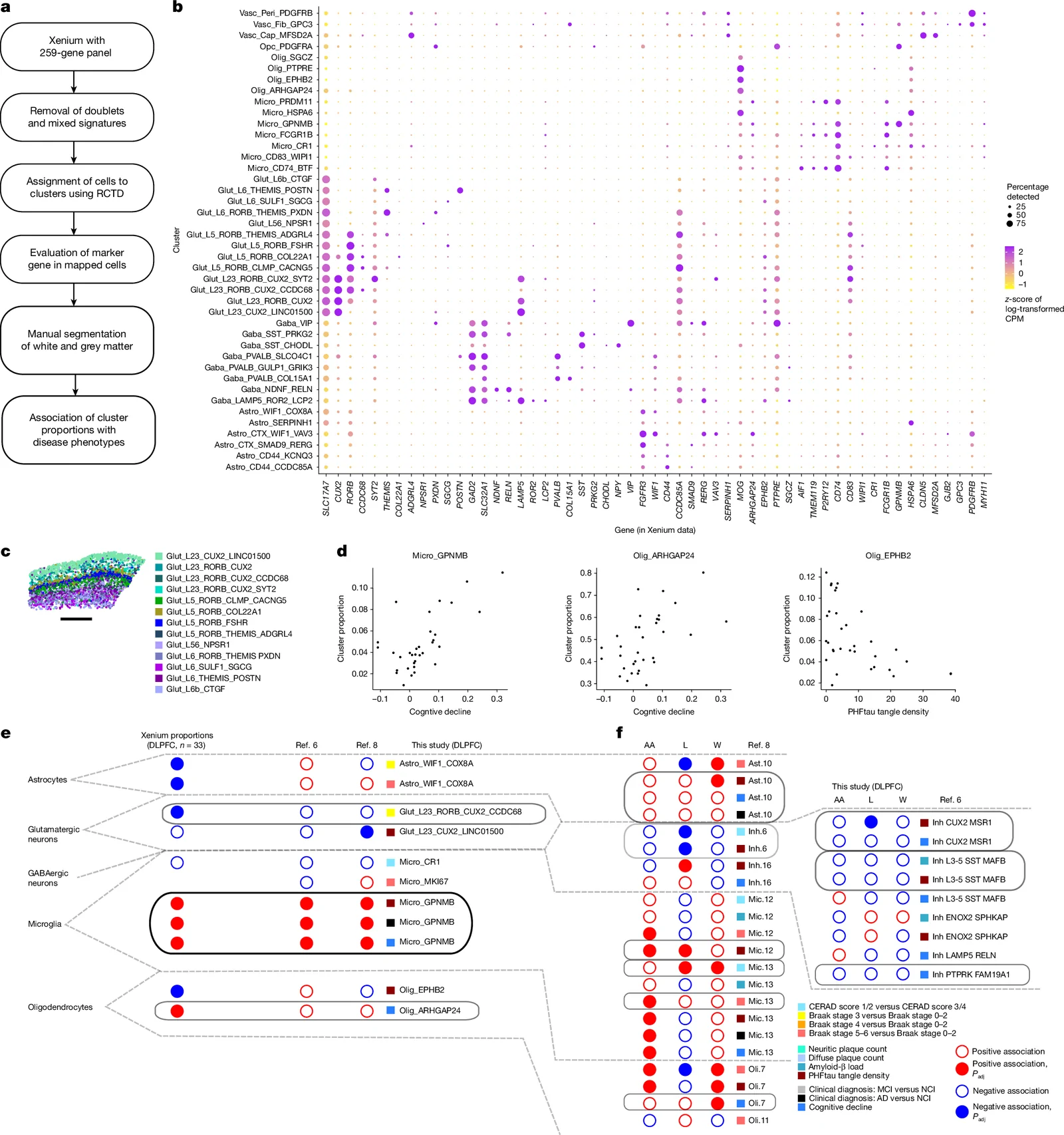
Validation of snRNA-seq findings through multiplexed ISH validation and comparison with other studies. **a**, Workflow for data generation and analysis using the Xenium platform. **b**, Dot plot showing the expression of snRNA-seq cluster markers in cells from 36 DLPFC sections (one per individual) profiled using the Xenium platform, after label transfer and cluster assignment using the RCTD pipeline. **c**, Tissue plot showing that the assignment of cells to glutamatergic clusters recapitulates the expected layer positions of clusters. Scale bar, 2 mm. **d**, Scatter plots showing the relative proportion of three cell clusters in intact tissue (Xenium) versus continuous measures of cognition (left and middle, with worse slope of cognitive decline on the right in each plot; see Methods for interpretation of this measure) and tau pathology (right); these associations were all found to be significant in the cell composition analysis from snRNA-seq data (Fig. 1f), and are recapitulated in intact tissue. **e**, Summary of snRNA-seq proportion associations from this study compared with Xenium data and two other studies^6,8^ of predominantly white individuals from ROSMAP. All association tests were run using ANCOM-BC. Colours indicate positive (red) or negative (blue) associations with clinical and pathological variables; solid circles represent FDR-adjusted *P* < 0.05; open circles indicate associations with *P*_adj_ > 0.05. Black boxes highlight strongly replicated findings (same directionality and statistical significance); grey boxes highlight findings with similar directionality and partial statistical significance. **f**, Similar to **e**, but using cluster labels from the two external studies in **e** transferred onto our study. Results are shown from mapping cluster labels from ref. ^8^ (left columns) or ref. ^6^ (right columns) onto this study. For the two external studies, only clusters reported as being statistically significantly associated with AD phenotypes are included.

To assess the robustness of signatures from our snRNA-seq analyses, we investigated the relative abundance of cell clusters in intact DLPFC tissue using the Xenium multiplexed in situ hybridization (ISH) platform. We designed a panel of 259 genes (Supplementary Table 13) covering major cell-type markers, cluster-specific genes and genes in known AD risk loci (Supplementary Table 10), and profiled tissue from 33 donors in our dataset (Fig. 4a). After mapping cluster identities to the cells in tissue sections (using the default cell segmentation pipeline from the Xenium Ranger software package, followed by the robust cell-type decomposition (RCTD) workflow^39^; Methods), we verified that most cluster marker genes were specific to their corresponding assigned cell groups (Fig. 4b), although a subset of oligodendrocyte and astrocyte subcluster markers had weak detection. In addition, the assignment of glutamatergic cells recapitulated their predicted spatial layer structure (Fig. 4c), and there was concordance among most cluster proportions between snRNA-seq and Xenium (Supplementary Fig. 5), providing further evidence that the ISH data are of reasonable quality for cluster assignment in tissue. We restricted our validation to the snRNA-seq-derived cluster associations described in our original cluster-based analysis (Fig. 1), using the same proportion analysis approach. We confirmed associations between the proportions of *GPNMB*^+^ microglia and *ARHGAP24*^+^ oligodendrocytes and the slope of cognitive decline (see Methods for interpretation of this measure), and between the proportion of *EPHB2*^+^ oligodendrocytes and PHFtau tangle density (Fig. 4d, Supplementary Fig. 5 and Supplementary Table 14). In intact tissue, we also confirmed the lower proportion of *WIF1*^+^*COX8A*^+^ astrocytes and *RORB*^+^*CUX2*^+^*CCDC68*^+^ glutamatergic neurons in Braak stage 3 than in Braak stages 0–2 (Fig. 4e and Supplementary Table 14). Other cell cluster–phenotype associations from our snRNA-seq analysis (Fig. 1) showed trends in the Xenium data but did not reach statistical significance (Fig. 4e and Supplementary Table 14), probably because the number of donors in this validation experiment was lower than that in the snRNA-seq data.

## Agreement with previous studies

To further evaluate the robustness of our findings, we mapped our clusters to two large-scale snRNA-seq studies on the DLPFC, both of which included mainly white individuals from ROSMAP^6,8^. As with the Xenium data, we attempted to validate only significant associations from our cluster-specific analysis (Fig. 1). We found that *GPNMB*^+^ microglia showed the same robust and statistically significant association in the DLPFC in these two published studies, in individuals with a clinical diagnosis of MCI or Alzheimer’s dementia, and with a worse slope of cognitive decline (Fig. 4e and Supplementary Table 15). The proportion of *ARHGAP24*^+^ oligodendrocytes was consistently higher in individuals with a worse slope of cognitive decline in both studies, although this trend did not reach statistical significance (*P* < 0.05). Similarly, the proportion of *RORB*^+^*CUX2*^+^*CCDC68*^+^ neurons was consistently lower in individuals at Braak stages 3 and 4, compared with those at Braak stages 0–2, although this difference was again not statistically significant (Fig. 4e). The reverse mapping of cluster labels from one of the snRNA-seq studies^8^ also showed that putative lipid-processing microglial subgroups Mic.12 and Mic.13—both of which express *GPNMB*—were more abundant in individuals at Braak stage 5 or 6, in individuals with a clinical diagnosis of AD dementia and in individuals with a higher PHFtau tangle density, in all three of our population groups (Fig. 4f). The same association was found for Oli.7 (the analogue of *ARHGAP24*^+^ oligodendrocytes) and Ast.10 (the analogue of W*IF1*^+^*VAV3*^+^ astrocytes (Fig. 4f). Similarly, mapping of vulnerable GABAergic neuron clusters from the other snRNA-seq study^6^ to our dataset revealed that *SST*^+^*MAFB*^+^ neurons showed a trend towards lower proportions in individuals with a higher amyloid-β load and PHFtau tangle density in all three of our population groups, and *CUX2*^+^*MSR1*^+^ neurons showed the same trend in individuals with a higher PHFtau tangle density and steeper slope of cognitive decline (Fig. 4f). Thus, despite the smaller sample size per population group in our study, we find that our key microglial, neuronal and oligodendrocyte findings are consistent with findings from larger—although less diverse—studies.

## Molecular subgroups in individuals with AD

**Fig. 5:**
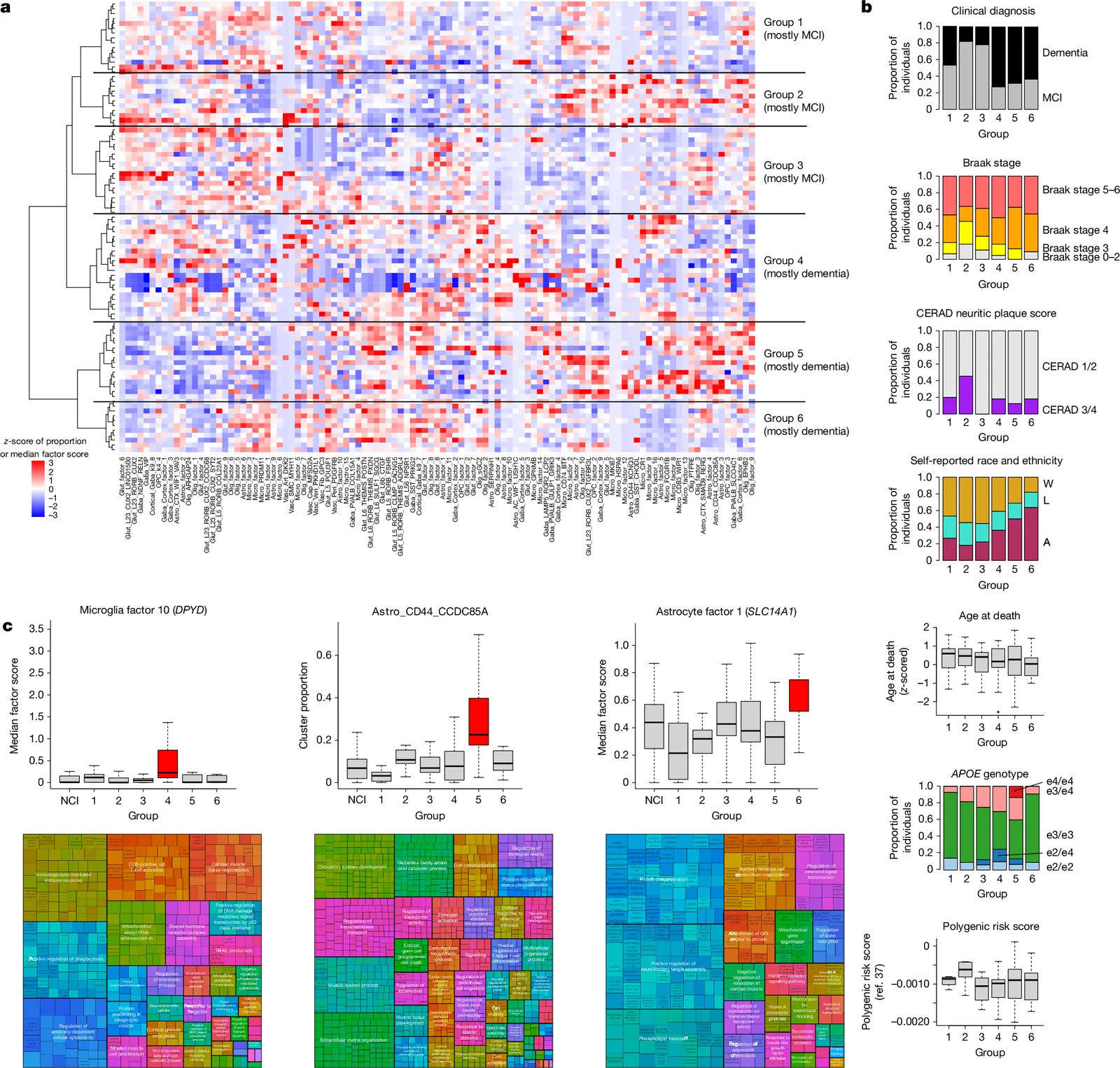
Molecularly defined donor subgroups. **a**, Heat map showing six clusters of individuals (rows) based on the relative proportions of cellular clusters and median factor values (columns). **b**, Bar plots showing the relative composition of each group on the basis of terminal cognitive diagnosis, pathological staging, population group, age at death, *APOE* genotype and polygenic risk score. Although none of the three dementia-related clusters shows significantly different composition across these categories, some trends in the representation of *APOE* e4/e4 donors in group 5 and African American donors in group 6 are evident. **c**, Clusters and factors with higher relative mean representation in group 4 (left), group 5 (middle), and group 6 (right), with corresponding grouped gene ontology (GO) enrichment terms for cluster- or factor-defining genes below. Box and whisker plots show the median (centre line), the IQR (box limits), and whiskers extending to the most extreme values no further than 1.5 times the IQR from the top or bottom of the box limits.

**Extended Data Fig. 5:**
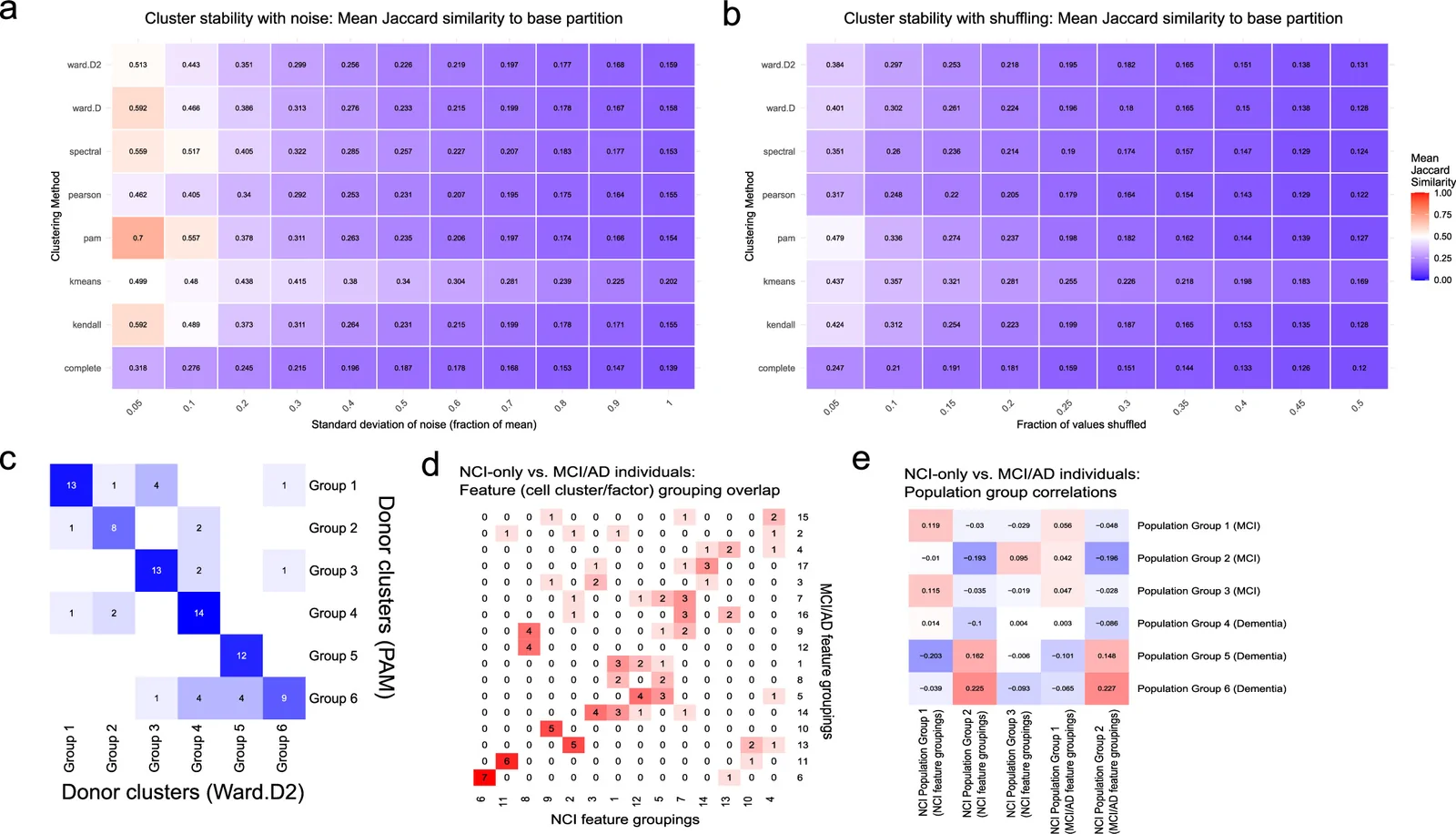
Assessment of the robustness of MCI and AD clustering across methods. **a**, Heat map showing the effect of randomly distributed noise on 8 different clustering workflows. Individual cells represent the Jaccard overlap of the partitioning obtained by each clustering workflow (rows) in the reference partition (zero-noise case) versus partitionings obtained at varying amounts of noise (columns, with each noise level having a standard deviation as a given fraction of the mean for each feature). The 17 agglomerated features are combinations of cluster and factor proportions with correlated values across all the donors. As expected, the Jaccard overlap decreases with higher noise levels, but this decrease is less for the PAM workflow, hierarchical clustering with Ward’s D method and Spearman correlation (“Ward.D”) and hierarchical clustering with Ward’s D method and Kendall rank correlation (“Kendall”). **b**, Same as **a**, but with feature values randomly shuffled instead of being altered with noise. As in **a**, the Jaccard overlap decreases as more values are shuffled, with the same three workflows showing the lowest decrease: PAM, hierarchical clustering with Ward’s D method and Spearman correlation (“Ward.D”) and hierarchical clustering with Ward’s D method and Kendall rank correlation (“Kendall”). **c**, Confusion matrix of donor assignment into clusters using the PAM workflow and the hierarchical clustering with Ward’s D method and Spearman correlation, showing overall agreement in the donor clusters between these two workflows, which also exhibit the greatest stability in the face of noise and shuffling as shown in **a**,**b**. The Ward’s D method and Spearman correlation workflow clusters correspond to the panels in Fig. 5. **d**, Comparison of NCI individual clustering with MCI/AD individual clustering, showing the confusion matrix of de novo clustering of features (cluster proportions and factors) in NCI individuals versus in MCI/AD individuals. Whereas some groupings of cluster proportions/factors are similar across NCI individuals and MCI/AD individuals, most cluster proportions/factors show differential correlations in these two sets of individuals, suggesting that coordinated variation in molecular signatures is different in NCI individuals versus individuals with cognitive impairment. **e**, Overall correlation of mean feature values in MCI/AD population clusters (rows) with NCI population clusters. NCI individuals were clustered in two ways: (1) using de novo groupings of features (cluster proportions/factors) only in NCI individuals, resulting in three NCI groups, and (2) using the groupings of features based on coordinated variation in MCI/AD individuals, resulting in two NCI groups. In both cases, the groups of NCI individuals do not show strong similarities with the MCI/AD groups, based on the correlation of mean feature values. Overall, this suggests that the subgroups of MCI/AD individuals do not reflect heterogeneity found in NCI individuals, but are rather more likely to be distinct molecular subtypes of disease.

Whereas the results described above focus on shared signatures across the entire sampled population, these global association analyses would miss signatures that are present only in subsets of individuals with disease phenotypes. These potential molecular ‘endophenotypes’ might be important when stratifying individuals for clinical or therapeutic studies that aim to modulate dysregulated molecular pathways. Thus, characterizing molecular heterogeneity in individuals with cognitive impairment is a key area, made possible by the profiling of tissue from larger numbers of individuals. Here, we used our multi-ethnic population of donors to analyse the heterogeneity of molecular brain-tissue profiles, similar to previous studies^8,14,40^. We focused on the STG because it was the most profiled region among individuals with MCI and AD in our study, and because it shows signs of AD pathology earlier than the DLPFC does. Using a hierarchical clustering approach based on cluster and factor composition in STG tissue from individuals with MCI or AD, we identified six donor subgroups that were robust to method and parameter selection (Fig. 5a, Extended Data Fig. 5 and Methods). Three of these subgroups were enriched for individuals with clinical diagnoses of MCI, and more than 70% of the individuals in each of the other three subgroups had a clinical diagnosis of AD dementia (Fig. 5b). CERAD scores and Braak stages did not differ across the three MCI-predominant subgroups or across the three AD-dementia-predominant subgroups (Fig. 5b), suggesting that standard pathological measures of disease progression do not distinguish the subgroups within each of these two sets. The small number of *APOE* e4/e4 donors (that is, individuals carrying two copies of the e4 variant of the *APOE* gene) profiled were all in group 5, but the distributions of overall polygenic risk scores were similar across the groups (Fig. 5b). Among the three AD dementia groups, group 6 (*n* = 10) has an absolute majority of African American individuals (*n* = 7), with a trend towards significance (hypergeometric test with FDR-adjusted *P* = 0.081).

Although the three dementia-predominant subgroups did not show substantial differences in disease progression based on CERAD score or Braak stage (Fig. 5b), we identified restricted molecular signature: group 4 was characterized by a higher representation of microglia factor 10 (enriched for phagocytosis genes; Fig. 5c); individuals in group 5 had higher proportions of putative fibrous-like *CD44*^+^ astrocytes (Fig. 5c); and group 6 was distinguished by higher expression of astrocyte factor 1 (enriched for *SLC14A1* and genes involved in tangle assembly and phospholipid transport; Fig. 5c). This grouping suggests that there is a complex landscape of molecular heterogeneity in individuals with dementia, with potentially distinct combinations of cellular signatures. Parallel groupings are not found in donors without cognitive impairment (Extended Data Fig. 5), either with the same clustering of features (from individuals with MCI and AD) or with de novo identification of correlated features (only from NCI individuals). This suggests that these molecular signatures represent endophenotypes associated with cognitive impairment and dementia, rather than population-level natural variation independent of disease status. Finally, despite the stability and robustness of these donor groupings in the current dataset, larger sample sizes are required to establish further generalizability. This work thus complements previous studies^8,14,40^, providing further insight into the molecular heterogeneity in tissues from individuals with Alzheimer’s pathology and dementia.

## Discussion

In studies of ageing and neurodegeneration, including participants from a variety of racial and ethnic backgrounds is important to answer two questions: (1) which disease-associated molecular signatures are shared across different population groups and (2) how do molecular subtypes of disease self-organize when a broad range of individuals are included. Here, by generating single-nucleus snRNA-seq and snATAC-seq data from individuals of various backgrounds, we show that specific signatures in several cell classes—*GPNMB*^+^ microglia, *SERPINH1*^+^ astrocytes, *SST*^+^ GABAergic neurons, a subset of *CUX2*^+^*RORB*^+^ glutamatergic neurons and a range of continuous astrocytic and oligodendrocytic signatures—exhibit similar associations with AD pathology and cognitive phenotypes in African American, white and Latin individuals. A key aspect of this study is the combination of discrete and continuous categorization of cellular heterogeneity within each major cell class. As reported by us and others before, continuous representation of certain glial classes (such as oligodendrocytes) might yield clearer associations with disease phenotypes than discrete cluster-based representation.

With regard to the second question, previous studies have investigated heterogeneity in AD pathology, biofluids and bulk molecular signatures, and our results also point to distinct molecular endophenotypes in subsets of individuals with disease. Whereas these molecular signatures are not found in all individuals with disease, the people who had these signatures were cognitively impaired, which suggests that convergent clinical phenotypes result from the dysregulation of distinct molecular pathways. This is an important consideration for preclinical studies, biomarker identification and patient stratification for therapeutics. We note that this area of the field of AD transcriptomics is still developing, and we view our results as preliminary given the sample size.

The findings summarized above should be considered in the context of three main caveats to this observational study. One of these is the relatively shallow depth of snATAC-seq (median of 30,000 reads per nucleus); indeed, our snATAC-seq data did not reveal any groupings of nuclei beyond those observed in the snRNA-seq data, and we did not identify any shared associations of individual chromatin regions with disease within a cell type across all three population groups after multiple testing correction.

The second caveat in this study is the sample size. Although the total number of tissue samples processed (399) is comparable with previous large-scale single-nucleus studies of AD^6,8,9^, the number of donors per population group is less than 100. In addition, the in-tissue validation experiments using Xenium profiled fewer than 40 donors, resulting in insufficient statistical power to validate some of the snRNA-seq-derived findings. As a result, this study is well-powered to interrogate shared signatures across population groups, but less so to identify group-specific signatures, especially given confounders such as age at death (Extended Data Fig. 1). Thus, findings such as putatively differential chromatin accessibility at the AD-associated locus containing *KANSL1* (Fig. 3d), and the lack of cross-population group reproducibility of vulnerable neuronal signatures identified in previous studies, do not necessarily indicate race- or ethnicity-specific differences, but could rather be a result of the lack of power. The third caveat is that the findings in this study are observational rather than mechanistic. As with all studies on post-mortem tissue, the inferences about cell-type-specific associations are drawn from cross-sectional data, and do not incorporate mechanistic or perturbation analyses. As a result, the main findings point to signatures that should be prioritized for further study in systems allowing for longitudinal examination and perturbations.

It is worth reiterating that we examined self-reported race and ethnicity as well as genetic ancestry in two parallel association analyses. Given that most of these donors lived in the USA from the 1940s onward, it is likely that their perceived race and ethnicity affected their life experiences. Thus, our analysis using self-reported race accounts for both genetic and life-experience effects on brain-tissue signatures. By contrast, our genetic ancestry analysis does not, in theory, include effects of life experience on brain-tissue signatures. However, because our main goal was to identify shared signatures across groups, it is not surprising that the results were mostly consistent across both analyses. Ultimately, further investigation of the shared cell-type signatures highlighted here could lead to the identification of preclinical targets that are relevant across a wide range of racial and ethnic backgrounds. In parallel, our dataset serves as a resource for future studies investigating how genetic variants affect transcriptomic and epigenetic signatures in older individuals. Thus, our work sets the stage for large studies that use a similar representative sampling approach to consider the effects of both genetic heterogeneity and diverse life experience in AD.

## Methods

### Study participants, ethics and clinicopathological characterization

All brain tissue was obtained from participants in the Religious Order Study and Memory and Aging Project (ROSMAP)^19^, the Minority Aging Research Study (MARS)^20^ and the Latino Core Study^21^. As described previously, all participants are without known dementia at enrolment and have annual clinical evaluations; participants whose brain tissue was profiled in this study also consented to brain donation. At death, the brains undergo a quantitative neuropathological assessment, and the participant’s rate of cognitive decline is calculated from the longitudinal cognitive measures, which include up to 31 yearly evaluations^19^. An institutional review board at Rush University Medical Center approved each study, and an institutional review board at Columbia University Irving Medical Center approved the use of the post-mortem tissue samples for molecular analysis. All participants included in the analyses presented here signed an informed consent, Anatomical Gift Act and repository consent. For this study, we selected 167 participants, including all donors from the MARS and Latino Core Study who had full pathological characterization by December 2022, and availability of fresh-frozen tissue from at least two of the three brain regions profiled (DLPFC, STG and AC). As a result, our study cohort includes diverse individuals across the full range of the pathological stages and diagnosis of AD and MCI^41–43^. A subset of demographic and clinicopathological characteristics are summarized in Fig. 1 and Extended Data Fig. 1. Pathological measures were collected as previously described^44,45^. We focused our analysis on the following measures:

1. CERAD neuritic plaque score^42,46^: a semiquantitative measure of neuritic plaques. A neuropathological diagnosis was made of no AD, possible AD, probable AD or definite AD on the basis of semiquantitative estimates of neuritic plaque density as recommended by CERAD, modified to be implemented without adjustment for age and clinical diagnosis. A CERAD neuropathological diagnosis of AD required moderate (probable AD) or frequent (definite AD) neuritic plaques in one or more neocortical regions. Diagnosis includes algorithm and neuropathologist’s opinion, blinded to age and all clinical data. The coded values correspond to 1 (definite), 2 (probable), 3 (possible) and 4 (no AD).
2. Braak stage^42^: a semiquantitative measure of the severity of neurofibrillary tangle (NFT) pathology. The Bielschowsky silver stain was used to visualize NFTs in the frontal, temporal, parietal and entorhinal cortex, and the hippocampus. Braak stages were based on the distribution and severity of NFT pathology: Braak stages 1 and 2 indicate NFTs confined mainly to the entorhinal region of the brain; Braak stages 3 and 4 indicate the involvement of limbic regions such as the hippocampus; and Braak stages 5 and 6 indicate moderate to severe neocortical involvement. Diagnosis includes algorithm and neuropathologist’s opinion.
3. Neuritic plaque counts: a quantified measure determined by microscopic examination of silver-stained slides from five regions: midfrontal cortex, midtemporal cortex, inferior parietal cortex, entorhinal cortex and hippocampus. The count of each region is scaled by dividing by the corresponding standard deviation. The five scaled regional measures are then averaged to obtain a summary measure for neuritic plaque counts.
4. Diffuse plaque counts: a quantitative measure determined by microscopic examination of silver-stained slides from five regions: midfrontal cortex, midtemporal cortex, inferior parietal cortex, entorhinal cortex and hippocampus. The count of each region is scaled by dividing by the corresponding standard deviation. The five scaled regional measures are then averaged to obtain a summary measure for diffuse plaque counts.
5. Amyloid-β load^47^: a quantified measure of amyloid-β protein identified by molecularly specific immunohistochemistry and quantified by image analysis. The value is the percentage area of cortex occupied by amyloid-β, and the overall score is the mean score in eight regions (four or more regions per individual are needed to calculate). The square root of this final score was used for association analyses.
6. PHFtau tangle density^47^: a quantitative measure of neuronal NFTs, identified by molecularly specific immunohistochemistry (antibodies to abnormally phosphorylated tau protein, AT8). Cortical density (per mm^2^) is determined using systematic sampling. Mean of tangle score in eight regions (four or more regions are needed to calculate). The square root of this final score was used for association analyses.
7. Clinical diagnosis^48^: physician’s overall cognitive diagnostic category, based on all available clinical data at the time of death. All available clinical data were reviewed by a neurologist with expertise in dementia, and a summary diagnostic opinion was rendered on the most likely clinical diagnosis at the time of death. Summary diagnoses were made blinded to all post-mortem data. Case conferences including one or more neurologists were used for consensus on selected cases. For this study, the categories used were NCI (no cognitive impairment; no impaired domain), MCI (mild cognitive impairment; one impaired domain) and Alzheimer’s dementia.
8. Slope of cognitive decline: a quantitative measure based on uniform structured clinical evaluations—including a comprehensive cognitive assessment—that are administered annually to the participants and have been summarized in previous publications^49,50^. Scores from 19 cognitive performance tests, 17 of which were used to obtain a summary measure for global cognition, as well as measures for 5 cognitive domains of episodic memory, visuospatial ability, perceptual speed, semantic memory and working memory. The summary measure for global cognition is calculated by averaging the standardized scores of the 17 tests, and the summary measure for each domain is calculated similarly by averaging the standardized scores of the tests specific to that domain. To obtain a measurement of cognitive decline, the annual global cognitive scores are modelled longitudinally with a mixed-effects model, adjusting for age, sex and education, providing person-specific random slopes of decline (which we refer to as the slope of cognitive decline). For association analyses in this study, the negative of the slope value was used (so that higher values of the association variable correspond to steeper slopes of decline).

### Generation of snRNA-seq and snATAC-seq (multiome) data

#### Tissue preparation

This study profiles post-mortem frozen human brain tissue isolated from the DLPFC (BA9), SPG (BA22) and AC, from individuals in the ROSMAP, MARS and Latino Core Study cohorts at Rush University. Tissue from each of these regions was dissected while frozen from flash-frozen tissue blocks at Rush University and sent to Columbia University. Working on ice throughout, we dissected out the white matter and meninges, when present. The following steps were also performed on ice: about 50–100 mg of grey-matter tissue was transferred into a Dounce homogenizer (Sigma, D8938) with 2 ml NP40 lysis buffer (0.1% NP40, 10 mM Tris, 146 mM NaCl, 1 mM CaCl_2_, 21 mM MgCl_2_ and 40 U ml^−1^ of RNAse inhibitor (Takara, 2313B)). Tissue was gently dounced while on ice 25 times with pestle A followed by 25 times with pestle B, then transferred to a 15-ml conical tube. Next, 3 ml of phosphate-buffered saline (PBS) + 0.01% bovine serum albumin (BSA) (NEB, B9000S) and 40 U ml^−1^ of RNAse inhibitor were added for a final volume of 5 ml and then immediately centrifuged with a swing bucket rotor at 500*g* for 5 min at 4 °C. Samples were processed two at a time, the supernatant was removed and the pellets were set on ice to rest while processing the remaining tissues to complete a batch of three samples for each run of the 10x Genomics Chromium platform (see pooling information below and in ‘Data availability’). The nuclei pellets were then resuspended in 500 ml of PBS + 0.01% BSA and 40 U ml^−1^ RNAse inhibitor. Nuclei were filtered through 20-μm pre-separation filters (Miltenyi, 130-101-812) and counted using the Nexcelom Cellometer Vision and AO/PI stain at a 1:1 dilution with a cellometer cell counting chamber (Nexcelom, CHT4-SD100-002).

#### Library preparation and sequencing

To generate snRNA-seq + snATAC-seq (multiome) data, individual samples were pooled into groups of three, with each pool containing (as far as possible) one sample from each of the three regions, and balanced across population groups to minimize batch effects (https://www.synapse.org/Synapse:syn53649030). Approximately 20,000 total nuclei from three samples were pooled into a single sample and these nuclei were run on the 10x Genomics Chromium platform using the 10x multiome protocol (Chromium Next GEM Single Cell Multiome ATAC + Gene Expression Reagent Bundle, PN-1000283). In brief, after transposition, gel beads-in-emulsion (GEMs) were generated by combining barcoded gel beads, transposed nuclei, a master mix that includes reverse transcription (RT) reagents and partitioning oil on a Chromium Next GEM Chip J (10x Genomics; PN-2000264). Incubation of the GEMs in a thermal cycler for 45 min at 37 °C and for 30 min at 25 °C generated full-length cDNA from poly-adenylated mRNA for the gene-expression library and a Spacer sequence that enabled the attachment of barcodes to transposed DNA fragments for the ATAC library. This was followed by a quenching step that stopped the reaction. Next, GEMs were broken, and pooled fractions were recovered. Silane magnetic beads were used to purify the first-strand cDNA from the post-GEM–RT reaction mixture. Barcoded transposed DNA and barcoded full-length cDNA from poly-adenylated mRNA were pre-amplified by PCR and the products were used as input for both ATAC library construction and cDNA amplification for gene-expression library construction. Libraries were pooled and sequenced together on a NovaSeq 6000 with an S4 flow cell (Illumina) at the New York Genome Center, for a target coverage of around 645 million reads per sample for snRNA-seq and 645 million reads per sample for ATAC-seq. The snATAC-seq libraries were sequenced as follows: read 1N, 50 cycles; i7 index, 8 cycles; i5 index, 24 cycles; read 2N, 49 cycles. The snRNA-seq libraries were sequenced as follows: read 1, 28 cycles; i7 index, 10 cycles; i5 index, 10 cycles; read 2, 90 cycles.

#### Primary processing of sequencing data

Raw fastq files were aligned to the genome or transcriptome and quantified using the CellRanger ARC v.2.0.2 package (https://www.10xgenomics.com/support/software/cell-ranger-arc/downloads) with default parameters. snRNA-seq count files were then processed with the CellBender package v.0.2.0^51^ (https://github.com/broadinstitute/CellBender) to remove background signal, also with default parameters. The post-processing CellBender h5 files were then used for downstream analysis and demultiplexing.

#### Demultiplexing of sample barcodes and assignment to donors

Because each multiome library consisted of nuclei from three samples from different donors, the original donor of nuclei contained in each droplet was inferred through single-nucleotide polymorphisms (SNPs) in snRNA-seq reads using the freemuxlet workflow in the popscle package^52^ (http://github.com/statgen/popscle). The pooling of samples was designed such that no pair of individuals was represented in more than one library—this design allowed us to demultiplex and assign nuclei to individuals even without their reference genotype information (multiplexing plan included in the data deposited; see ‘Data availability’). Freemuxlet was run on each 10x Genomics run, with the assumption of three donors (each contributing one sample) in the mixture of nuclei. Finally, the donor-specific VCFs generated by freemuxlet were compared across all batches, allowing us to assign each donor group a unique identity based on the combination of batches it appears in. We then assigned each singlet nucleus to the original donor.

### Clustering of nuclei

To assign nuclei to individual clusters, we focused on the snRNA-seq data, and clustered nuclei using the Seurat v.4 package^53^ (https://satijalab.org/seurat/) from each 10x Genomics batch separately to identify major cell classes: glutamatergic neurons, GABAergic neurons, oligodendrocytes, oligodendrocyte precursor cells (OPCs), microglia, astrocytes, vascular cells, ependymal cells and non-microglial immune cells. Only nuclei with more than 500 UMIs (based on snRNA-seq) and a mitochondrial UMI percentage lower than 5% were retained. We then aggregated nuclei from each of these broad classes from all batches, and subsequently subclustered each broad class separately; this included four rounds in which clusters with mixed signatures were iteratively removed. After data clean-up, nuclei from each broad cell type were clustered over a range of PCs and resolution parameter values, and an optimal clustering was selected on the basis of the Calinski–Harabasz and Davies–Bouldin scores. To assign nomenclature to each cluster, we examined the fraction of expression of each gene and each cluster, and selected the most differential genes, some in combination, to uniquely identify each cluster. The final parameters for each broad cell type were as follows: astrocytes (10 PCs, resolution 0.3); ependymal cells (10 PCs, resolution 0.05); GABAergic neurons (10 PCs, resolution 0.4); glutamatergic neurons (10 PCs, resolution 0.4); microglia (20 PCs, resolution 0.2); oligodendrocytes (10 PCs, resolution 0.05); OPCs (10 PCs, resolution 0.05); vascular cells (10 PCs, resolution = 0.1). After each broad cell type was clustered, subclusters were merged if they did not have at least four genes with abs(log_2_-fold change) > 2 in each direction for every pair of subclusters. For non-microglial immune cells, cells were not clustered, but rather mapped to the HuBMAP Azimuth annotation using the existing interface (https://azimuth.hubmapconsortium.org/). After quality control, iterative clean-up and removal of nuclei with ambiguous or doublet donor assignments, we retained 1,914,581 nuclei for downstream analysis, out of an initial set of 2,967,275 nuclei.

### Associations of cell-type proportions with clinical and pathological traits

To identify statistically significant differences in cluster proportions across various pathological and clinical conditions, we used four methods: three published methods (ANCOM-BC^54^, LINDA^55^ and sccomp^56^) and a quasibinomial regression approach to assess differences in cell cluster proportions (described in more detail below). *P* values and effect sizes plotted in Figs. 1 and 2 are all from ANCOM-BC, with the corresponding values for the other models reported in Supplementary Table 1. For the published methods, we used standard workflows with default settings for all parameters. For the quasibinomial model, we took advantage of the fact that relative cell-type proportions are naturally distributed in the closed interval [0–1]. Therefore, we used logistic regression with a logit link to model response variables in that interval. However, compositional data tend to be enriched with zeroes, depleted of ones and left-skewed. We modelled this with a quasibinomial distribution that uses an extra parameter to account for overdispersion. We also accounted for heteroskedastic residuals using robust standard errors calculated with White’s original method using the R package sandwich. Using the R package performance, we assessed the model assumptions and the influence of outliers. We computed the 95% confidence intervals based on these robust standard errors using the R package broom. Finally, for each model, we ran multiple iterations randomizing the relative cell-type proportions across participants to assess model calibration and the rate of false positives.

For all methods, we modelled only cell types with at least one more observation than the total number of terms in the models being run: “proportion ~ variable_of_interest + age_at_death + sex + pmi” (where pmi denotes post-mortem interval in hours) for pathological traits and “proportion ~ variable_of_interest + age_at_death + sex + pmi + education” for cognitive traits. Cell compositional analyses were done separately for each broad class: glutamatergic neurons (DLPFC and STG only), GABAergic neurons (including only the relevant GABAergic types in each region), microglia, astrocytes, oligodendrocytes and vascular cells. OPCs were not tested, because they did not have any discrete subclusters, and non-microglial immune cells were also excluded, because of low cell numbers.

Finally, for the cell cluster association analysis in the DLPFC replication datasets^6,8^, we used ANCOM-BC on both the original reference cluster calls and the mapped cluster names, with the same associated variables and covariates as in our dataset.

### Meta-analysis across populations

To identify consistent signals across the three population groups, we modelled each group independently and then meta-analysed. For all of our proportion analyses (ANCOM-BC, LINDA, sccomp and the quasibinomial model), we used the package metafor to run a meta-analysis using maximum likelihood or a fixed-effects model with a *t*-test for the coefficients. For a given compositional analysis method, all meta-analysis *P* values were adjusted using the Benjamini–Hochberg method to obtain FDR-adjusted *P* values; these corrections were implemented for each broad cell class, over all combinations of cell cluster (minimum 4, maximum 15) × clinical or pathological trait (11) × region (minimum 2, maximum 3), resulting in batches of 88 to 495 *P* values being adjusted at the same time. We determined that a specific cell-type–phenotype association was shared across all three population groups only if (1) the FDR-adjusted *P* value was less than 0.1; (2) the sign of association was the same in all groups; and (3) the meta-analysis *I*^2^ value was lower than 50%. These criteria had to be met for at least two of the compositional methods (ANCOM-BC, LINDA, sccomp and the quasibinomial model) with the same directionality of effect.

### Identifying cell-type factors through scHPF

To identify factors modelling continuous gene-expression variation, we used the scHPF method^25^ to generate factors within each broad cell class. For GABAergic neurons, we calculated factors separately for the AC and for the cortex (both regions aggregated). For glutamatergic neurons, we calculated factors only for the cortex, given the paucity of glutamatergic neurons in the caudate. For each run of scHPF on each broad cell class, we identified the optimal number of factors by selecting the largest factor set such that no two factors had a gene-weight vector or cell-weight vector correlation greater than 0.75. For the selection of genes to represent each factor for visualization, we selected the five genes with the largest weights, as well as a mean pseudobulked counts per million (CPM) > 10 across all nuclei in the broad class of interest.

For factor associations with AD phenotypes, we calculated the median factor value per donor–region combination, and ran simple linear models with the same general covariates as in the proportion analyses described above: “proportion ~ variable_of_interest + age_at_death + sex + pmi” for pathological phenotypes and “proportion ~ variable_of_interest + age_at_death + sex + pmi + education” for phenotypes that include assessments of cognition. As with the compositional analysis, we ran a fixed-effects meta-analysis across the population groups, adjusted *P* values within each broad class (across all clinical and pathological traits and regions) using the Benjamini–Hochberg method, and report as significant only the factor–trait combinations that passed the following criteria: (1) FDR-adjusted meta-analysis *P* < 0.1; (2) sign of association the same in all population groups; and (3) meta-analysis *I*^2^ < 50%.

### WGCNA

As well as identifying factors using scHPF, we also performed WGCNA^26^, as an alternative method to characterize associations between modules of genes and clinicopathological phenotypes. We used the R package WGCNA v.1.74-5, starting with pseudobulked data (CPM) aggregated per donor and region for seven broad classes of cells. Within each class, we retained only genes with CPM > 10 in at least 5 samples, and identified cell-type-specific soft-threshold powers on the log-transformed CPM matrices: glutamatergic neurons (power = 10), AC GABAergic neurons (power = 12), cortical GABAergic neurons (power = 10), astrocytes (power = 4), oligodendrocytes (power = 6), OPCs (power = 4), microglia (power = 4) and vascular cells (power = 3). All signed modules for each cell type were generated using the blockwiseModules function with default parameters, except for the following: deepSplit=2, minModuleSize=30, maxBlockSize=80000, reassignThreshold=0 and mergeCutHeight=0.3.

After identifying gene modules for each cell type, we assessed the enrichment of each module’s genes with cluster differential genes and gene factor loadings. We used the fgsea package in R to calculate *P* values of enrichment of the genes in a given module in the ranked list of cluster differential genes (for each discrete cluster) and in the ranked list of factor loadings (for each scHPF factor).

All module association analyses with pathological and clinical measures were run in the same way as for the scHPF factors as described above, except that module eigengenes per sample were used instead of median factor values. This includes the same approach for multiple testing correction, and the same criteria for evaluation for meta-analysis *P* values. However, we also included one additional criterion for significance of modules with traits: all meta-analysis *P* values had to be more significant than the lowest *P* value for module 0 eigengene for each cell type. Because module 0 in each cell type does not represent a coherent set of correlated genes, it can be used as a bound to eliminate potential false positives.

### Gene ontology analysis

For enrichment analysis, differentially upregulated genes were identified for each subcluster relative to all other subclusters of the same major cell class using the standard edgeR package workflow, with counts pseudobulked per individual sample (donor + region). Genes were first filtered to include only those with at least 10 CPM in at least 10 pseudobulked samples for the cluster of interest. Genes for enrichment were selected according to the following criteria: (1) Benjamini–Hochberg FDR-adjusted *P* < 0.05; (2) mean pseudobulk CPM ≥ 10 for the cluster of interest; and (3) at least twofold higher expression (log_2_-fold change > 1) in the cluster of interest. Genes that satisfied these criteria were then assessed for ‘biological process’ enrichment using the topGO R package, filtered to those processes with a Fisher’s test *P*_adj_ < 0.1 and aggregated into broader categories using the rrvrgo package.

For factors, genes were filtered to include only those detected in 5% of pseudobulked samples in a given cell class, and with a mean CPM > 5 across all pseudobulked samples. Of these genes, the top 100 were selected for each factor on the basis of their loading scores, and were fed directly into the topGO package for biological process enrichment. Aggregation and visualization were done in the same way as for cell clusters, using the rrvrgo package with pathways selected on the basis of a Fisher’s test *P*_adj_ < 0.1.

### snATAC-seq visualization and differential peak analysis

snATAC-seq peaks were first calculated separately for each batch using CellRanger ARC v.2, as described above. Peaks were then aggregated separately across major RNA-defined cell classes (glutamatergic neurons, GABAergic neurons, astrocytes, oligodendrocytes, OPCs, vascular cells, microglia and other immune cells) after the RNA quality control steps outlined above; this resulted in a different set of peak regions called for each major cell class. Peak aggregation and harmonization across samples within each cell class was done using the Signac library in Seurat. Clustering of nuclei in each major cell class using snATAC-seq data was performed within each RNA-defined major cell class using the standard workflow in Signac (latent semantic indexing for dimensionality reduction, followed by Louvain community detection), but did not identify robust subclusters from the Calinski–Harabasz and Davies–Bouldin indices, so no snATAC-seq subclusters were called. Uniform manifold approximation and projection (UMAP) visualizations for ATAC-seq data and for joint snRNA-seq and ATAC-seq data were performed on a subset of 300,000 nuclei randomly sampled across all populations; peaks were harmonized across all nuclei regardless of major cell class. UMAPs for snRNA-seq were based on principal component analysis for dimensionality reduction (30 PCs used); UMAPs for ATAC-seq were based on latent semantic indexing for dimensionality reduction after harmonizing peak calls across the 300,000 nuclei; and UMAPs for joint snRNA-seq and snATAC-seq were based on joint clustering using the WNN workflow in the Signac^57^ R package.

Differential snATAC-seq occupancy among RNA-defined subclusters was measured using the DESeq2^58^ R package on pseudobulked snATAC-seq data. Pseudobulk counts were generated by summing counts for all peak regions within each donor + region sample for that subcluster, after harmonization of peaks within each major cell class. DESeq2 was run for each subcluster (against pseudobulked samples from all other subclusters within the same major cell class) with batch, donor post-mortem interval and donor age at death as covariates. All *P* values were adjusted using the Benjamini–Hochberg FDR correction. For visualizing the top two peaks per subcluster (Fig. 3a), peaks were sorted by *P* value, and the top two peaks with positive log_2_-transformed fold change were selected.

For transcription-factor-binding site and motif enrichment analysis (Fig. 3b), all cluster-specific differential peaks with FDR-adjusted *P* < 0.05 and positive log_2_-transformed fold changes were inputted into the XSTREME algorithm in the MEME suite using the interactive submission mode (https://meme-suite.org/meme/doc/xstreme.html). Transcription factors with enriched binding sites in each subcluster were then selected after filtering out sequence motifs with more than eight repetitions of the same nucleotide and filtering out sequences found in more than one subcluster.

### Open chromatin quantification for AD risk loci

snATAC-seq peak counts were pseudobulked as described above, but for all nuclei from a donor + region sample belonging to each major cell class (instead of each subcluster, as in the description above). Pseudobulked values were then normalized to CPM within each donor + region + cell class sample. For each AD risk locus, CPM counts for all peaks overlapping with the locus (even partially, at the start and end points of the locus) were summed together for each sample. To allow for relative differences in peak locations and widths across different major cell class, these summed CPM values were then divided by the total length of peak regions, and then the mean length-normalized CPM value was calculated for each population group for all samples of a given region and major cell class. Finally, for plotting, the population mean length-normalized CPM value for each locus was divided by the maximum value (across all major cell classes and population groups). The values presented in Fig. 3c therefore underwent four transformations: (1) sample-specific normalization for total read count; (2) length normalization to account for total peak length within a locus; (3) mean calculation and aggregation for each of our three population groups; and (4) normalization to the maximum value for a given locus, to allow for visualization of loci simultaneously.

To assess differential peak occupancy across population groups in each broad cell class, we performed a Kruskal–Wallis non-parametric test on donor-level pseudobulked CPM values for each locus (Supplementary Table 10). Raw *P* values were adjusted for multiple testing within each broad cell class using the Bonferroni correction. Post-hoc pairwise test *P* values were then calculated using Mann–Whitney non-parametric tests between population groups; these post-hoc values were not further adjusted.

### Xenium multiplexed ISH experiments

The Xenium In Situ technology uses targeted panels to detect gene expression. Here, we used a custom gene list based on cell cluster markers from snRNA-seq (for a full list of genes on the panel see Supplementary Table 2). The probes were designed to contain two complementary sequences that hybridize to the target RNA and a third region encoding a gene-specific barcode so that the paired ends of the probe bind to the target RNA and ligate to generate a circular DNA probe. If the probe experiences an off-target binding event, ligation should not occur, suppressing off-target signals and ensuring high specificity. The Xenium workflow (using in-development chemistry and a prototype instrument and consumables) began by sectioning 10-μm fresh-frozen tissue sections onto a Xenium slide, followed by permeabilization to make the mRNA accessible. The mRNAs were targeted by the probes described above and two negative controls: (1) probe controls to assess non-specific binding and (2) genomic DNA controls to ensure that the signal is from RNA. Probe hybridization occurred at 50 °C overnight with a probe concentration of 10 nM. After stringency washing to remove unhybridized probes, probes were ligated at 37 °C for 2 h. During this step, a rolling circle amplification (RCA) primer was also annealed. The circularized probes were then enzymatically amplified (for 1 h at 4 °C followed by 2 h at 37 °C), generating multiple copies of the gene-specific barcode for each RNA-binding event, resulting in a strong signal-to-noise ratio. After washing, background fluorescence was quenched chemically. The biochemistry is designed to mitigate autofluorescence, which is a known issue owing to the presence of lipofuscins, elastin, collagen and red blood cells, and owing to formalin fixation itself. Sections were placed into an imaging cassette to be loaded onto the Xenium Analyzer instrument. The Xenium Analyzer is fully automated and includes an imager (imageable area of about 12 × 24 mm per slide), sample handling, liquid handling, wide-field epifluorescence imaging, capacity for two slides per run and an on-instrument analysis pipeline. The imager is a fast area scan camera featuring a high numerical aperture, a low read noise sensor and approximately 200 nm per pixel resolution. On the Xenium Analyzer, image acquisition was performed in cycles. The reagents, including fluorescently labelled probes for detecting RNA, were automatically cycled in, incubated, imaged and removed by the instrument. After the binding of fluorescent oligos to the amplified barcode sequence, the sample underwent 15 rounds of fluorescent probe hybridization, imaging and probe removal. The *z*-stacks were taken with a 0.75-μm step size across the entire tissue thickness. The Xenium Analyzer captured a *z*-stack of images every cycle and in every channel, which needed to be processed and stitched to build a spatial map of the transcripts across the tissue section. Stitching was performed on the DAPI image, taking all of the stacks from different fields of view (FOVs) and colours to create a complete three-dimensional (3D) morphology image (morphology.ome.tif) for each of the stained regions. First, the lens distortion in internal sensor data was corrected on the basis of instrument calibration data, which were collected to characterize the optical system and were saved on the instrument. Next, the *z*-stacks from the internal sensor data were further subsampled to a 3-μm step size, which was determined empirically to be a useful resolution for cell segmentation quality. Image features were then extracted from the regions where FOVs overlapped. Feature matching was performed to estimate the offsets between adjoining FOVs. The offsets were used to ensure consistent global alignment across the image. Finally, the 3D DAPI image volumes (*z*-stacks) generated across FOVs were stitched together. RCA product image processing was then applied to detect and filter puncta and correct distortion. A punctum is a point source in microscopy that is smaller than a pixel and is measured in units of observed photons. The 3D image volumes (*z*-stacks) obtained for each FOV were processed, for 4 colour channels and 15 cycles, to detect the puncta in 3D space that correspond to labelled RCA products. The RNA fluorescence images were scanned for punctum signals that stood out from the local background. The *XYZ* coordinates of each punctum were refined by examining the local brightness. The signal intensity of the punctum was determined by fitting a Gaussian distribution to the observed emitted light to determine the centre, size and intensity of the point sources. We filtered out puncta that were unlikely to be from true RCA products (non-punctate or low-quality signals). Similar to DAPI images, curvature distortion was corrected. To proceed from puncta to transcripts, decoding was performed using a Xenium codebook—a collection of code words that were assigned to genes in the gene panel. Each code word was defined on the basis of an expected pattern of fluorescent signals recorded across channels and cycles. Some code words were reserved for negative controls. The fluorescent signals from all channels and cycles were compared with the codebook using a global (across all FOVs) maximum likelihood approach. This approach considered attributes such as puncta locations, their colour and cycle of detections and signal intensities. To assign mRNA transcripts to cells, we used the default segmentation parameters in Xenium Onboard Analysis (XOA) v.3.0, which segments cells on the basis of DAPI staining, with an expansion distance of 5 µm or until another cell boundary is encountered.

### Analysis of Xenium data

Processing and downstream analysis of the Xenium counts matrix was performed using the Seurat v.5 package in R, as follows:

1. For each section, all segmented objects (cells) with fewer than 20 total transcript counts were removed.
2. Labels derived from snRNA-seq clusters were then used to generate a set of reference profiles for label transfer. snRNA-seq counts were pseudobulked (raw counts summed) for all clusters per donor for every DLPFC sample (excluding the AC-specific clusters) and pseudobulk profiles with more than 5,000 total counts were retained. These pseudobulked data were then used as the reference for label transfer.
3. The RCTD workflow^39^ was run on the Xenium data, using the pseudobulked snRNA-seq-derived profiles. This workflow, implemented in Seurat v.5, consisted of the spatialRNA, create.RCTD (UMI_min = 20) and run.RCTD (doublet_mode = ”doublet”) commands.
4. Transferred labels were assessed by plotting cluster marker expression per cluster (Fig. 4b), with notes made as to which transferred clusters did not show the expected expression of markers.
5. For each Xenium sample, all objects within the grey matter were manually assigned by drawing boundaries around the grey matter using the gatepoints package in R. Grey–white matter boundaries were identified using cellular (segmented object) density and expression of *MOG* and *RBFOX3*.
6. For validation of snRNA-seq-derived proportion analyses, ANCOM-BC was run in the same way as for the snRNA-seq data, but using cell cluster proportions in the grey matter of the Xenium sections.

### Mapping cell cluster labels across studies

Cluster calls from this dataset were mapped to nuclei from two previously publisheddatasets^6,8^ using the MapMyCells package from the Allen Institute for Brain Science (https://portal.brain-map.org/atlases-and-data/bkp/mapmycells). Nuclei from this study were randomly assigned to five groups, and a separate MapMyCells model was built for each set of nuclei. The nuclei from the query datasets^6,8^ were then assigned five cluster labels, one from each of the five models. Nuclei that received the same label from at least four of the five models were assigned that cluster label, and nuclei that did not meet this criterion were called ‘ambiguous’ and not retained for downstream analysis. This same procedure was also run in the other direction, for mapping cluster names from these two datasets (as reference) onto this study (as query).

### Donor clustering based on cell subcluster frequencies and median factor scores

To identify potential subgroups of donors on the basis of cell-type profiles, the within-broad-class normalized proportions of astrocyte, neuronal, oligodendrocyte, microglial and vascular clusters, as well as the median factor values per donor, were concatenated into input vectors for hierarchical clustering approach, with 1 − Pearson’s correlation *r* value as the distance measure and Ward’s method for linkage. The resulting hierarchical tree was cut at various heights, and silhouette scores were calculated at each height. The median silhouette score at each cut height was used to determine that six groupings was optimal. For each of these six groups, the number of donors for each cognitive diagnosis, Braak stage, CERAD score and population group were calculated, as shown in Fig. 5a.

We evaluated whether the clustering partitions acquired using hierarchical clustering with Ward’s D method for linkage and dissimilarity through Spearman correlation were generalizable to (1) minor changes to the initial dataset or (2) the clustering method used. We evaluated the robustness of eight algorithms: three that used different correlation metrics ((1) Pearson, (2) Spearman and (3) Kendall) but which retained the use of hierarchical clustering with Ward’s D, and five that did not use Ward’s D but which retained the calculation of dissimilarity with Spearman correlation ((4) hierarchical clustering with Ward’s D2 method, (5) hierarchical clustering with complete linkage, (6) spectral clustering, (7) *k*-means clustering and (8) partitioning around medoids (PAM) clustering).

We first tested all eight clustering algorithm variations on the initial, unperturbed datasets (consisting of the 17 agglomerated features) to create reference partitions per method. We then performed two robustness analyses. In the first analysis, we added varying amounts of randomly distributed noise centred around a mean of 0 into the 17 agglomerated features. Noise levels ranged from 0.05 to 1 standard deviations of each feature. We tested 100 bootstrap iterations for each noise level and method, and calculated the Jaccard similarity of each bootstrap iteration to the reference partition for that method to create a distribution of Jaccard similarity scores. Across methods, we found that added noise leads to decreased similarity, which was expected. In the second analysis, we shuffled a percentage of each of the values per column across samples. As expected, a higher shuffling percentage resulted in lower similarity to the reference partition. Across both analyses, the PAM method exhibited the highest Jaccard similarity compared with the base partition. We were interested in whether the reference PAM partition corresponded to cognitive diagnoses, and found similar results to those obtained from the reference Ward D cluster assignments. Finally, to qualitatively evaluate whether the reference PAM partition differed substantially from the reference Ward D partition, we plotted river plots showing cluster assignments between the two partitions. We found a large amount of overlap, because many individuals in the same group in one partition were also grouped together in the other.

### Genetic ancestry estimation and modelling

To discern the effects of environment and genetics on the associations between AD traits and our molecular features, we used available WGS data from 151 of our 167 donors. The remaining 16 donors belonged to the white racial and ethnic group; this was the largest group, and thus the results should not be affected by their absence. For the Latin and African American groups, we accessed the gatk-Haplotype-called gvcf files on GRCh38, then used GenomicsDBImport to generate jointly genotyped GenDB files, and finally kept only the varying regions using GenotypeGVCFs to generate multi-sample VCFs per chromosome. We then used bcftools (v.1.23) to select only biallelic SNPs and PLINK (v.1.9.0-b.8) to remove variants with more than 2% missing data, exclude rare variants with a minor allele frequency lower than 1% and exclude individuals with more than 5% of their genotypes missing. In addition, we discarded variants that deviated significantly from Hardy–Weinberg equilibrium (*P* < 1 × 10^−6^). We then merged the PLINK quality-controlled files for the white group (83,222,307 variants) with those for the Latin and African American groups (14,669,023 variants), yielding a final variant count of 12,601,138 unique and shared SNP IDs. Finally, we performed linkage disequilibrium (LD) pruning on the multi-ethnic PLINK files to exclude variants in LD (*r*^2^ > 0.2) within a 200-SNP window, advancing by 50 SNPs, for a total of 1,355,673 variants.

Using those quality-controlled multi-ethnic variants, we computed genomic PCs using PLINK and ADMIXTURE (v.1.3.0) for 1 < *k* < 9. Admixture loadings and genomic PCs were plotted against all AD traits to assess their correlations. The first two genomic PCs capture mostly racial and ethnic variation (Extended Data Fig. 3), whereas PCs 3 and 4 show no clear patterning with the variables of interest. For ADMIXTURE, *k* = 3 showed the biggest changes in log-likelihood, normalized ancestry entropy and minimum pairwise Fst (Extended Data Fig. 3). On the basis of these biological metrics and the complementary numerical optimizations, *k* = 3 was selected for modelling.

For admixture component-based modelling, we ran the same set of meta-analyses (using all four compositional methods and one factor association approach) using the majority admixture component per individual as the grouping variable, instead of self-reported race and ethnic group. The same criteria were used as described above for multiple testing correction and for assessing the robustness of results across methods.

For genetic PC-based modelling, we included the continuous values for PC1 and PC2 as interaction terms with the clinical or pathological trait of interest in two separate models (one for PC1 and one for PC2). No meta-analysis was needed in this case, but multiple testing correction was performed using the same criteria above. Here, we identified cluster and factor associations with a statistically significant main effect (with the trait of interest, FDR-adjusted *P* < 0.1) and non-significant interaction effect with the genetic PC (FDR-adjusted *P* > 0.1). As in our other analyses, cluster associations had to be significant after multiple testing correction in at least two out of four compositional methods.

### Polygenic risk scoring

We computed polygenic risk scores per donor using the most recent and well-powered AD summary statistics^37^ in conjunction with the quality-controlled (but not LD pruned) multi-ethnic PLINK file described in the previous section. To account for variants in LD, we first aggregated variants within 250 kb of the lead variant (nominal *P* < 1 × 10^−5^) with *r*^2^ > 0.1 using PLINK. The effect of these variants was then summed and averaged to score each donor using PLINK.

### Reporting summary

Further information on research design is available in the Nature Portfolio Reporting Summary linked to this article.

## Online content

Any methods, additional references, Nature Portfolio reporting summaries, source data, extended data, supplementary information, acknowledgements, peer review information; details of author contributions and competing interests; and statements of data and code availability are available at https://doi.org/10.1038/s41586-026-10793-0.

## Supplementary information

## Data Availability

All raw data (fastq files) and processed data (post-quality control count tables and cluster identities) are available through the AD Knowledge Portal in Synapse at https://doi.org/10.7303/syn53649093. Batch processing and pooling experimental plan files are available through the AD Knowledge Portal at https://www.synapse.org/Synapse:syn53649030. These files are available by submitting a Data Use Agreement through Synapse, requesting access to AMP AD data. Raw and processed WGS data used for genetic ancestry and polygenic risk scores are available through the AD Knowledge Portal (https://www.synapse.org/Synapse:syn52563603 and referenced in a previous study^59^. A browsable interface to examine the data is available at https://vmenon.shinyapps.io/ampad2_snrnaseq.
