# Supplementary material for: Cell-type signatures of Alzheimer’s disease shared across population groups
Source: Nature. 2026 Jul 15;657(8130):191–201. doi: 10.1038/s41586-026-10793-0 (PMC13538069; doi:10.1038/s41586-026-10793-0)
Supplement: Supplementary file 1 [file 41586_2026_10793_MOESM2_ESM.pdf]

nature portfolio

Corresponding author(s): V. Menon

Last updated by author(s): 6 June 2026

Reporting Summary

Nature Portfolio wishes to improve the reproducibility of the work that we publish. This form provides structure for consistency and transparency in reporting. For further information on Nature Portfolio policies, see our Editorial Policies and the Editorial Policy Checklist.

Statistics

For all statistical analyses, confirm that the following items are present in the figure legend, table legend, main text, or Methods section.

n/a	Confirmed	
☐	☒	The exact sample size (*n*) for each experimental group/condition, given as a discrete number and unit of measurement
☐	☒	A statement on whether measurements were taken from distinct samples or whether the same sample was measured repeatedly
☐	☒	The statistical test(s) used AND whether they are one- or two-sided<br>*Only common tests should be described solely by name; describe more complex techniques in the Methods section.*
☐	☒	A description of all covariates tested
☐	☒	A description of any assumptions or corrections, such as tests of normality and adjustment for multiple comparisons
☐	☒	A full description of the statistical parameters including central tendency (e.g. means) or other basic estimates (e.g. regression coefficient) AND variation (e.g. standard deviation) or associated estimates of uncertainty (e.g. confidence intervals)
☐	☒	For null hypothesis testing, the test statistic (e.g. *F*, *t*, *r*) with confidence intervals, effect sizes, degrees of freedom and *P* value noted<br>*Give P values as exact values whenever suitable.*
☒	☐	For Bayesian analysis, information on the choice of priors and Markov chain Monte Carlo settings
☒	☐	For hierarchical and complex designs, identification of the appropriate level for tests and full reporting of outcomes
☐	☒	Estimates of effect sizes (e.g. Cohen's *d*, Pearson's *r*), indicating how they were calculated

Our web collection on statistics for biologists contains articles on many of the points above.

Software and code

Policy information about availability of computer code

Data collection	10x Genomics Cellranger Arc v2.0.2
Data analysis	R version 4.0.2, Seurat R package version 4, python 3.7, python schpf package version _p37, WGCNA R package version 1.74-5, Cellbender package version 0.2.0, bcftools version 1.23, PLINK version 1.9.0-b.8

For manuscripts utilizing custom algorithms or software that are central to the research but not yet described in published literature, software must be made available to editors and reviewers. We strongly encourage code deposition in a community repository (e.g. GitHub). See the Nature Portfolio guidelines for submitting code & software for further information.

Data

Policy information about availability of data

All manuscripts must include a data availability statement. This statement should provide the following information, where applicable:

- Accession codes, unique identifiers, or web links for publicly available datasets
- A description of any restrictions on data availability
- For clinical datasets or third party data, please ensure that the statement adheres to our policy

All raw (fastq files) and processed data (post-QC counts tables and cluster identities) are available on the AD Knowledge Portal, under the following DOI: https://doi.org/10.7303/syn53649093. Batch processing/pooling experimental plan files are available on the AD Knowledge Portal at https://www.synapse.org/Synapse:syn53649030, Raw and processed whole-genome sequencing used for genetic ancestry and polygenic risk scores are available through the AD Knowledge

Portal (https://www.synapse.org/Synapse:syn52563603 and referenced in Leung et al.[79]). A browsable interface to examine the data is available at https://vmenon.shinyapps.io/ampad2_snrnaseq.

Research involving human participants, their data, or biological material

Policy information about studies with human participants or human data. See also policy information about sex, gender (identity/presentation), and sexual orientation and race, ethnicity and racism.

Reporting on sex and gender	Sex was based on self-reported information from the cohorts at Rush University (ROSMAP, MARS, and Latin Core participants), and was treated as a covariate in all analysis. No sex-specific analyses were performed in this study.
Reporting on race, ethnicity, or other socially relevant groupings	Race and ethnicity were self-reported from the cohorts at Rush University (ROSMAP, MARS, and Latin Core). Race encompassed 4 categories: White, African-American, Asian/Pacific Islander, or other. Ethnicity encompassed 2 categories: Latin and Non-Latin. All analyses were run across three non-overlapping groups: Non-Latin Whites, Non-Latin African-American, and Latin of any race. None of the Latin Individuals self-reported as being African-American by race. Age of death values >90 have been redacted/removed in all figures.
Population characteristics	Additional demographic characteristics used as covariates were age of death, years of education, and post-mortem interval
Recruitment	Participants were recruited at Rush University as part of community cohorts in the Chicago area (MAP, MARS, Latin Core) and nationwide Religious Orders (ROS). Participants had to be 65+ years of age and free of dementia at recruitment. These criteria result in a known skew of the population towards physiologically healthier individuals without early-onset cognitive symptoms, as well as some skew towards more educated individuals, as described in publications on the Rush University cohorts.
Ethics oversight	An Institutional Review Board of Rush University Medical Center approved each study, and an Institutional Review Board at Columbia University Irving Medical Center approved the use of the post-mortem tissue samples for molecular analysis. All participants included in the analyses presented here signed an informed consent, Anatomic Gift Act and repository consent.

Note that full information on the approval of the study protocol must also be provided in the manuscript.

Field-specific reporting

Please select the one below that is the best fit for your research. If you are not sure, read the appropriate sections before making your selection.

[x] Life sciences [ ] Behavioural & social sciences [ ] Ecological, evolutionary & environmental sciences

For a reference copy of the document with all sections, see nature.com/documents/nr-reporting-summary-flat.pdf

Life sciences study design

All studies must disclose on these points even when the disclosure is negative.

Sample size	Sample size was determined by the availability of well-characterized (pathological staining and fully diagnosed) post-mortem brain tissue from individuals of each population group. No pre-study sample size calculation was performed. A total of 399 samples were analyzed, coming from 167 distinct individuals.
Data exclusions	Nuclei not passing QC criteria (<500 genes detected or with >5% mitochondrial UMIs) were excluded from all downstream analyses.
Replication	Replication was performed: 1) experimentally on a subset of brain tissue from the same individuals using the Xenium ISH platform, and 2) analytically using publicly available data from the ROSMAP cohorts, published in Green et al. (2024) and Mathys et al. (2023).
Randomization	Individuals were not randomized in terms of racial and ethnic groups, but were incorporated into a meta analysis across the three groups. Covariates included age at death, sex, years of education (for cognitive associations), and post-mortem interval. Age at death, in particular, showed differences across the three population groups, and so the analysis of all individuals in a randomized fashion would have resulted in loss of signal due to the confounding of age and population group.
Blinding	All investigator were blinded to the individual demographics during data generation, analysis, and QC, as only access to the unique individual project ID was allowed. For association analysis with clinical and pathological phenotypes, investigators were aware of the race and ethnicity of each individual in order to carry out stratified analyses.

Reporting for specific materials, systems and methods

We require information from authors about some types of materials, experimental systems and methods used in many studies. Here, indicate whether each material, system or method listed is relevant to your study. If you are not sure if a list item applies to your research, read the appropriate section before selecting a response.

Materials & experimental systems

n/a	Involved in the study	
☒	☐	Antibodies
☒	☐	Eukaryotic cell lines
☒	☐	Palaeontology and archaeology
☒	☐	Animals and other organisms
☒	☐	Clinical data
☒	☐	Dual use research of concern
☒	☐	Plants

Methods

n/a	Involved in the study	
☒	☐	ChIP-seq
☒	☐	Flow cytometry
☒	☐	MRI-based neuroimaging

Plants

Seed stocks	N/A
Novel plant genotypes	N/A
Authentication	N/A
