# Supplementary material for: Cell-type signatures of Alzheimer’s disease shared across population groups
Source: Nature. 2026 Jul 15;657(8130):191–201. doi: 10.1038/s41586-026-10793-0 (PMC13538069; doi:10.1038/s41586-026-10793-0)
Supplement: Supplementary file 2 [file 41586_2026_10793_MOESM3_ESM.pdf]

Supplementary Fig. 1

Effect of QC parameters on proportion associations

Visualization of effect sizes and p-values of ANCOM-BC model cluster proportion associations (top half) and factor associations (bottom half) across a range of parameter values for the UMI threshold and the thresholded difference in likelihood between a singlet and doublet (sngdbldiff) using the freemuxlet package. Row labels indicate the combination of brain region, cell cluster, and phenotype of interest; these include only the significant combinations outlined in Figure 1. Column labels indicate the singlet threshold (where -Infinity is the default), UMI threshold, and total number of nuclei retained after applying these thresholds. Note that UMI=0 corresponds to the default thresholding of 500 UMIs, so the first column shows values reported in the main figure.

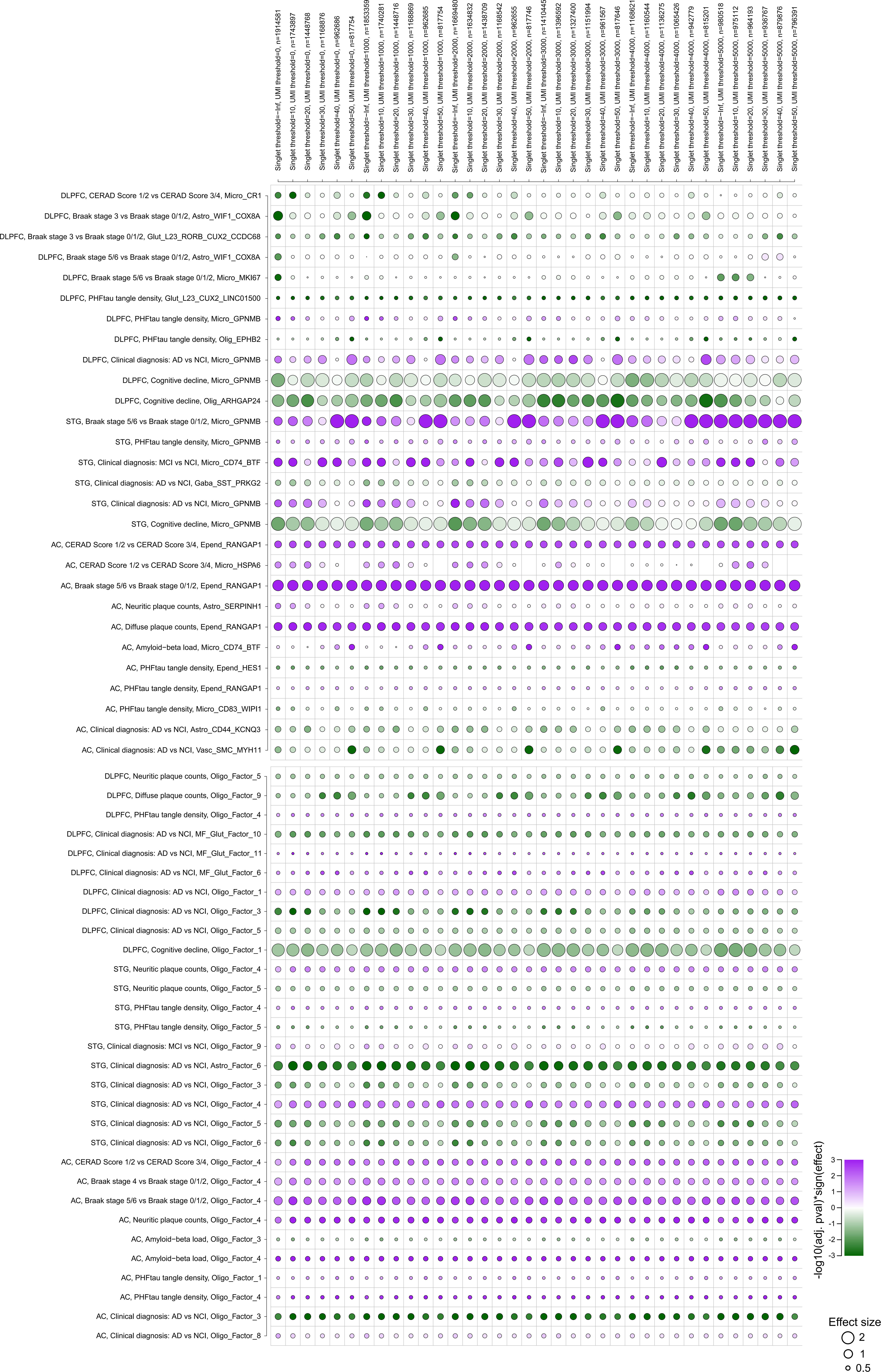

Singlet threshold=−Inf, UMI threshold=0, n=1914581
Singlet threshold=10, UMI threshold=0, n=1743897
Singlet threshold=20, UMI threshold=0, n=1448768
Singlet threshold=30, UMI threshold=0, n=1168876
Singlet threshold=40, UMI threshold=0, n=962686
Singlet threshold=50, UMI threshold=0, n=817754
Singlet threshold=−Inf, UMI threshold=1000, n=1853359
Singlet threshold=10, UMI threshold=1000, n=1740281
Singlet threshold=20, UMI threshold=1000, n=1448716
Singlet threshold=30, UMI threshold=1000, n=1168869
Singlet threshold=40, UMI threshold=1000, n=962685
Singlet threshold=50, UMI threshold=1000, n=817754
Singlet threshold=−Inf, UMI threshold=2000, n=1669480
Singlet threshold=10, UMI threshold=2000, n=1634832
Singlet threshold=20, UMI threshold=2000, n=1438709
Singlet threshold=30, UMI threshold=2000, n=1168542
Singlet threshold=40, UMI threshold=2000, n=962655
Singlet threshold=50, UMI threshold=2000, n=817746
Singlet threshold=−Inf, UMI threshold=3000, n=1410445
Singlet threshold=10, UMI threshold=3000, n=1396592
Singlet threshold=20, UMI threshold=3000, n=1327400
Singlet threshold=30, UMI threshold=3000, n=1151994
Singlet threshold=40, UMI threshold=3000, n=961567
Singlet threshold=50, UMI threshold=3000, n=817646
Singlet threshold=−Inf, UMI threshold=4000, n=1168621
Singlet threshold=10, UMI threshold=4000, n=1160544
Singlet threshold=20, UMI threshold=4000, n=1136275
Singlet threshold=30, UMI threshold=4000, n=1065426
Singlet threshold=40, UMI threshold=4000, n=942779
Singlet threshold=50, UMI threshold=4000, n=815201
Singlet threshold=−Inf, UMI threshold=5000, n=980518
Singlet threshold=10, UMI threshold=5000, n=975112
Singlet threshold=20, UMI threshold=5000, n=964193
Singlet threshold=30, UMI threshold=5000, n=936767
Singlet threshold=40, UMI threshold=5000, n=879876
Singlet threshold=50, UMI threshold=5000, n=796391
DLPFC, CERAD Score 1/2 vs CERAD Score 3/4, Micro_CR1
DLPFC, Braak stage 3 vs Braak stage 0/1/2, Astro_WIF1_COX8A
DLPFC, Braak stage 3 vs Braak stage 0/1/2, Glut_L23_RORB_CUX2_CCDC68
DLPFC, Braak stage 5/6 vs Braak stage 0/1/2, Astro_WIF1_COX8A
DLPFC, Braak stage 5/6 vs Braak stage 0/1/2, Micro_MKI67
DLPFC, PHFtau tangle density, Glut_L23_CUX2_LINC01500
DLPFC, PHFtau tangle density, Micro_GPNMB
DLPFC, PHFtau tangle density, Olig_EPHB2
DLPFC, Clinical diagnosis: AD vs NCI, Micro_GPNMB
DLPFC, Cognitive decline, Micro_GPNMB
DLPFC, Cognitive decline, Olig_ARHGAP24
STG, Braak stage 5/6 vs Braak stage 0/1/2, Micro_GPNMB
STG, PHFtau tangle density, Micro_GPNMB
STG, Clinical diagnosis: MCI vs NCI, Micro_CD74_BTF
STG, Clinical diagnosis: AD vs NCI, Gaba_SST_PRKG2
STG, Clinical diagnosis: AD vs NCI, Micro_GPNMB
STG, Cognitive decline, Micro_GPNMB
AC, CERAD Score 1/2 vs CERAD Score 3/4, Epend_RANGAP1
AC, CERAD Score 1/2 vs CERAD Score 3/4, Micro_HSPA6
AC, Braak stage 5/6 vs Braak stage 0/1/2, Epend_RANGAP1
AC, Neuritic plaque counts, Astro_SERPINH1
AC, Diffuse plaque counts, Epend_RANGAP1
AC, Amyloid−beta load, Micro_CD74_BTF
AC, PHFtau tangle density, Epend_HES1
AC, PHFtau tangle density, Epend_RANGAP1
AC, PHFtau tangle density, Micro_CD83_WIPI1
AC, Clinical diagnosis: AD vs NCI, Astro_CD44_KCNQ3
AC, Clinical diagnosis: AD vs NCI, Vasc_SMC_MYH11
DLPFC, Neuritic plaque counts, Oligo_Factor_5
DLPFC, Diffuse plaque counts, Oligo_Factor_9
DLPFC, PHFtau tangle density, Oligo_Factor_4
DLPFC, Clinical diagnosis: AD vs NCI, MF_Glut_Factor_10
DLPFC, Clinical diagnosis: AD vs NCI, MF_Glut_Factor_11
DLPFC, Clinical diagnosis: AD vs NCI, MF_Glut_Factor_6
DLPFC, Clinical diagnosis: AD vs NCI, Oligo_Factor_1
DLPFC, Clinical diagnosis: AD vs NCI, Oligo_Factor_3
DLPFC, Clinical diagnosis: AD vs NCI, Oligo_Factor_5
DLPFC, Cognitive decline, Oligo_Factor_1
STG, Neuritic plaque counts, Oligo_Factor_4
STG, Neuritic plaque counts, Oligo_Factor_5
STG, PHFtau tangle density, Oligo_Factor_4
STG, PHFtau tangle density, Oligo_Factor_5
STG, Clinical diagnosis: MCI vs NCI, Oligo_Factor_9
STG, Clinical diagnosis: AD vs NCI, Astro_Factor_6
STG, Clinical diagnosis: AD vs NCI, Oligo_Factor_3
STG, Clinical diagnosis: AD vs NCI, Oligo_Factor_4
STG, Clinical diagnosis: AD vs NCI, Oligo_Factor_5
STG, Clinical diagnosis: AD vs NCI, Oligo_Factor_6
AC, CERAD Score 1/2 vs CERAD Score 3/4, Oligo_Factor_4
AC, Braak stage 4 vs Braak stage 0/1/2, Oligo_Factor_4
AC, Braak stage 5/6 vs Braak stage 0/1/2, Oligo_Factor_4
AC, Neuritic plaque counts, Oligo_Factor_4
AC, Amyloid−beta load, Oligo_Factor_3
AC, Amyloid−beta load, Oligo_Factor_4
AC, PHFtau tangle density, Oligo_Factor_1
AC, PHFtau tangle density, Oligo_Factor_4
AC, Clinical diagnosis: AD vs NCI, Oligo_Factor_3
AC, Clinical diagnosis: AD vs NCI, Oligo_Factor_8
-log10(adj. pval)*sign(effect)
3
2
1
0
−1
−2
−3
Effect size
2
1
0.5

Supplementary Fig. 2

Gene loadings onto factors

Dotplot showing the fraction of nuclei and mean gene expression in each cluster of the top 2 genes (by loading) on each scHPF-derived factor (see Methods).

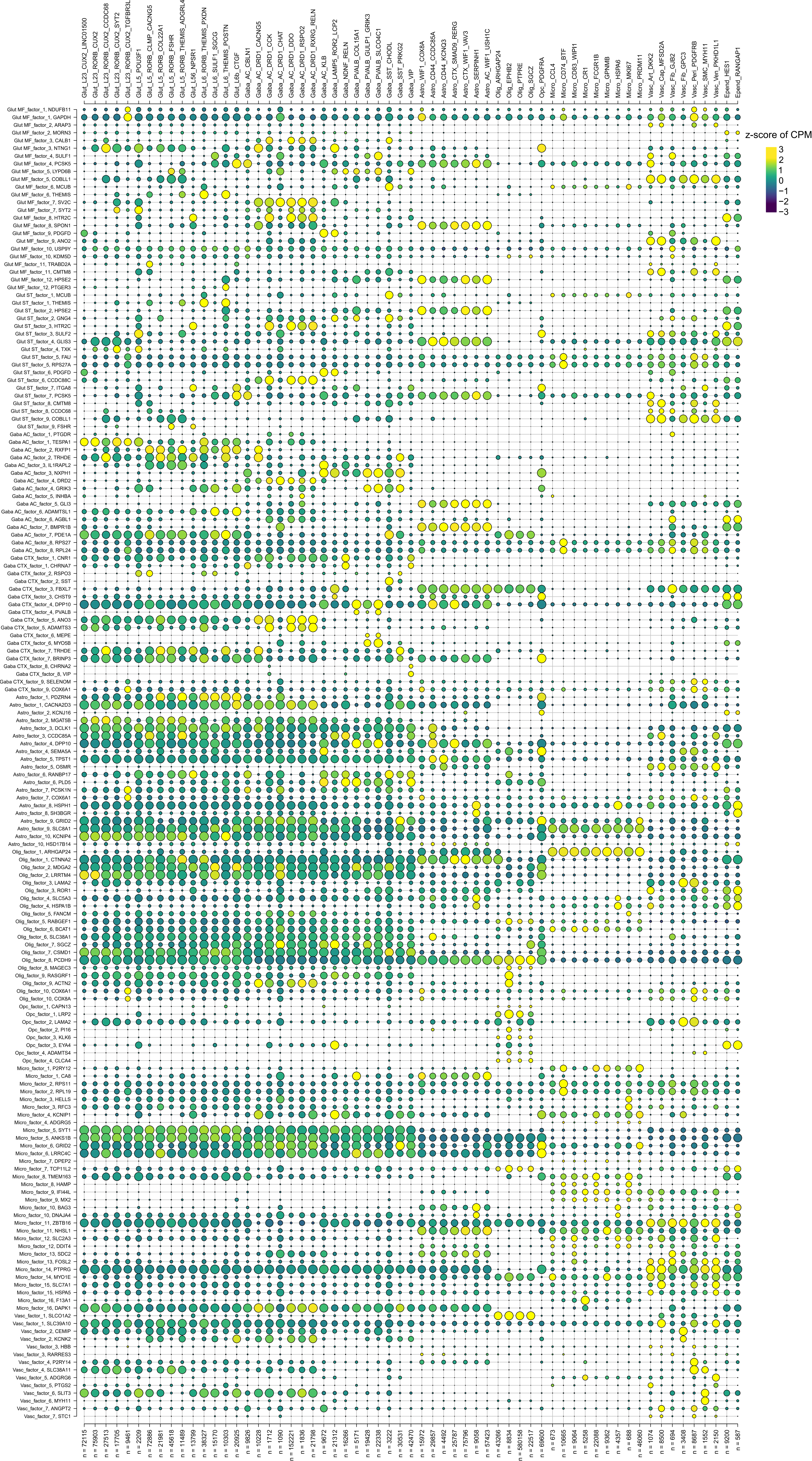

z-score of CPM
3
2
1
0
−1
−2
−3
Glut_L23_CUX2_LINC01500
Glut_L23_RORB_CUX2
Glut_L23_RORB_CUX2_CCDC68
Glut_L23_RORB_CUX2_SYT2
Glut_L23_RORB_CUX2_TGFBR3L
Glut_L5_POU3F1
Glut_L5_RORB_CLMP_CACNG5
Glut_L5_RORB_COL22A1
Glut_L5_RORB_FSHR
Glut_L5_RORB_THEMIS_ADGRL4
Glut_L56_NPSR1
Glut_L6_RORB_THEMIS_PXDN
Glut_L6_SULF1_SGCG
Glut_L6_THEMIS_POSTN
Glut_L6b_CTGF
Gaba_AC_CBLN1
Gaba_AC_DRD1_CACNG5
Gaba_AC_DRD1_CCK
Gaba_AC_DRD1_CHAT
Gaba_AC_DRD1_DDO
Gaba_AC_DRD1_RSPO2
Gaba_AC_DRD1_RXRG_RELN
Gaba_AC_KLB
Gaba_LAMP5_ROR2_LCP2
Gaba_NDNF_RELN
Gaba_PVALB_COL15A1
Gaba_PVALB_GULP1_GRIK3
Gaba_PVALB_SLCO4C1
Gaba_SST_CHODL
Gaba_SST_PRKG2
Gaba_VIP
Astro_WIF1_COX8A
Astro_CD44_CCDC85A
Astro_CD44_KCNQ3
Astro_CTX_SMAD9_RERG
Astro_CTX_WIF1_VAV3
Astro_SERPINH1
Astro_AC_WIF1_USH1C
Olig_ARHGAP24
Olig_EPHB2
Olig_PTPRE
Olig_SGCZ
Opc_PDGFRA
Micro_CCL4
Micro_CD74_BTF
Micro_CD83_WIPI1
Micro_CR1
Micro_FCGR1B
Micro_GPNMB
Micro_HSPA6
Micro_MKI67
Micro_PRDM11
Vasc_Art_DKK2
Vasc_Cap_MFSD2A
Vasc_Fib_GJB2
Vasc_Fib_GPC3
Vasc_Peri_PDGFRB
Vasc_SMC_MYH11
Vasc_Ven_PKHD1L1
Epend_HES1
Epend_RANGAP1
Glut MF_factor_1, NDUFB11
Glut MF_factor_1, GAPDH
Glut MF_factor_2, ARAP3
Glut MF_factor_2, MORN3
Glut MF_factor_3, CALB1
Glut MF_factor_3, NTNG1
Glut MF_factor_4, SULF1
Glut MF_factor_4, PCSK5
Glut MF_factor_5, LYPD6B
Glut MF_factor_5, COBLL1
Glut MF_factor_6, MCUB
Glut MF_factor_6, THEMIS
Glut MF_factor_7, SV2C
Glut MF_factor_7, SYT2
Glut MF_factor_8, HTR2C
Glut MF_factor_8, SPON1
Glut MF_factor_9, PDGFD
Glut MF_factor_9, ANO2
Glut MF_factor_10, USP9Y
Glut MF_factor_10, KDM5D
Glut MF_factor_11, TRABD2A
Glut MF_factor_11, CMTM8
Glut MF_factor_12, HPSE2
Glut MF_factor_12, PTGER3
Glut ST_factor_1, MCUB
Glut ST_factor_1, THEMIS
Glut ST_factor_2, HPSE2
Glut ST_factor_2, GNG4
Glut ST_factor_3, HTR2C
Glut ST_factor_3, SULF2
Glut ST_factor_4, GLIS3
Glut ST_factor_4, TXK
Glut ST_factor_5, FAU
Glut ST_factor_5, RPS27A
Glut ST_factor_6, PDGFD
Glut ST_factor_6, CCDC88C
Glut ST_factor_7, ITGA8
Glut ST_factor_7, PCSK5
Glut ST_factor_8, CMTM8
Glut ST_factor_8, CCDC68
Glut ST_factor_9, COBLL1
Glut ST_factor_9, FSHR
Gaba AC_factor_1, PTGDR
Gaba AC_factor_1, TESPA1
Gaba AC_factor_2, RXFP1
Gaba AC_factor_2, TRHDE
Gaba AC_factor_3, IL1RAPL2
Gaba AC_factor_3, NXPH1
Gaba AC_factor_4, DRD2
Gaba AC_factor_4, GRIK3
Gaba AC_factor_5, INHBA
Gaba AC_factor_5, GLI3
Gaba AC_factor_6, ADAMTSL1
Gaba AC_factor_6, AGBL1
Gaba AC_factor_7, BMPR1B
Gaba AC_factor_7, PDE1A
Gaba AC_factor_8, RPS27
Gaba AC_factor_8, RPL24
Gaba CTX_factor_1, CNR1
Gaba CTX_factor_1, CHRNA7
Gaba CTX_factor_2, RSPO3
Gaba CTX_factor_2, SST
Gaba CTX_factor_3, FBXL7
Gaba CTX_factor_3, CHST9
Gaba CTX_factor_4, DPP10
Gaba CTX_factor_4, PVALB
Gaba CTX_factor_5, ANO3
Gaba CTX_factor_5, ADAMTS3
Gaba CTX_factor_6, MEPE
Gaba CTX_factor_6, MYO5B
Gaba CTX_factor_7, TRHDE
Gaba CTX_factor_7, BRINP3
Gaba CTX_factor_8, CHRNA2
Gaba CTX_factor_8, VIP
Gaba CTX_factor_9, SELENOM
Gaba CTX_factor_9, COX6A1
Astro_factor_1, PDZRN4
Astro_factor_1, CACNA2D3
Astro_factor_2, KCNJ16
Astro_factor_2, MGAT5B
Astro_factor_3, DCLK1
Astro_factor_3, CCDC85A
Astro_factor_4, DPP10
Astro_factor_4, SEMA5A
Astro_factor_5, TPST1
Astro_factor_5, OSMR
Astro_factor_6, RANBP17
Astro_factor_6, PLD5
Astro_factor_7, PCSK1N
Astro_factor_7, COX6A1
Astro_factor_8, HSPH1
Astro_factor_8, SH3BGR
Astro_factor_9, GRID2
Astro_factor_9, SLC8A1
Astro_factor_10, KCNIP4
Astro_factor_10, HSD17B14
Olig_factor_1, ARHGAP24
Olig_factor_1, CTNNA2
Olig_factor_2, MDGA2
Olig_factor_2, LRRTM4
Olig_factor_3, LAMA2
Olig_factor_3, ROR1
Olig_factor_4, SLC5A3
Olig_factor_4, HSPA1B
Olig_factor_5, FANCM
Olig_factor_5, RABGEF1
Olig_factor_6, BCAT1
Olig_factor_6, SLC38A1
Olig_factor_7, SGCZ
Olig_factor_7, CSMD1
Olig_factor_8, PCDH9
Olig_factor_8, MAGEC3
Olig_factor_9, RASGRF1
Olig_factor_9, ACTN2
Olig_factor_10, COX6A1
Olig_factor_10, COX8A
Opc_factor_1, CAPN13
Opc_factor_1, LRP2
Opc_factor_2, LAMA2
Opc_factor_2, PI16
Opc_factor_3, KLK6
Opc_factor_3, EYA4
Opc_factor_4, ADAMTS4
Opc_factor_4, CLCA4
Micro_factor_1, P2RY12
Micro_factor_1, CA8
Micro_factor_2, RPS11
Micro_factor_2, RPL19
Micro_factor_3, HELLS
Micro_factor_3, RFC3
Micro_factor_4, KCNIP1
Micro_factor_4, ADGRG5
Micro_factor_5, SYT1
Micro_factor_5, ANKS1B
Micro_factor_6, GRID2
Micro_factor_6, LRRC4C
Micro_factor_7, DPEP2
Micro_factor_7, TCP11L2
Micro_factor_8, TMEM163
Micro_factor_8, HAMP
Micro_factor_9, IFI44L
Micro_factor_9, MX2
Micro_factor_10, BAG3
Micro_factor_10, DNAJA4
Micro_factor_11, ZBTB16
Micro_factor_11, NHSL1
Micro_factor_12, SLC2A3
Micro_factor_12, DDIT4
Micro_factor_13, FOSL2
Micro_factor_13, SDC2
Micro_factor_14, PTPRG
Micro_factor_14, MYO1E
Micro_factor_15, SLC7A1
Micro_factor_15, HSPA5
Micro_factor_16, F13A1
Micro_factor_16, DAPK1
Vasc_factor_1, SLCO1A2
Vasc_factor_1, SLC39A10
Vasc_factor_2, CEMIP
Vasc_factor_2, KCNK2
Vasc_factor_3, HBB
Vasc_factor_3, RARRES3
Vasc_factor_4, P2RY14
Vasc_factor_4, SLC38A11
Vasc_factor_5, ADGRG6
Vasc_factor_5, PTGS2
Vasc_factor_6, SLIT3
Vasc_factor_6, MYH11
Vasc_factor_7, ANGPT2
Vasc_factor_7, STC1
n = 72115
n = 75903
n = 27513
n = 17705
n = 9461
n = 2209
n = 72886
n = 21981
n = 45618
n = 11489
n = 13799
n = 38327
n = 15170
n = 10303
n = 20925
n = 9826
n = 10228
n = 1712
n = 1090
n = 152221
n = 1836
n = 21798
n = 9672
n = 21312
n = 16266
n = 5171
n = 19428
n = 22338
n = 3222
n = 30531
n = 42470
n = 15972
n = 29957
n = 4492
n = 25787
n = 75796
n = 9058
n = 57423
n = 43266
n = 8834
n = 580158
n = 22517
n = 69600
n = 673
n = 10665
n = 9064
n = 5258
n = 22088
n = 9362
n = 4357
n = 688
n = 46060
n = 1074
n = 8500
n = 694
n = 3408
n = 8687
n = 1552
n = 2150
n = 9200
n = 587

Supplementary Fig. 3

WGCNA module gene overview and associations with pathological and clinical phenotypes Heatmaps represent the Gene Set Enrichment Analysis p-values of genes in each WGCNA module compared to the ranked gene lists derived from differential genes (for clusters) or gene loadings (for factors). The bottom right panel shows a summary of p-values and effect sizes for module eigengenes with statistically significant (meta-analysis FDR-adjusted p-value <0.1, consistent directionality, meta-analysis I^2<50%, and p-value more significant than that of module eigengene 0 for all cell classes) and consistent associations with pathological and cognitive phenotypes across all three population groups.

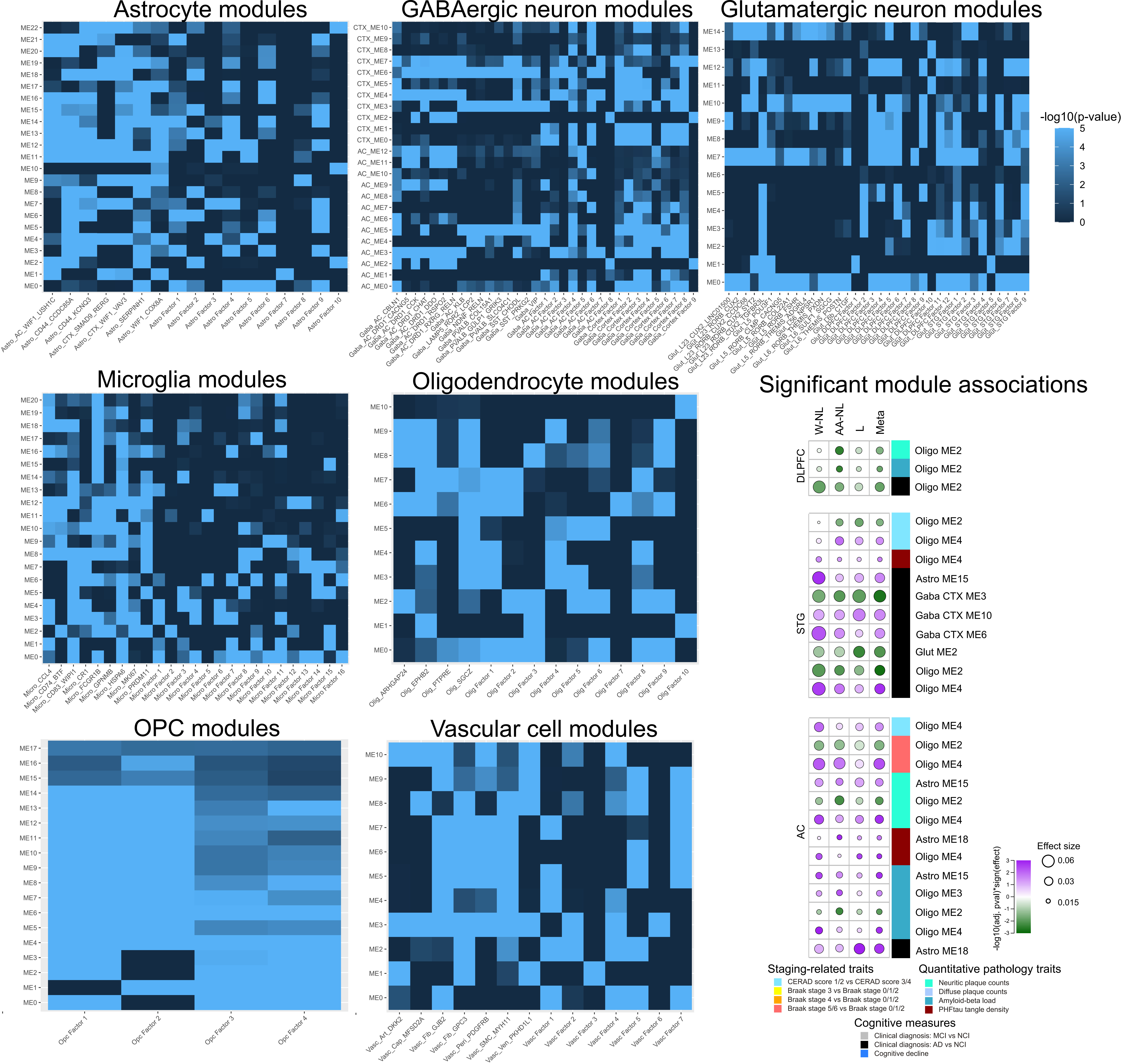
Astrocyte modules
GABAergic neuron modules
Glutamatergic neuron modules
-log10(p-value)
5
4
3
2
1
0
Astro_AC_WIF1_USH1C
Astro_CD44_CCDC85A
Astro_CD44_KCNQ3
Astro_CTX_SMAD9_RERG
Astro_CTX_WIF1_VAV3
Astro_SERPINH1
Astro_WIF1_COX8A
Astro Factor 1
Astro Factor 2
Astro Factor 3
Astro Factor 4
Astro Factor 5
Astro Factor 6
Astro Factor 7
Astro Factor 8
Astro Factor 9
Astro Factor 10
Microglia modules
Micro_CCL4
Micro_CD74_BTF
Micro_CD83_WIPI1
Micro_CR1
Micro_FCGR1B
Micro_GPNMB
Micro_HSPA6
Micro_MKI67
Micro_PRDM11
Oligodendrocyte modules
Olig_ARHGAP24
Olig_EPHB2
Olig_PTPRE
Olig_SGCZ
OPC modules
Opc Factor 1
Opc Factor 2
Opc Factor 3
Opc Factor 4
Vascular cell modules
Vasc_Art_DKK2
Vasc_Cap_MFSD2A
Vasc_Fib_GJB2
Vasc_Fib_GPC3
Vasc_Peri_PDGFRB
Vasc_SMC_MYH11
Vasc_Ven_PKHD1L1
Significant module associations
W-NL
AA-NL
L
Meta
DLPFC
Oligo ME2
Oligo ME2
Oligo ME2
STG
Oligo ME2
Oligo ME4
Oligo ME4
Astro ME15
Gaba CTX ME3
Gaba CTX ME10
Gaba CTX ME6
Glut ME2
Oligo ME2
Oligo ME4
AC
Oligo ME4
Oligo ME2
Oligo ME4
Astro ME15
Oligo ME2
Oligo ME4
Astro ME18
Oligo ME4
Astro ME15
Oligo ME3
Oligo ME2
Oligo ME4
Astro ME18
Effect size
0.06
0.03
0.015
-log10(adj. pval)*sign(effect)
Staging-related traits
CERAD score 1/2 vs CERAD score 3/4
Braak stage 3 vs Braak stage 0/1/2
Braak stage 4 vs Braak stage 0/1/2
Braak stage 5/6 vs Braak stage 0/1/2
Quantitative pathology traits
Neuritic plaque counts
Diffuse plaque counts
Amyloid-beta load
PHFtau tangle density
Cognitive measures
Clinical diagnosis: MCI vs NCI
Clinical diagnosis: AD vs NCI
Cognitive decline

Supplementary Fig. 4
Regional variation in cluster and factor associations
Visualization comparing region-specific p-values and effect size ratios for ANCOM-BC cluster associations (top row), the quasibinomial regression model cluster associations (second row), LINDA cluster associations (third row), sccomp cluster associations (fourth row), and linear model for factor associations (bottom row). The first column compares STG (x-axis) and DLPFC (y-axis) p-values. The middle column compares STG (x-axis) and AC (y-axis) p-values. The third column compares DLPFC (x-axis) and AC (y-axis). The size of each dot represents the absolute value of the log2-transformed ratio between effect sizes in the two regions being compared. Colors indicate whether a specific cell type/factor association with a phenotype was significant in both regions (black), only in one region (red or blue), or in neither region (grey).

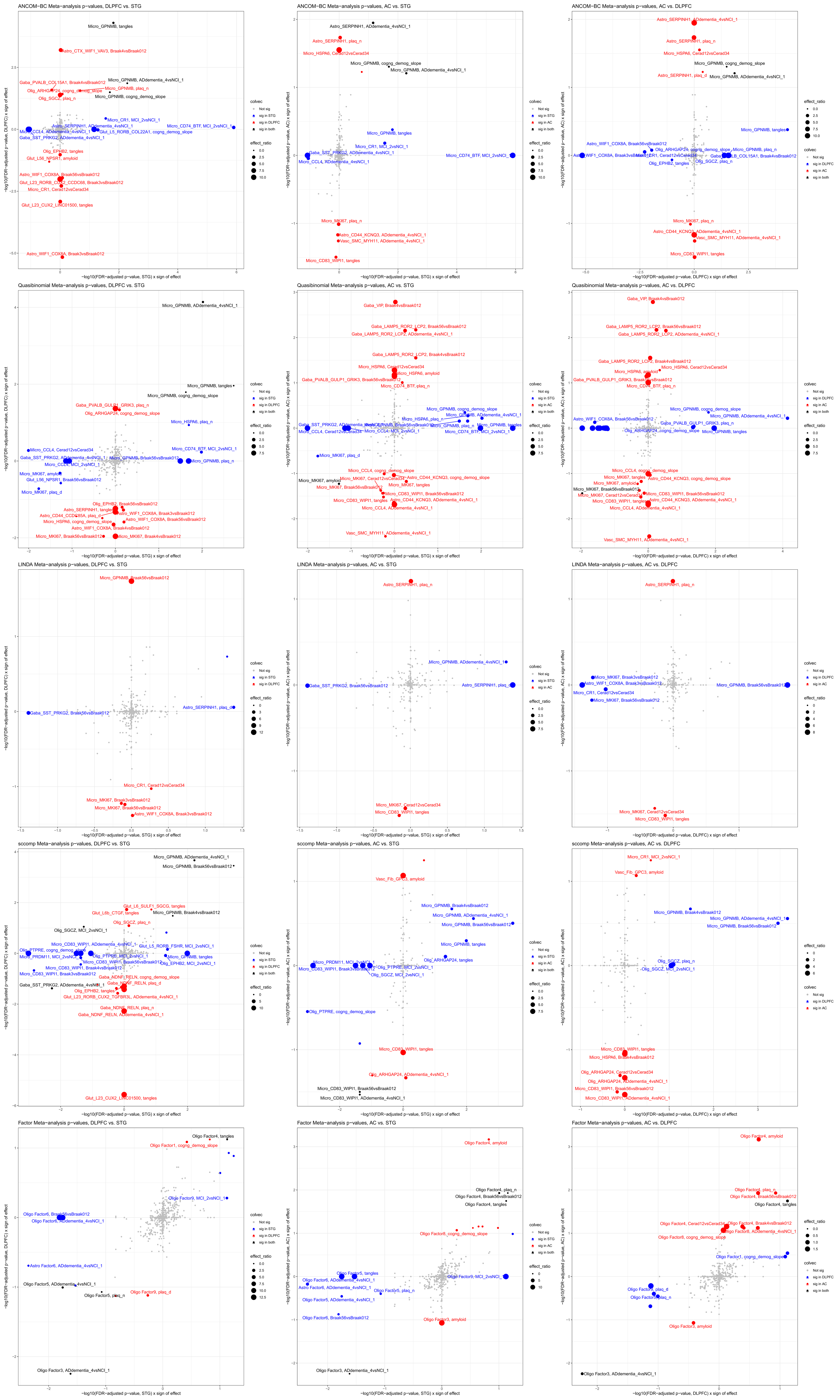
ANCOM−BC Meta−analysis p−values, DLPFC vs. STG
Micro_GPNMB, tangles
Astro_CTX_WIF1_VAV3, Braak4vsBraak012
Gaba_PVALB_COL15A1, Braak4vsBraak012
Micro_GPNMB, ADdementia_4vsNCI_1
Olig_ARHGAP24, cogng_demog_slope
Micro_GPNMB, plaq_n
Olig_SGCZ, plaq_n
Micro_GPNMB, cogng_demog_slope
Micro_CR1, MCI_2vsNCI_1
Astro_SERPINH1, ADdementia_4vsNCI_1
Micro_CCL4, ADdementia_4vsNCI_1
Glut_L5_RORB_COL22A1, cogng_demog_slope
Micro_CD74_BTF, MCI_2vsNCI_1
Gaba_SST_PRKG2, ADdementia_4vsNCI_1
Olig_EPHB2, tangles
Glut_L56_NPSR1, amyloid
Astro_WIF1_COX8A, Braak56vsBraak012
Glut_L23_RORB_CCDC68, Braak3vsBraak012
Micro_CR1, Cerad12vsCerad34
Glut_L23_CUX2_LINC01500, tangles
Astro_WIF1_COX8A, Braak3vsBraak012
−log10(FDR−adjusted p−value, STG) x sign of effect
−log10(FDR−adjusted p−value, DLPFC) x sign of effect
colvec
Not sig
sig in STG
sig in DLPFC
sig in both
effect_ratio
ANCOM−BC Meta−analysis p−values, AC vs. STG
Astro_SERPINH1, ADdementia_4vsNCI_1
Astro_SERPINH1, plaq_n
Micro_HSPA6, Cerad12vsCerad34
Micro_GPNMB, cogng_demog_slope
Micro_GPNMB, ADdementia_4vsNCI_1
Micro_GPNMB, tangles
Micro_CR1, MCI_2vsNCI_1
Gaba_SST_PRKG2, ADdementia_4vsNCI_1
Micro_CD74_BTF, MCI_2vsNCI_1
Micro_CCL4, ADdementia_4vsNCI_1
Micro_MKI67, plaq_n
Astro_CD44_KCNQ3, ADdementia_4vsNCI_1
Vasc_SMC_MYH11, ADdementia_4vsNCI_1
Micro_CD83_WIPI1, tangles
−log10(FDR−adjusted p−value, STG) x sign of effect
−log10(FDR−adjusted p−value, AC) x sign of effect
sig in AC
ANCOM−BC Meta−analysis p−values, AC vs. DLPFC
Astro_SERPINH1, ADdementia_4vsNCI_1
Astro_SERPINH1, plaq_n
Micro_HSPA6, Cerad12vsCerad34
Micro_GPNMB, cogng_demog_slope
Astro_SERPINH1, plaq_d
Micro_GPNMB, ADdementia_4vsNCI_1
Micro_GPNMB, tangles
Astro_WIF1_COX8A, Braak56vsBraak012
Olig_ARHGAP24, cogng_demog_slope
Micro_GPNMB, plaq_n
Astro_WIF1_COX8A, Braak3vsBraak012
Micro_CR1, Cerad12vsCerad34
Gaba_PVALB_COL15A1, Braak4vsBraak012
Olig_EPHB2, tangles
Olig_SGCZ, plaq_n
Micro_MKI67, plaq_n
Astro_CD44_KCNQ3, ADdementia_4vsNCI_1
Vasc_SMC_MYH11, ADdementia_4vsNCI_1
Micro_CD83_WIPI1, tangles
−log10(FDR−adjusted p−value, DLPFC) x sign of effect
Quasibinomial Meta−analysis p−values, DLPFC vs. STG
Micro_GPNMB, ADdementia_4vsNCI_1
Micro_GPNMB, tangles
Micro_GPNMB, cogng_demog_slope
Gaba_PVALB_GULP1_GRIK3, plaq_n
Olig_ARHGAP24, cogng_demog_slope
Micro_HSPA6, plaq_n
Micro_CCL4, Cerad12vsCerad34
Micro_CD74_BTF, MCI_2vsNCI_1
Gaba_SST_PRKG2, ADdementia_4vsNCI_1
Micro_GPNMB, Braak56vsBraak012
Micro_CCL4, MCI_2vsNCI_1
Micro_GPNMB, plaq_n
Micro_MKI67, amyloid
Glut_L56_NPSR1, Braak56vsBraak012
Micro_MKI67, plaq_d
Olig_EPHB2, Braak56vsBraak012
Astro_SERPINH1, tangles
Astro_WIF1_COX8A, Braak3vsBraak012
Astro_CD44_CCDC85A, plaq_d
Astro_WIF1_COX8A, Braak56vsBraak012
Micro_HSPA6, cogng_demog_slope
Astro_WIF1_COX8A, Braak4vsBraak012
Micro_MKI67, Braak56vsBraak012
Micro_MKI67, Braak4vsBraak012
Quasibinomial Meta−analysis p−values, AC vs. STG
Gaba_VIP, Braak4vsBraak012
Gaba_LAMP5_ROR2_LCP2, Braak56vsBraak012
Gaba_LAMP5_ROR2_LCP2, ADdementia_4vsNCI_1
Gaba_LAMP5_ROR2_LCP2, Braak4vsBraak012
Micro_HSPA6, Cerad12vsCerad34
Micro_HSPA6, amyloid
Gaba_PVALB_GULP1_GRIK3, Braak56vsBraak012
Micro_CD74_BTF, plaq_n
Micro_GPNMB, cogng_demog_slope
Micro_GPNMB, ADdementia_4vsNCI_1
Gaba_SST_PRKG2, ADdementia_4vsNCI_1
Micro_GPNMB, Braak56vsBraak012
Micro_HSPA6, plaq_n
Micro_GPNMB, plaq_n
Micro_GPNMB, tangles
Micro_CCL4, Cerad12vsCerad34
Micro_CCL4, MCI_2vsNCI_1
Micro_CD74_BTF, MCI_2vsNCI_1
Micro_MKI67, plaq_d
Micro_CCL4, cogng_demog_slope
Micro_MKI67, amyloid
Micro_MKI67, Cerad12vsCerad34
Astro_CD44_KCNQ3, cogng_demog_slope
Micro_MKI67, Braak56vsBraak012
Micro_MKI67, tangles
Micro_CD83_WIPI1, Braak56vsBraak012
Micro_CD83_WIPI1, tangles
Astro_CD44_KCNQ3, ADdementia_4vsNCI_1
Micro_CCL4, ADdementia_4vsNCI_1
Vasc_SMC_MYH11, ADdementia_4vsNCI_1
Quasibinomial Meta−analysis p−values, AC vs. DLPFC
Gaba_VIP, Braak4vsBraak012
Gaba_LAMP5_ROR2_LCP2, Braak56vsBraak012
Gaba_LAMP5_ROR2_LCP2, ADdementia_4vsNCI_1
Gaba_LAMP5_ROR2_LCP2, Braak4vsBraak012
Micro_HSPA6, Cerad12vsCerad34
Micro_HSPA6, amyloid
Gaba_PVALB_GULP1_GRIK3, Braak56vsBraak012
Micro_CD74_BTF, plaq_n
Micro_GPNMB, cogng_demog_slope
Micro_GPNMB, ADdementia_4vsNCI_1
Astro_WIF1_COX8A, Braak56vsBraak012
Gaba_PVALB_GULP1_GRIK3, plaq_n
Olig_ARHGAP24, cogng_demog_slope
Micro_GPNMB, tangles
Micro_CCL4, cogng_demog_slope
Micro_MKI67, tangles
Astro_CD44_KCNQ3, cogng_demog_slope
Micro_MKI67, amyloid
Micro_MKI67, Braak56vsBraak012
Micro_MKI67, Cerad12vsCerad34
Micro_CD83_WIPI1, Braak56vsBraak012
Micro_CD83_WIPI1, tangles
Astro_CD44_KCNQ3, ADdementia_4vsNCI_1
Micro_CCL4, ADdementia_4vsNCI_1
Vasc_SMC_MYH11, ADdementia_4vsNCI_1
LINDA Meta−analysis p−values, DLPFC vs. STG
Micro_GPNMB, Braak56vsBraak012
Gaba_SST_PRKG2, Braak56vsBraak012
Astro_SERPINH1, plaq_d
Micro_CR1, Cerad12vsCerad34
Micro_MKI67, Braak3vsBraak012
Micro_MKI67, Braak56vsBraak012
Astro_WIF1_COX8A, Braak3vsBraak012
LINDA Meta−analysis p−values, AC vs. STG
Astro_SERPINH1, plaq_n
Micro_GPNMB, ADdementia_4vsNCI_1
Gaba_SST_PRKG2, Braak56vsBraak012
Astro_SERPINH1, plaq_d
Micro_MKI67, Cerad12vsCerad34
Micro_CD83_WIPI1, tangles
LINDA Meta−analysis p−values, AC vs. DLPFC
Astro_SERPINH1, plaq_n
Micro_MKI67, Braak3vsBraak012
Astro_WIF1_COX8A, Braak3vsBraak012
Micro_CR1, Cerad12vsCerad34
Micro_MKI67, Braak56vsBraak012
Micro_GPNMB, Braak56vsBraak012
Micro_MKI67, Cerad12vsCerad34
Micro_CD83_WIPI1, tangles
sccomp Meta−analysis p−values, DLPFC vs. STG
Micro_GPNMB, ADdementia_4vsNCI_1
Micro_GPNMB, Braak56vsBraak012
Glut_L6_SULF1_SGCG, tangles
Glut_L6b_CTGF, tangles
Micro_GPNMB, Braak4vsBraak012
Olig_SGCZ, plaq_n
Olig_SGCZ, MCI_2vsNCI_1
Micro_CD83_WIPI1, ADdementia_4vsNCI_1
Glut_L5_RORB_FSHR, MCI_2vsNCI_1
Olig_PTPRE, cogng_demog_slope
Micro_PRDM11, MCI_2vsNCI_1
Olig_PTPRE, MCI_2vsNCI_1
Micro_GPNMB, tangles
Micro_CD83_WIPI1, Braak56vsBraak012
Olig_EPHB2, MCI_2vsNCI_1
Micro_CD83_WIPI1, Braak4vsBraak012
Micro_CD83_WIPI1, Braak3vsBraak012
Gaba_NDNF_RELN, cogng_demog_slope
Gaba_SST_PRKG2, ADdementia_4vsNCI_1
Gaba_NDNF_RELN, plaq_d
Olig_EPHB2, tangles
Glut_L23_RORB_CUX2_TGFBR3L, ADdementia_4vsNCI_1
Gaba_NDNF_RELN, plaq_n
Gaba_NDNF_RELN, ADdementia_4vsNCI_1
Glut_L23_CUX2_LINC01500, tangles
sccomp Meta−analysis p−values, AC vs. STG
Vasc_Fib_GPC3, amyloid
Micro_GPNMB, Braak4vsBraak012
Micro_GPNMB, ADdementia_4vsNCI_1
Micro_GPNMB, Braak56vsBraak012
Micro_GPNMB, tangles
Olig_ARHGAP24, tangles
Micro_PRDM11, MCI_2vsNCI_1
Micro_CD83_WIPI1, Braak3vsBraak012
Olig_PTPRE, MCI_2vsNCI_1
Olig_SGCZ, MCI_2vsNCI_1
Olig_PTPRE, cogng_demog_slope
Micro_CD83_WIPI1, tangles
Olig_ARHGAP24, ADdementia_4vsNCI_1
Micro_CD83_WIPI1, Braak56vsBraak012
Micro_CD83_WIPI1, ADdementia_4vsNCI_1
sccomp Meta−analysis p−values, AC vs. DLPFC
Micro_CR1, MCI_2vsNCI_1
Vasc_Fib_GPC3, amyloid
Micro_GPNMB, Braak4vsBraak012
Micro_GPNMB, ADdementia_4vsNCI_1
Micro_GPNMB, Braak56vsBraak012
Olig_SGCZ, plaq_n
Olig_SGCZ, MCI_2vsNCI_1
Micro_CD83_WIPI1, tangles
Micro_HSPA6, Braak4vsBraak012
Olig_ARHGAP24, Cerad12vsCerad34
Olig_ARHGAP24, ADdementia_4vsNCI_1
Micro_CD83_WIPI1, Braak56vsBraak012
Micro_CD83_WIPI1, ADdementia_4vsNCI_1
Factor Meta−analysis p−values, DLPFC vs. STG
Oligo Factor4, tangles
Oligo Factor1, cogng_demog_slope
Oligo Factor9, MCI_2vsNCI_1
Oligo Factor6, Braak56vsBraak012
Oligo Factor6, ADdementia_4vsNCI_1
Astro Factor6, ADdementia_4vsNCI_1
Oligo Factor5, ADdementia_4vsNCI_1
Oligo Factor5, plaq_n
Oligo Factor9, plaq_d
Oligo Factor3, ADdementia_4vsNCI_1
Factor Meta−analysis p−values, AC vs. STG
Oligo Factor4, amyloid
Oligo Factor4, plaq_n
Oligo Factor4, Braak56vsBraak012
Oligo Factor4, tangles
Oligo Factor8, cogng_demog_slope
Oligo Factor5, tangles
Oligo Factor6, ADdementia_4vsNCI_1
Astro Factor6, ADdementia_4vsNCI_1
Oligo Factor9, MCI_2vsNCI_1
Oligo Factor5, plaq_n
Oligo Factor5, ADdementia_4vsNCI_1
Oligo Factor6, Braak56vsBraak012
Oligo Factor3, amyloid
Oligo Factor3, ADdementia_4vsNCI_1
Factor Meta−analysis p−values, AC vs. DLPFC
Oligo Factor4, amyloid
Oligo Factor4, plaq_n
Oligo Factor4, Braak56vsBraak012
Oligo Factor4, tangles
Oligo Factor4, Cerad12vsCerad34
Oligo Factor4, Braak4vsBraak012
Oligo Factor8, ADdementia_4vsNCI_1
Oligo Factor8, cogng_demog_slope
Oligo Factor1, cogng_demog_slope
Oligo Factor5, plaq_d
Oligo Factor5, plaq_n
Oligo Factor3, amyloid
Oligo Factor3, ADdementia_4vsNCI_1

Supplementary Fig. 5

Xenium mapped cluster proportions and tissue-level visualization

a) Relative proportions of clusters per donor (each dot represents one donor) in the DLPFC derivefrom snRNA-seq (y-axes) and after label transfer onto Xenium data (x-axis). Although there is some variability in absolute proportions between the two methods (Astro_CTX_WIF1_VAV3 and Micro_HSPA6, for example), there is an overall association between the relative abundance of clusters. b) Tissue-level visualization of cell clusters from Xenium multiplexed ISH data on 33 DLPFC tissue sections. Leftmost column maps the location of glutamatergic clusters (with labels transferred from snRNA-seq reference data) within each tissue section. Columns 2-4 show the location of all microglia nuclei (red+grey), nuclei belonging to Micro_GPNMB cluster (red), and non-Micro_GPNMB microglial nuclei (grey) respectively in each tissue section. Column 5 shows the Micro_GPNMB cluster proportion versus negative slope of cognitive decline for all individuals (top row) and per individual from whom the tissue section was obtained (other rows). Sections are ordered top to bottom by individuls with the most severe to least severe cognitive decline. Columns 6-9 are the same as columns 2-4, but for oligodendrocytes and the Olig_ARHGAP24 cluster. Columns 10-13 are the same as columns 6-9, but for the Olig_EPHB2 cluster, and PHFtau tangle density as the phenotype of interest. Note that the order of sections in columns 10-13 is different, as they are arranged by lowest to highest PHFtau tangle density.

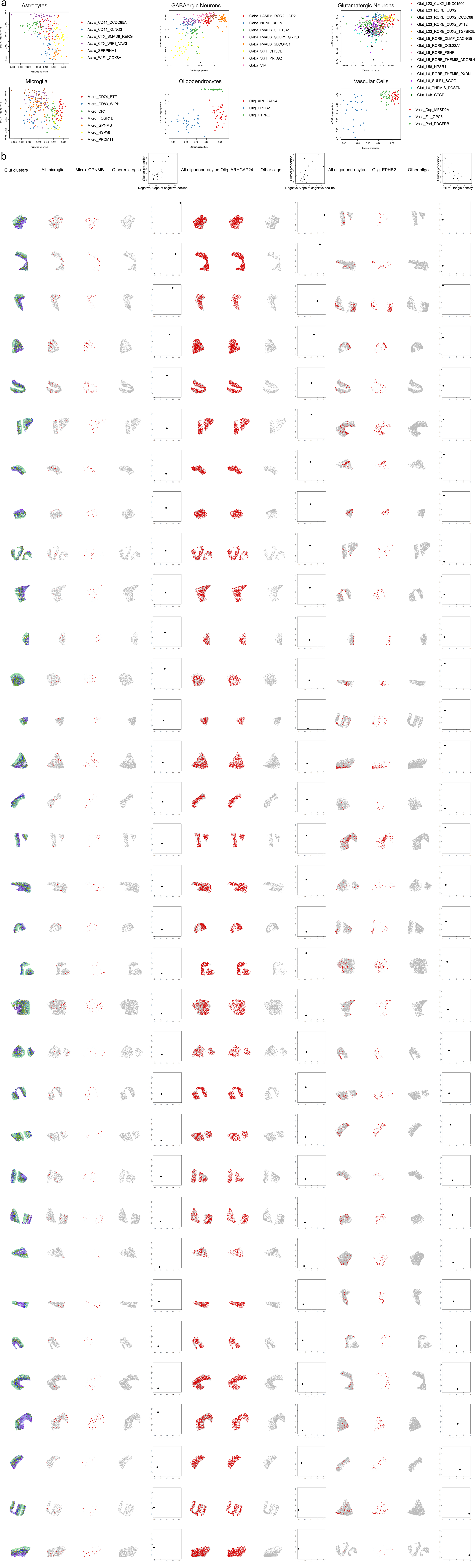

a
Astrocytes
Astro_CD44_CCDC85A
Astro_CD44_KCNQ3
Astro_CTX_SMAD9_RERG
Astro_CTX_WIF1_VAV3
Astro_SERPINH1
Astro_WIF1_COX8A
GABAergic Neurons
Gaba_LAMP5_ROR2_LCP2
Gaba_NDNF_RELN
Gaba_PVALB_COL15A1
Gaba_PVALB_GULP1_GRIK3
Gaba_PVALB_SLCO4C1
Gaba_SST_CHODL
Gaba_SST_PRKG2
Gaba_VIP
Glutamatergic Neurons
Glut_L23_CUX2_LINC01500
Glut_L23_RORB_CUX2
Glut_L23_RORB_CUX2_CCDC68
Glut_L23_RORB_CUX2_SYT2
Glut_L23_RORB_CUX2_TGFBR3L
Glut_L5_RORB_CLMP_CACNG5
Glut_L5_RORB_COL22A1
Glut_L5_RORB_FSHR
Glut_L5_RORB_THEMIS_ADGRL4
Glut_L56_NPSR1
Glut_L6_RORB_THEMIS_PXDN
Glut_L6_SULF1_SGCG
Glut_L6_THEMIS_POSTN
Glut_L6b_CTGF
Microglia
Micro_CD74_BTF
Micro_CD83_WIPI1
Micro_CR1
Micro_FCGR1B
Micro_GPNMB
Micro_HSPA6
Micro_PRDM11
Oligodendrocytes
Olig_ARHGAP24
Olig_EPHB2
Olig_PTPRE
Vascular Cells
Vasc_Cap_MFSD2A
Vasc_Fib_GPC3
Vasc_Peri_PDGFRB
b
Glut clusters
All microglia
Micro_GPNMB
Other microglia
Cluster proportion
Negative Slope of cognitive decline
All oligodendrocytes
Olig_ARHGAP24
Other oligo
All oligodendrocytes
Olig_EPHB2
Other oligo
PHFtau tangle density
