# Supplementary material for: Cell-type signatures of Alzheimer’s disease shared across population groups
Source: Nature. 2026 Jul 15;657(8130):191–201. doi: 10.1038/s41586-026-10793-0 (PMC13538069; doi:10.1038/s41586-026-10793-0)
Supplement: Supplementary file 3 [file 41586_2026_10793_MOESM4_ESM.pdf]

nature portfolio

Peer Review File

Alzheimer’s Disease cell type signatures shared across population groups

Corresponding Author: Dr Vilas Menon

Any redactions in this file are there to maintain patient confidentiality, the confidentiality of unpublished data, or to remove third-party material.

This file contains all reviewer reports in order by version, followed by all author rebuttals in order by version.

Version 0:

Reviewer comments:

Referee #1

(Remarks to the Author)
In this study, Luquez et al. use joint single-nucleus RNA-seq and ATAC-seq to profile three brain regions from 167 post-mortem donors across three self-reported racial/ethnic groups (non-Latin White, African American, Latin), yielding 1.9 million nuclei across major neuronal and glial classes. The authors identify microglial, neuronal, and astrocyte signatures that display conserved associations with AD pathology and cognitive decline across all three groups. They also report continuous gene expression factors that show strong associations with disease phenotypes and found that cognitively impaired donors can be separated into six molecularly distinct groups. Overall, this is a carefully executed, high-quality dataset paper whose main strength is the diverse sampling and explicit cross-group analysis demonstrating that key AD-associated cell state signatures are shared across self-reported racial/ethnic groups.

The work is moderately novel in that many cell-states have been reported previously, but here are convincingly shown to be conserved across three self-reported racial/ethnic groups, and the resulting multi-ethnic, multi-region dataset will be a useful community resource. Data quality and scale are strong, the multiome and factor-based design is appropriate, and the analytical framework is methodologically sound and clearly presented, with ATAC-seq interpreted cautiously given depth limitations. I see no fundamental flaws in the analysis, and my comments focus on clarifying aspects of design and statistics and suggesting a few additions that would further strengthen the manuscript.

Major comments

1)
There are several numbers in the manuscript that are somewhat difficult to reconcile on a first reading (160 individuals mentioned in the introduction section; “we selected 164 participants” in the Methods section; 167 donors in Figure 1A). Please make sure it is clear for each analysis how many individuals the analysis is based on.

2)
The manuscript states that BH FDR correction was applied “across broad cell types, regions, and clinical and pathological traits”. I think it remains unclear what the precise correction universe was. It would be helpful to specify exactly how the FDR batches were defined. For example, whether BH correction was applied (i) within each broad cell class across all cluster times region times phenotype tests, (ii) across all tests in the entire dataset, or (iii) separately for each phenotype or region.

3)
A central strength of the work is the inclusion of African American and Latin donors alongside non-Latin White donors, and the demonstration that key cell-type signatures are conserved across these self-reported groups. The analysis across self-reported race and ethnicity categories is a valuable aspect of this study. If genetic data are available, a brief descriptive characterization of genetic ancestry would, in my view, further strengthen the manuscript. In particular, it would be informative to show how donors from each self-reported category are distributed across the underlying genetic ancestry variation (for example, via PCA or ancestry-proportion estimates), and, where sample size allows, whether the conserved transcriptomic associations are observed across donors spanning different ancestry backgrounds. Such an analysis would

add useful context on how the identified shared signatures generalize across both socially defined categories and underlying continuous genetic diversity, without requiring any changes to the core analysis.

4)
The identification of six donor subgroups based on STG composition and factor values is intriguing. Because subgroup discovery in relatively small cohorts can be sensitive to analytical choices, it would strengthen the interpretation to provide a bit more context on the robustness of these groupings. In particular, could the authors comment on the stability of the subgroup structure? For example, whether similar broad groupings are observed under reasonable alternative clustering settings or distance metrics? Even a short qualitative statement would help readers understand the extent to which these six groups should be viewed as preliminary candidate molecular subtypes, pending replication in larger cohorts.

5)
The observation that the KANSL1 locus shows lower chromatin accessibility in African American donors compared to non-Latin White donors is intriguing. If I did not overlook this, it would be helpful to clarify whether this difference is supported by a formal statistical test, and, if so, how multiple comparisons were handled.

Minor comments

1)
The sentence on lines 300–302 appears truncated (".... ARHGAP24+ oligodendrocyte proportions and more severe ....").

2)
There appears to be a minor typographical error on lines 373–376 ("re not distinguished"), which I believe should read "are not distinguished".

3)
For the donor subgrouping, a short justification for choosing STG specifically (as opposed to another region or combined regions) would be useful.

Referee #2

(Remarks to the Author)
This manuscript presents a large, integrated single-nucleus multi-omic study of Alzheimer's disease–related molecular programmes across multiple brain regions and with samples from different population groups. By combining transcriptomic and chromatin accessibility profiling with compositional and expression-based analyses, the authors aim to identify cell-type- and state-specific signatures associated with clinical and pathological phenotypes, and to assess the extent to which these associations are consistent across populations groups. The dataset is substantial and the overall direction is timely, with potential value both as a resource and as a framework for representative sampling in neurodegeneration.

Major strengths
• The study tackles a high-impact question regarding the generalisability of disease-associated cellular programmes across population groups, using a multi-region design and multi-omic profiling.
• Integration of transcriptomic and chromatin accessibility data strengthens cell-state definition and provides a route towards regulatory interpretation, even if mechanistic inference remains limited.
• Regardless of specific associations, the dataset and analytical framework are likely to be of broad value to the community as a resource for future studies.

Concerns
1. A central claim of the manuscript concerns the consistency ("conservation") of disease-associated signatures across population groups. Although the authors clearly define "conserved" in statistical terms (meta-analysis significance, consistent direction of effect, and limited heterogeneity), which improves transparency, the manuscript remains affected by an important interpretational issue. Specifically, the current analyses do not fully distinguish whether the observed consistency reflects: (i) true biological similarity across groups, (ii) limited statistical power to detect differences, or (iii) modelling and normalisation choices that may bias results toward similarity. Where possible, the manuscript would be strengthened by more direct tests of difference (e.g. sensitivity analyses, effect-size comparisons, or explicit interaction-style reasoning) that demonstrate meaningful similarity rather than simply non-heterogeneity. In parallel, the language used to describe "conservation" should be carefully reviewed and not used broadly to avoid over-generalisation.

2. The use of self-reported race and ethnicity is transparent and appropriate. However, because the donors largely share a broad geographic and societal context, the observed consistency of cellular signatures could plausibly reflect environmental convergence and shared life experience rather than ancestry-independent biology. As such, the study cannot establish that the reported signatures are conserved across populations in a global or genetic sense, particularly in the absence of geographically distinct cohorts or analyses stratified by genetic ancestry. Although this limitation is acknowledged, the framing should be more consistently maintained across the manuscript to prevent readers from inferring conclusions about genetic ancestry that are not directly supported by the data.

3. A major limitation of the study is that it is fundamentally observational, relying on associations between cellular states and clinical or pathological phenotypes. Although this is expected and acceptable for a study of this scope.

4. Many of the key conclusions rely on differential abundance or compositional analyses of cell subclusters. Such analyses in single-nucleus datasets are inherently sensitive to nucleus recovery, QC thresholds, batch effects, regional sampling imbalance, and the choice of compositional model. While the analytical framework is sound and clearly described, the manuscript does not sufficiently demonstrate the robustness of key abundance findings to reasonable alternative analytical choices. This would be strengthened by additional robustness or sensitivity analyses (e.g. alternative QC thresholds, stricter filtering, or complementary compositional modelling strategies) showing that the main conclusions are stable. But if this was done, then it needs to be clearly stated in the manuscript.

5. Pooling of samples prior to library preparation with subsequent genetic demultiplexing is a reasonable design choice. However, unavoidable misassignment, undetected same-donor doublets, or exclusion of ambiguous nuclei may reduce between-donor variance and bias results toward apparent similarity across groups. This is particularly relevant given the emphasis on cross-group consistency. The manuscript would be strengthened by more explicit reporting of donor assignment confidence and exclusion rates, as well as sensitivity analyses demonstrating that key cross-group conclusions persist under stricter singlet or assignment-confidence thresholds.

6. On a more general note, the manuscript will benefit from clarity in handling and interpretation of region-specific effects when not all individuals contribute all brain regions.

Referee #3

(Remarks to the Author)
Luquez, Algoo and colleagues report on a framework of analyses relevant for Alzheimer's Disease (AD) across racial and ethnic groups. Authors highlight the gap in the field regarding the underrepresentation of non-white individuals in AD studies. Their study aims to uncover 1) which cell type-specific signatures are found in AD across population groups, and 2) which subtypes of AD may exist across these populations group. Authors generated sn-RNAseq and ATAC-seq datasets of 399 samples of 160 individuals spanning 3 self-reported races and ethnicity (African-american, Latin White, non-Latin White) across 3 brain regions. Authors found 61 clusters across all major cell classes. To interpret these data, authors employed discrete and continuous covariates. Authors identified myeloid cell signatures that are well conserved across the three population groups. This manuscript also reports continuous factors that were identified in the dataset that later are relevant for dissection of molecular signatures. Transcriptomic findings were performed with the Xenium platform. Finally, the authors take a data-driven approach considering cluster and factor composition to determine what they framed as "molecular subgroups". These groups transcended pathological staging but segregated AD and MCI-predominant subgroups.
Overall, the manuscript aims to fill a research gap, and its findings are relevant for AD in cross-ancestry research. It is appreciated that results are available in the form of an online shiny app as a reference resource. Nonetheless, when reading the paper, several aspects about novelty, reasoning, and the rationale behind analytical decision-making as well as interpretation raised concern. Addressing these issues would significantly improve the manuscript . Below thespecific major comments are listed.

1. Authors employ self-reported race and ethnicity to account for effects of life experience, as they may act as either confounders or disease-driving factors in a polygenic and multifactorial disease such as sporadic AD. Considering multi-ancestry cohort studies such as GWAS or TWAS, where ancestry is genetically inferred with haplotypes, here, race/ethnicity is self-reported. The authors do not explain how self-reported race and ethnicity are different to genetic ancestry. They do explain that self-reported race is used to account for gene-environment interactions during life. How different is the self-reported race from the genetic ancestry for these individuals? Would that impact the whole analysis? This is not outlined in the introduction, methods, or discussion. This benchmarking is crucial, as linkage of the data to ancestry/race is the foundation of the paper. Authors for instance could reconstruct partial genotypes from sn-RNAseq and ATAC-seq data. Alternatively, some (or all) of the donors should be benchmarked by SNP genotyping. More compelling evidence is required to prove face-validity of race/ethnicity self reporting.
2. Nuclear suspensions were pooled and then demultiplexed. A supplement to explain the compositions of the suspensions is missing. Authors point to the data availability statement that contains a link to Synapse. However, it cannot be found there. How does this pooling strategy look? Were they balanced by population group or brain region (3 of each)? Nevertheless, this information should be included in the manuscript as a Supplementary Figure or Table and included with more detail in the Methods (Although there are some aspects in the Methods section, it is insufficient).
3. The Xenium validation figure is hard to understand. What is the added value of the gradient of the colored sections? From the visual inspection of the plots with the red dots, Micro_GPNMB does not seem to increase in individuals with more cognitive decline. Negative slope as a continuous variable in such plots makes the analysis convoluted and not intuitive. Authors need to present these data in a more accessible fashion to allow proper interpretation, instead of hard to interpret and integrate spatial plots.
4. In Figure 5, performing only hierarchical clustering of cell proportions and factors, obtained in the same data seems circular as a standalone analysis. Seems that the same data is "represented" in different fashions, which would not be an orthogonal validation. Orthogonal analyses, for instance with WGCNA or similar strategies, will strengthen these associations and support the claims by the authors.
5. Comprehensive rationale behind MCI and AD selection only for the definition of subgroups is missing. The motivation towards further dissection of pathological states is clear, but how do controls behave? Or how do they load these factors? It is anticipated that controls also contain a wealth of information on non-pathological data across ancestries.
6. Presenting the concept of "transcriptomically defined subgroups" is problematic. What is the biological or practical relevance of defining them? It is evident that the interpretation of these groups transcends other categories such as race or

pathological features. However, an individual suffering from AD will not be further sub-classified as Group 4, 5 or 6 unless they load heavily on Oligodendrocyte factor 1, Astro_CD44, or Astrocyte Factor 1, respectively. It needs to be clarified. The field of genomics has employed the term “endophenotypes” to propose further dissection of diseases to a higher resolution. Please note that this is an evolving field, and these definitions are always subject to revision. These considerations of further dissection of disease signatures should be addressed at the analysis, interpretation, and discussion of the findings.
7. Is a polygenic structure available to possibly dissect the data further? Are the polygenic risk scores of these individuals calculated and used for interpretation of these subgroups?
8. The reporting of enrichments is overwhelming in the supplementary material as it seems that it was largely data deposition. These enrichments lack directionality, and how these gene programs polarize (enrich/deplete) across populations will assist in interpretation of the plethora of cell populations reported here.

Referee #4

(Remarks to the Author)
I co-reviewed this manuscript with one of the reviewers who provided the listed reports.

Version 1:

Reviewer comments:

Referee #1

(Remarks to the Author)
The authors have satisfactorily addressed all of my previous comments, and I have no further concerns.

Referee #2

(Remarks to the Author)
The study presents a large-scale, integrated single-nucleus multi-omic analysis of Alzheimer’s disease–associated cellular programmes across multiple brain regions and population groups. The dataset remains a valuable resource, and the analytical framework is both modern and appropriate. In its revised form, the manuscript more carefully delineates what can and cannot be inferred from the data, which substantially improves its scientific clarity and reliability.

Key points from the revision with respect to my earlier concerns

1. Interpretation of “shared” signatures across population groups
Replacing the term “conserved” with “shared across population groups” is appropriate and addresses the core interpretational concern raised previously. The addition of interaction models, effect size comparisons, and multi-method validation provides further support that the reported associations are not simply artefacts of non-heterogeneity. While these analyses do not establish biological equivalence across groups (which is inherently difficult in this design), the revised framing now accurately reflects the level of inference supported by the data.

2. Consideration of genetic ancestry and environmental context
The inclusion of analyses based on genetic ancestry (WGS PCs and admixture-defined groups) is an important addition. The observed concordance between self-reported and genetically defined groupings strengthens confidence in the robustness of the findings, while the authors appropriately retain caution in interpretation. The revised text now better reflects the limitations in disentangling ancestry from shared environmental and life-course factors.

3. Robustness of compositional analyses
The incorporation of additional compositional methods (LINDA, sccomp) and the requirement for replication across methods meaningfully strengthen the analytical framework. The inclusion of sensitivity analyses exploring QC thresholds (e.g. UMI thresholds and donor assignment confidence) is particularly valuable and addresses the concern regarding dependence on analytical choices. These additions increase confidence that the main findings are not driven by specific modelling assumptions.

4. Donor assignment and pooling strategy
The additional sensitivity analyses examining the impact of stricter demultiplexing thresholds are appropriate and informative. The observation that key associations remain stable across a reasonable range of parameters is reassuring and sufficiently addresses the concern regarding potential smoothing of inter-individual differences.

5. Observational nature and scope of inference
The inclusion of explicit discussion of the observational nature of the study and its limitations is appropriate and sufficient. The manuscript now more clearly separates association from mechanism.

6. Regional sampling and interpretation
The additional analyses and clarification of region-specific effects improve transparency regarding sampling variability and power. This strengthens the interpretation of regional differences.

The authors have addressed the substantive concerns raised in my initial review. The manuscript is now suitable for publication in its current form.

Referee #3

(Remarks to the Author)
Overall, the authors have addressed most of the major concerns. They strengthened the manuscript by reporting the face-validity of self-reported race/ethnicity compared to genetic ancestry, they made the 10X multiome pooling strategy available, and clarified that AD and MCI subclustering is disease-specific and cannot be mapped in control donors. They acknowledge the work-in-progress nature of endophenotyping and similar operational definitions. The addition of PRS calculations showed no differences between the diseased subgroups. The WGCNA analysis is appreciated, but it is not reported systematically if it is intended to support the preference of factor-analysis. However, an important remaining issue is the insufficient clarity surrounding results of Figure 4. I outline my major outstanding concerns here:

1. Figure 4 requires a clearer interpretation. The negative slope of cognitive decline remains still difficult to follow. The authors report having revised the Figure and scatterplots for GPNM and ATHGAP24. It is not clear what specifically changed compared to the original submission. Signs were flipped, but the axes appear similar while the plotted data differ, additional clarifications are needed to understand whether the changes are interpretative or analytical.

2. The rationale for cell types shown in Figure 4 should be explicit. It is not clear why now Oligo_EPHB2 is shown now instead of Gaba_SST_CHODL, which was included in the original submission. Selection of examples strongly influences the interpretation of the figure, the authors must explain the rationale for these choices.

3. Changes in Figure 4e associations are not described. Oligo_EPHB2, which previously was not significant is now significantly associated. Glut_L23_CUX2_CCDC68 was previously significant and it appears no longer significant in the current analyses. These layout revisions and altered associations were not outlined in the rebuttal for this reviewer or any other reviewer. This needs clarification.

4. The addition of WGCNA should be reported more systematically. The addition of WGCNA is useful as a comparative analysis. However, in its current form, it only supports the preference of the authors for factor analysis and hierarchical clustering for interpretation purposes. The WGCNA should be reported more systematically in the manuscript or supplement.

Referee #4

(Remarks to the Author)
I co-reviewed this manuscript with one of the reviewers who provided the listed reports.

Version 2:

Reviewer comments:

Referee #3

(Remarks to the Author)
In the last second round of review, four points were raised. Authors have addressed these points with transparency.

One remaining point needs clarification:
In Figure 4e “Xenium proportions (DLPFC, n=33)” is stated, however, the legend states that the Xenium results come from n=36. The methods section from the previous version states that n=33 were used while the figure states 36. From the last version to this latest version, we believe there is a typo. Please verify, as removing 3 subjects from one version to the next one should be justified and outlined.

Referee #4

(Remarks to the Author)
I co-reviewed this manuscript with one of the reviewers who provided the listed reports.

Open Access This Peer Review File is licensed under a Creative Commons Attribution 4.0 International License, which permits use, sharing, adaptation, distribution and reproduction in any medium or format, as long as you give appropriate credit to the original author(s) and the source, provide a link to the Creative Commons license, and indicate if changes were made.
In cases where reviewers are anonymous, credit should be given to 'Anonymous Referee' and the source.
The images or other third party material in this Peer Review File are included in the article's Creative Commons license, unless indicated otherwise in a credit line to the material. If material is not included in the article's Creative Commons license and your intended use is not permitted by statutory regulation or exceeds the permitted use, you will need to obtain permission directly from the copyright holder.
To view a copy of this license, visit https://creativecommons.org/licenses/by/4.0/

We thank the reviewers for their detailed and productive comments; addressing these has resulted in a substantially stronger manuscript, particularly with respect to the rigor of the findings, more careful interpretation and evaluation, and the inclusion of additional genetic data to support interpretation.

Reviewer 1

1) There are several numbers in the manuscript that are somewhat difficult to reconcile on a first reading (160 individuals mentioned in the introduction section; "we selected 164 participants" in the Methods section; 167 donors in Figure 1A). Please make sure it is clear for each analysis how many individuals the analysis is based on.

We thank the reviewer for identifying this discrepancy. We have now modified Figure 1 to show the number of donors per region and per population group (which were then used for the meta-analyses of proportions and factors), and have ensured that the manuscript text is consistent.

2) The manuscript states that BH FDR correction was applied "across broad cell types, regions, and clinical and pathological traits". I think it remains unclear what the precise correction universe was. It would be helpful to specify exactly how the FDR batches were defined. For example, whether BH correction was applied (i) within each broad cell class across all cluster times region times phenotype tests, (ii) across all tests in the entire dataset, or (iii) separately for each phenotype or region.

We agree that additional clarification on this point was needed in the paper. We have updated the Methods section to reflect that multiple-hypothesis correction for the proportion and factor analyses (Figs. 1 and 2) was applied within each broad cell type, across all regions and phenotypes, corresponding to (i) in this comment. The number of comparisons per broad cell type was therefore region x phenotype x number of subclusters (or factors).

3) A central strength of the work is the inclusion of African American and Latin donors alongside non-Latin White donors, and the demonstration that key cell-type signatures are conserved across these self-reported groups. The analysis across self-reported race and ethnicity categories is a valuable aspect of this study. If genetic data are available, a brief descriptive characterization of genetic ancestry would, in my view, further strengthen the manuscript. In particular, it would be informative to show how donors from each self-reported category are distributed across the underlying genetic ancestry variation (for example, via PCA or ancestry-proportion estimates), and, where sample size allows, whether the conserved transcriptomic associations are observed across donors spanning different ancestry backgrounds. Such an analysis would add useful context on how the identified shared signatures generalize across both socially defined categories and underlying continuous genetic diversity, without requiring any changes to the core analysis.

This is a key point raised by all three reviewers. To tackle this question, we leveraged publicly available whole-genome sequencing data from the AD Knowledge Portal on Synapse and NIAGADS for 151 of the 167 donors and computed both genetic principal components and admixture components (Figure S7, reproduced below). We observe that PCs 1 and 2 explain a 23% of the variation, and that they align with self-reported race/ethnicity, despite some differences. Similarly, an admixture analysis with k=3 captured the primary ancestry structure, beyond which additional components reflected increasingly fine-scale subdivision (Figure S7b, c). Importantly, self-reported White Non-Latin

individuals had mostly admixture component 1, whereas self-reported African-American Non-Latin individuals had mostly admixture component 3 (with individuals also showing varying levels of admixture component 1). The Latin individuals included those with admixture components 1 and 2, highlighting differences in groupings between self-reported race/ethnicity and genetic ancestry.

Based on this analysis, we re-ran the associations of cell clusters and factors with both the WGS PCs and the admixture components, using an interaction model for the PCs (which have continuous values) and the same categorical approach for admixtures (based on the most dominant component) as for self-reported race/ethnicity in Figures 1 and 2. We observed that most of the major molecular-to-clinical associations we identified shared across self-reported race/ethnicity groups were also shared when donors were grouped by dominant admixture component or when donors were represented by their continuous WGS PCs (Figure S7d-g, labeled in blue text), consistent with the overall similarity between self-reported and genetic ancestry. However, grouping individuals by genetic ancestry did highlight additional signatures that did not cross the FDR correction threshold in our self-reported race/ancestry analysis. These include some vascular cell clusters and glutamatergic neuron factors. Although these differences are minor (see the direct comparison of p-values and effect sizes in Figure S8, also reproduced below), they are still worth reporting as potential differences resulting from self-reported versus genetic ancestry. We have now added a section to the Results and to the Discussion including these new analyses and their implications.

Overall, we are thankful to the reviewers for their suggestion to examine genetic ancestry, which led to corroborating our previous findings on cell type associations shared across population groups, and the discovery of new ones in this study.

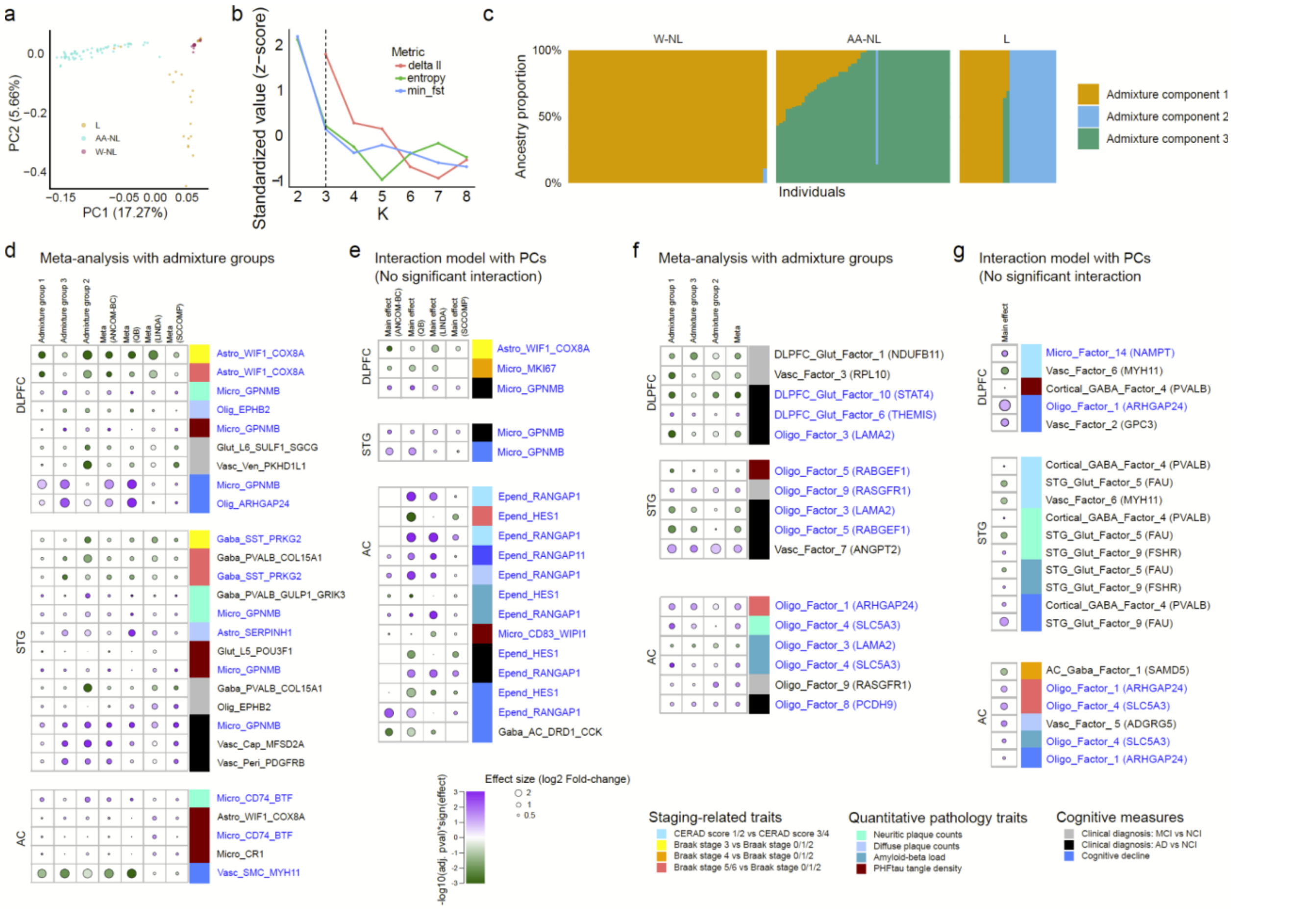

Supplemental Figure S7: Overview of genetic ancestry groupings and associations between clusters/factors and clinical and pathological traits across population groups defined by genetic ancestry. a) Distribution of individual donors along genetic ancestry principal components 1 and 2. Colors indicate self-reported race and ethnicity groups, showing an overall concordance with genetic ancestry variation. b) Assessment of optimal number of admixture components (k) based on multiple information theory metrics. k=3 showed the biggest changes in log-likelihood, normalized ancestry entropy and minimum pairwise Fst. c) Stacked bar plots showing the relative contribution of admixture components (k=3) in each donor, with donors grouped by self-reported race and ancestry. d) Summary of p-values and effect sizes for all cell clusters with statistically significant (meta-analysis FDR-adjusted p-value <0.1, consistent directionality, and meta-analysis I^2<50% in at least two out of four compositional analysis methods) and consistent associations with pathological and cognitive phenotypes across all three population groups defined by dominant admixture component. For visualization, effect sizes and FDR-adjusted p-values for each population group are from the ANCOM-BC method to assess cluster proportion associations. NCI: No cognitive impairment, MCI: Clinical diagnosis of Mild cognitive Impairment, AD: Clinical diagnosis of Alzheimer's dementia. e) Summary of p-values and main effect sizes from models relating cluster proportions to phenotypes including interactions with genetic ancestry PCs 1 and 2. All visualizations are only for region-cluster-phenotype models where the main effect (with phenotype of interest) was significant and neither the interaction with PC1 nor the interaction with PC2 was significant; this combination highlights cluster-phenotype associations that are not differential across genetic ancestry PCs1 and 2. Cell clusters in blue were also

found to have associations shared across groups based on self-reported race and ethnicity (Main Figure 1). f) and g) Same as d) and e), but using the linear model for factor association with a meta-analysis on admixture-defined population groups (f) and interaction model with genetic ancestry PCs (g).

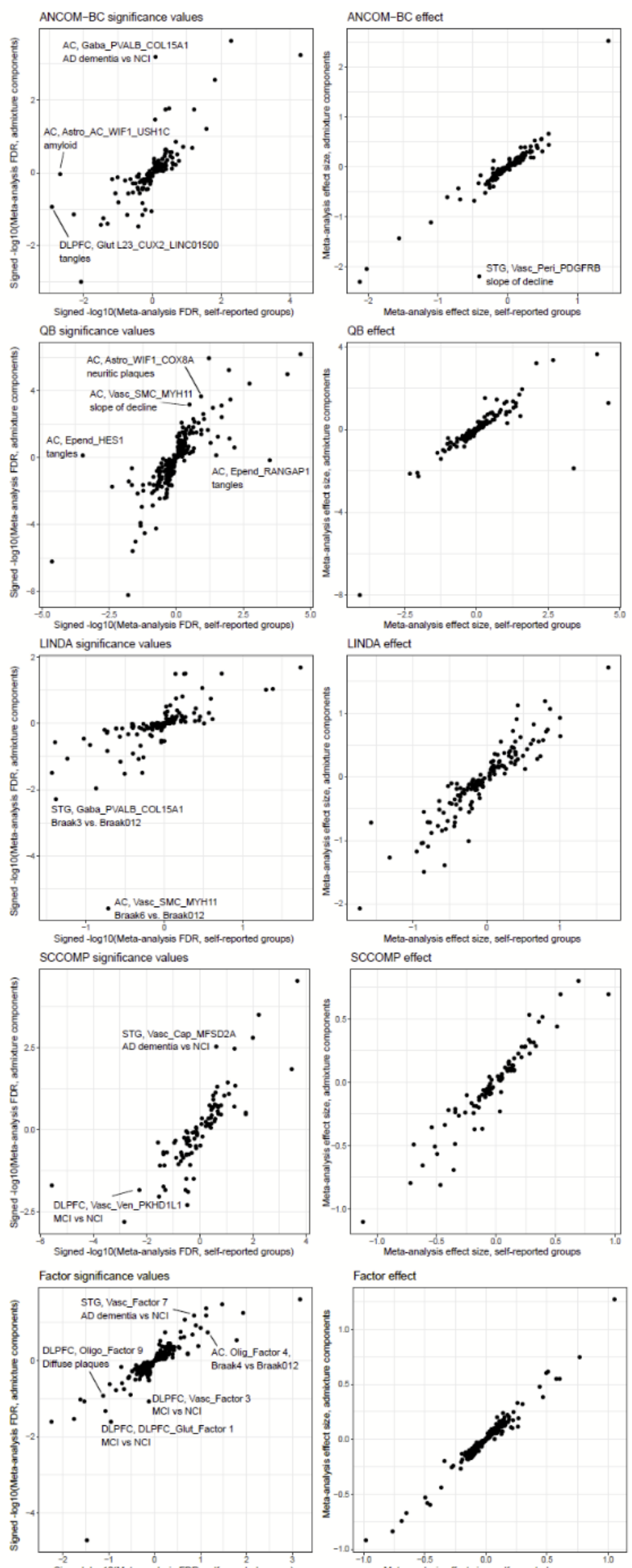

Supplemental Figure S8: Visualization comparing meta-analysis p-values (left column) and effect sizes (right column) for cluster/factor associations with phenotypes using self-reported race and ethnicity groupings (x-axis) versus genetic admixture-defined groupings (y-axis). Each row represents a different method: ANCOM-BC cluster associations (top row), the quasibinomial regression model cluster associations (second row), LINDA cluster associations (third row), sccomp cluster associations (fourth row), and linear model for factor associations (bottom row). Labeled combinations of region, cell type, and phenotype represent associations with substantially different meta-analyses p-values between the x- and y-axes (left column) or substantially different effect sizes between the x- and y-axes (right column).

4) The identification of six donor subgroups based on STG composition and factor values is intriguing. Because subgroup discovery in relatively small cohorts can be sensitive to analytical choices, it would strengthen the interpretation to provide a bit more context on the robustness of these groupings. In particular, could the authors comment on the stability of the subgroup structure? For example, whether similar broad groupings are observed under reasonable alternative clustering settings or distance metrics? Even a short qualitative statement would help readers understand the extent to which these six groups should be viewed as preliminary candidate molecular subtypes, pending replication in larger cohorts.
This is a key point that was missing in our original submission. We have now addressed this by: 1) providing more information about the parameter search initially used to identify as robust a set of clusters as possible;, and 2) running a systematic comparison of alternative clustering methods to evaluate whether other approaches with similar or better robustness yield similar groupings of donors.
For 1), we expanded the Methods section to include how we used a parameter search over the number of clusters to optimize the Silhouette score for each partitioning; this was the basis for the six clusters reported in Figure 5.
For 2) Specifically, we show that with added perturbations through a) added amounts of noise to each feature or b) shuffling features across observations, the Jaccard similarity (1 = identical; 0 = dissimilar) between the original partitioning and the noise-based partitioning decreases across methods, as expected. We found that hierarchical clustering using Ward's method and Spearman correlation (the original approach in the manuscript) ranked near the top among all methods, although PAM (Partitioning around Medoids) clustering was slightly more robust in both perturbation analyses. As a result, we compared the PAM clustering partitions with our original clusters identified via hierarchical clustering with Ward's method and found a large overlap between the two. Therefore, we decided to keep our original clustering solution, with additional explanation about the clusters obtained through the PAM method. These results showcase the robustness of the six molecular subtypes we originally identified. These results are now included as Figure S12, reproduced below.

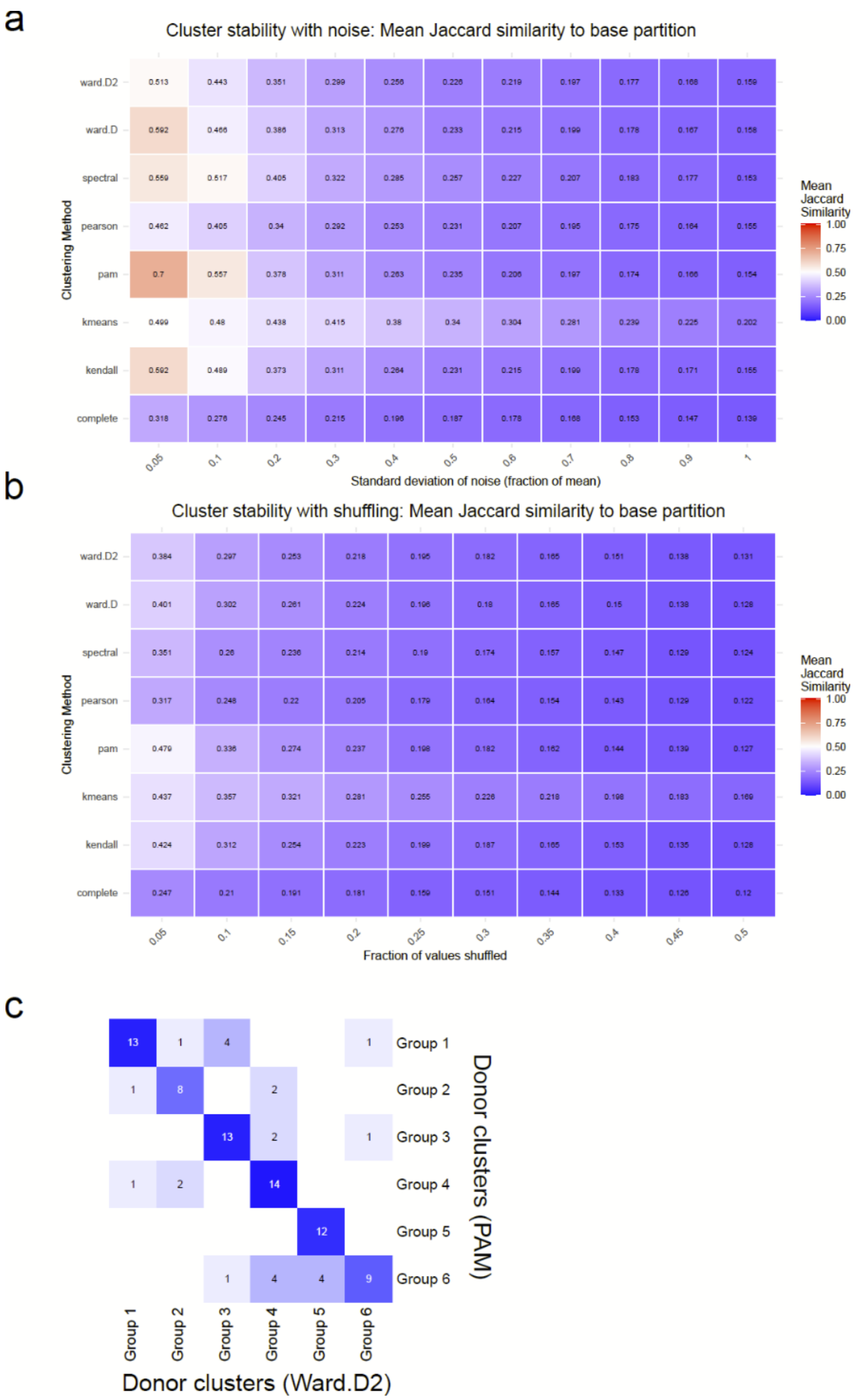

Supplemental Figure S12: Assessment of MCI/AD donor clustering robustness across different methods. a) Heatmap showing the effect of randomly distributed noise on 8 different clustering workflows. Individual cells represent the Jaccard overlap of the partitioning obtained by each clustering workflow (rows) in the reference partition (zero-noise case) versus partitionings obtained at varying amounts of noise (columns, with each noise level having a standard deviation as a given fraction of the mean for each feature). The 17 agglomerated features are combinations of cluster and factor proportions with correlated values across all the donors. As expected, the Jaccard overlap decreases with higher noise levels, but this decrease is less for the PAM workflow, hierarchical clustering with Ward's D method and Spearman correlation ("Ward.D") and hierarchical clustering with Ward's D method and Kendall rank correlation ("Kendall"). b) Same as a), but with feature values randomly shuffled instead of being altered with noise. As in a), the Jaccard overlap decreases as more values are shuffled, with the same three workflows showing the lowest decrease: PAM, hierarchical clustering with Ward's D method and Spearman correlation ("Ward.D") and hierarchical clustering with Ward's D method and Kendall rank correlation ("Kendall"). c) Confusion matrix of donor assignment into clusters using the PAM workflow and the hierarchical clustering with Ward's D method and Spearman correlation, showing overall agreement in the donor clusters between these two workflows, which also exhibit the greatest stability in the face of noise and shuffling as shown in a) and b). The Ward's D method and Spearman correlation workflow clusters correspond to the panels in Main Figure 5.

Despite these new analyses, we agree with the reviewer that the donor subgrouping here **is still preliminary**, given the relatively small number of donors, and that **validation in larger cohorts** will be necessary in future studies to draw stronger conclusions about these subgroups. This caveat is now mentioned explicitly in the Results and Discussion.

5) The observation that the KANSL1 locus shows lower chromatin accessibility in African American donors compared to non-Latin White donors is intriguing. If I did not overlook this, it would be helpful to clarify whether this difference is supported by a formal statistical test, and, if so, how multiple comparisons were handled.

This is an excellent point. We have now included a table (Supplemental Table 10) with the results of running a (nonparametric) Kruskal-Wallis test (across the three population groups) on each locus in each cell type. After Bonferroni correction, we then performed Mann-Whitney tests between pairs of population groups on those locus+cell type combinations. Of the statistically significant results (adjusted p-value <0.05), the KANSL1 locus has the smallest adjusted p-value across multiple cell types, confirming the visual impression conveyed from the mean pileups in the heatmap in Figure 3. We have now included this statistical result in the text and in Supplemental Table 10.

6) The sentence on lines 300–302 appears truncated (".... ARHGAP24+ oligodendrocyte proportions and more severe ....").

We have updated the text to reflect the full sentence.

7) There appears to be a minor typographical error on lines 373–376 ("re not distinguished"), which I believe should read "are not distinguished".

We have updated the text to reflect the complete sentence.

8) For the donor subgrouping, a short justification for choosing STG specifically (as opposed to another region or combined regions) would be useful.

This is a good point, and was not justified in the original manuscript. We have now included text outlining the decision for our selection. Briefly, selection of the STG is motivated by a practical concern - we have the largest number of samples from donors with cognitive impairment - and one biologically relevant reason - the STG exhibits the most variation in pathological burden of the three regions, showing accumulation of tangles earlier than the DLPFC or the AC (which shows very little tangle burden), according to Braak staging. We have updated the manuscript to include these justifications.

Reviewer 2

1. A central claim of the manuscript concerns the consistency ("conservation") of disease-associated signatures across population groups. Although the authors clearly define "conserved" in statistical terms (meta-analysis significance, consistent direction of effect, and limited heterogeneity), which improves transparency, the manuscript remains affected by an important interpretational issue. Specifically, the current analyses do not fully distinguish whether the observed consistency reflects: (i) true biological similarity across groups, (ii) limited statistical power to detect differences, or (iii) modelling and normalisation choices that may bias results toward similarity. Where possible, the manuscript would be strengthened by more direct tests of difference (e.g. sensitivity analyses, effect-size comparisons, or explicit interaction-style reasoning) that demonstrate meaningful similarity rather than simply non-heterogeneity. In parallel, the language used to describe "conservation" should be carefully reviewed and not used broadly to avoid over-generalisation.

This is a key point, and was underexplored in the original manuscript. We have now conducted a number of analyses that suggest the meta-analysis is not merely identifying non-heterogeneity, but also accept that these analyses (described below) are still not sufficient to merit the word "conservation". As a result, **we have replaced all mentions of "conserved" in the text with "shared across population groups"**, which is a better interpretation of the results of the meta-analysis approach.

1) As suggested by the reviewer, we implemented an explicit interaction model approach to compare with the meta-analysis results. Here, instead of stratifying models by population group, we include the population group as an interaction term with the phenotype of interest, and examine which molecular signatures show a significant main effect (overall phenotype association) without a significant interaction effect (differential association across population groups). We then compare the significance of this interaction term with the significance from the meta-analysis model. As expected, all models with significant interaction terms are not deemed significant in the meta-analysis (top-left corner of plots below). However, if the meta-analysis were just identifying non-heterogeneity, we would expect many non-significant interaction models to have significant meta-analysis p-values (large numbers of points in the bottom-right corner of the plots below). However, only a small subset of non-heterogeneous models based on interaction terms have significant meta-analysis p-values, suggesting that the meta-analysis is not merely picking up non-heterogeneity, but is likely identifying associations shared across all three population groups.

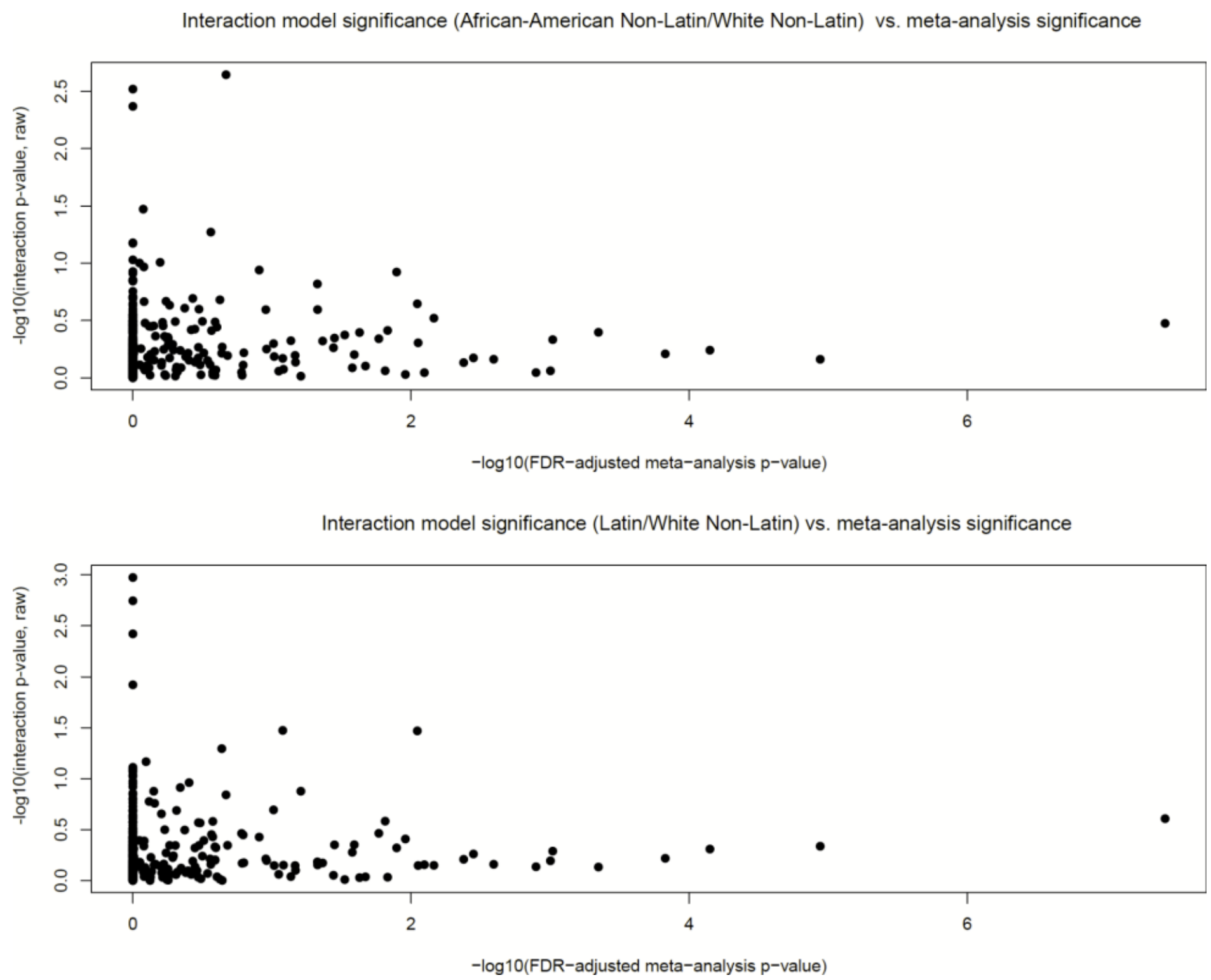

Review Figure 1: *Comparison of adjusted p-values from meta-analysis on cluster proportion associations with clinical and pathological phenotypes across three population groups (x-axis) to interaction model p-values for the same combinations of cluster-phenotype associations (y-axis). The top panel shows interaction p-values for the African-American Non-Latin group vs. White Non-Latin group (reference group), whereas the bottom panel shows interaction p-values for the Latin group vs. White Non-Latin group. These p-values are obtained using the ANCOMBC approach, but are consistent with findings from the three other proportion modeling approaches now described in the text.*

2) For cell cluster proportion associations, we have now incorporated four methods into the manuscript (ANCOM-BC, Quasibinomial regression, LINDA and sccomp), each with different underlying models for assessing proportion differences in relation to clinical traits. By reporting only associations with significant meta-analysis p-values from two or more methods, we ensure that this approach is not unduly biased by the propensity of any given method to be skewed toward non-heterogeneity versus truly shared associations across population groups.
3) An important clarification we have added to the text and the methods is that in order for an association to be shared across groups, the I-squared value from the meta-analysis has to be <50%, and the sign of the effect has to be in the same direction for all three population groups, thus reducing the possibility that the meta-analysis is identifying associations that are substantially different across population groups.
4) As suggested by the reviewer, we have compiled the effect sizes per population group versus the meta-analysis effect size. In the plot below, it is clear that the meta-analysis effect sizes for associations passing the meta-analysis FDR-adjusted p-value are skewed towards conditions where the median and minimum per-group effect sizes are larger. This suggests that the significant meta-analysis findings (which make up a small fraction of the total, as shown in the

middle panel) are not merely selecting for all non-heterogeneous effects, but are identifying conditions where each individual group's effect size is larger than expected.

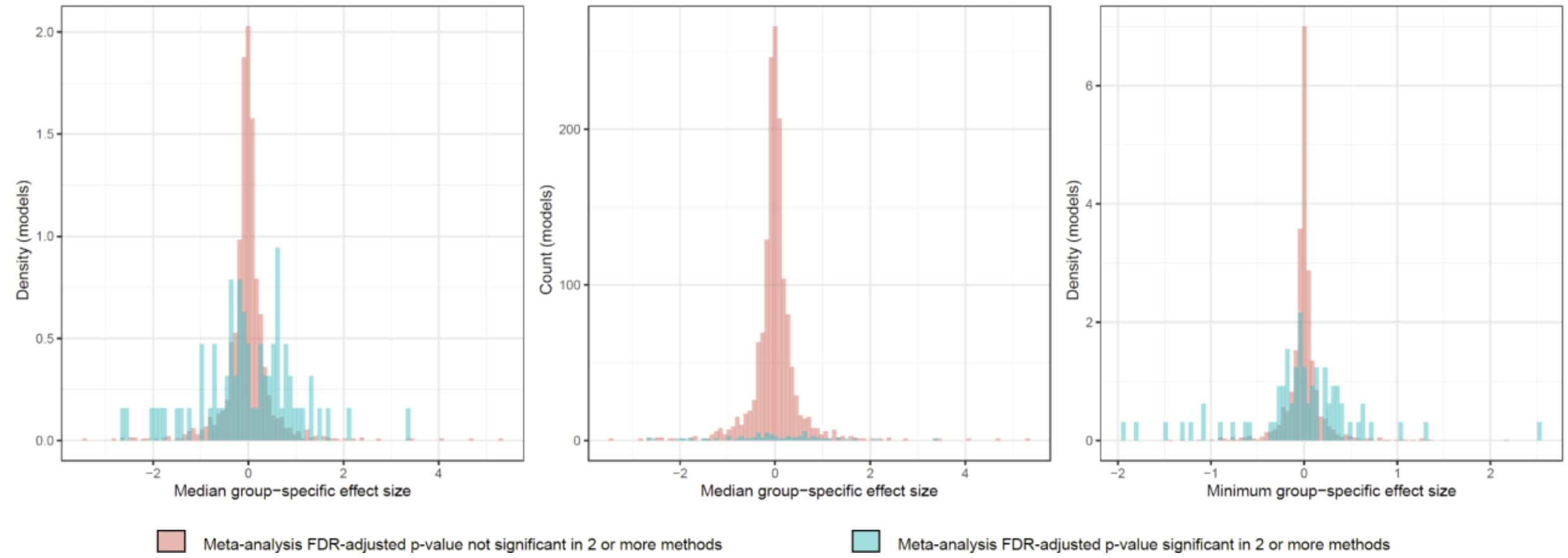

Review Figure 2: Effect size histograms for all models (red) versus models found to be significant in the meta-analysis (blue). Left panel shows the normalized probability density of the median group-specific effect size (median value of effect size among the three population groups) of each model. There is a noticeable shift of the effect size in significant meta-analysis models (blue distribution) towards higher absolute values, suggesting they are not unduly influenced by the maximum effect size within only one population group being analyzed. Middle panel shows the same as the left, but with actual numbers of models instead of normalized probability density; the key point is that only very few models are found to be significant by the meta-analysis. The rightmost panel is the same as the leftmost, but instead showing the minimum effect size from the three population groups. There is still a noticeable shift in the models found to be significant in the meta-analysis, suggesting that this meta-analysis approach is indeed identifying associations that are more likely to have effects in all three population groups, rather than being dominated by large effect sizes in a single population group.

2. The use of self-reported race and ethnicity is transparent and appropriate. However, because the donors largely share a broad geographic and societal context, the observed consistency of cellular signatures could plausibly reflect environmental convergence and shared life experience rather than ancestry-independent biology. As such, the study cannot establish that the reported signatures are conserved across populations in a global or genetic sense, particularly in the absence of geographically distinct cohorts or analyses stratified by genetic ancestry. Although this limitation is acknowledged, the framing should be more consistently maintained across the manuscript to prevent readers from inferring conclusions about genetic ancestry that are not directly supported by the data.

This is an excellent point, and the importance of an analysis including genetic ancestry was raised by all reviewers. We have therefore included an entirely new figure (Fig. S7) that includes all of our cell cluster and factor analyses, rerun with the associations between pathological and cognitive traits and 1) continuous WGS PCs and 2) WGS admixture-defined population groups. Most of our original cell cluster and factor associations are also found in these two analyses. In addition, this genetic ancestry-based analysis identifies some new associations that did not reach significance in the self-reported race/ancestry analyses, particularly among glutamatergic factors and vascular cell clusters.
Although there are some differences in the significant associations between clinical phenotypes and clusters/factors in self-reported race/ethnicity and genetic ancestry analyses, the overall coherence

between the two sets of analyses is high (see the new comparison Figure S8. Given this similarity, it is not possible to determine whether the differences between self-reported and ancestry-based group associations are truly due to environmental or life-experience effects on the population groups or instead to a lack of power. As the reviewer points out, this is a limitation of this type of study, and we have included text in the results and discussion to ensure the findings are appropriately framed.
Both new Figures - S7 and S8 - are reproduced, together with a detailed explanation of the new analysis, in response to Reviewer 1's comments above.

3. A major limitation of the study is that it is fundamentally observational, relying on associations between cellular states and clinical or pathological phenotypes. Although this is expected and acceptable for a study of this scope.
We thank the reviewer for mentioning the nature and scope of our analysis. This limitation is inherent to large-scale human brain tissue and single-cell profiling studies of this scope, both of which are recognized challenges in the field. We have now included a paragraph in the Discussion explicitly mentioning the observational nature of this study as a main caveat.

4. Many of the key conclusions rely on differential abundance or compositional analyses of cell subclusters. Such analyses in single-nucleus datasets are inherently sensitive to nucleus recovery, QC thresholds, batch effects, regional sampling imbalance, and the choice of compositional model. While the analytical framework is sound and clearly described, the manuscript does not sufficiently demonstrate the robustness of key abundance findings to reasonable alternative analytical choices. This would be strengthened by additional robustness or sensitivity analyses (e.g. alternative QC thresholds, stricter filtering, or complementary compositional modelling strategies) showing that the main conclusions are stable. But if this was done, then it needs to be clearly stated in the manuscript.

1) In response to this comment, we have now included additional compositional methods in the analysis: LINDA and SCCOMP. This allows us to highlight associations that are reproduced across 2 or more compositional methods, thereby avoiding biases introduced by any given method.
2) In addition, we have included new sensitivity figures that show the variation in association due to different thresholds for the minimum UMI count (a QC threshold that can drastically impact the results), as well as in the parameters used to uniquely and confidently assign nuclei to an individual donor in each batch. The meta-analysis's significance and effect size are still observed despite modifying the threshold and parameters, demonstrating the robustness of the key associations reported in the manuscript. These supplemental figures (Figs. S3 and S5) are now reproduced below, as a combined analysis addressing review points 4 & 5. We have also included the full results of these parameter explorations as Supplemental Tables 3 and 4).

5. Pooling of samples prior to library preparation with subsequent genetic demultiplexing is a reasonable design choice. However, unavoidable misassignment, undetected same-donor doublets, or exclusion of ambiguous nuclei may reduce between-donor variance and bias results toward apparent similarity across groups. This is particularly relevant given the emphasis on cross-group consistency. The manuscript would be strengthened by more explicit reporting of donor assignment confidence and exclusion rates, as well as sensitivity analyses

demonstrating that key cross-group conclusions persist under stricter singlet or assignment-confidence thresholds.

An excellent point - as described in the response to point 4 above, we have now run the association analysis for varying combinations of stricter donor assignment confidence threshold convolved with another key QC measure (minimum UMI count per nucleus). As intuited by the reviewer, there is variation in the strength of association: for cluster associations, this appears to be more strongly affected by the donor assignment threshold rather than the minimum UMI count threshold, although there is a strong confound in the reduced number of total nuclei that result from the former. For factor associations, the variation is less, with some factor associations actually increasing in effect size with higher UMI thresholds. Overall, however, the associations remain stable except for very high values of the donor assignment threshold. These results are now included as additional supplemental figures in the manuscript (Figs. S3 and S5) and as Supplemental Tables 3 and 4, and we have added text in the results to highlight the effect of these parameters.

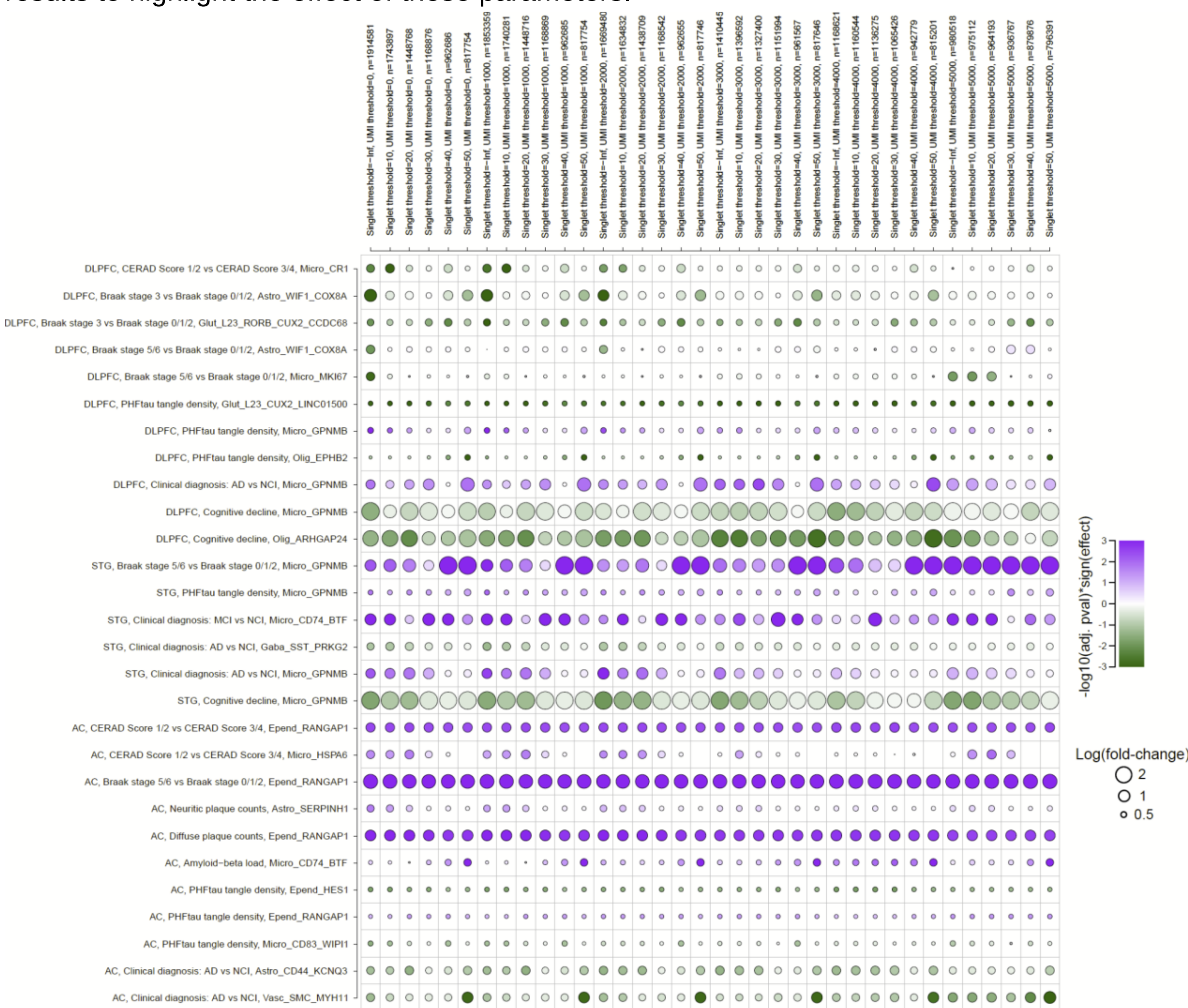

Supplemental Figure S3: Visualization of effect sizes and p-values of ANCOM-BC model cluster proportion associations across a range of parameter values for the UMI threshold and the thresholded difference in likelihood between a singlet and doublet (sngdbldiff) using the freemuxlet package. Row labels indicate the combination of brain region, cell cluster, and phenotype of interest; these include

only the significant combinations outlined in Figure 1. Column labels indicate the singlet threshold (where -Infinity is the default), UMI threshold, and total number of nuclei retained after applying these thresholds. Note that UMI=0 corresponds to the default thresholding of 500 UMIs, so the first column shows values reported in the main figure.

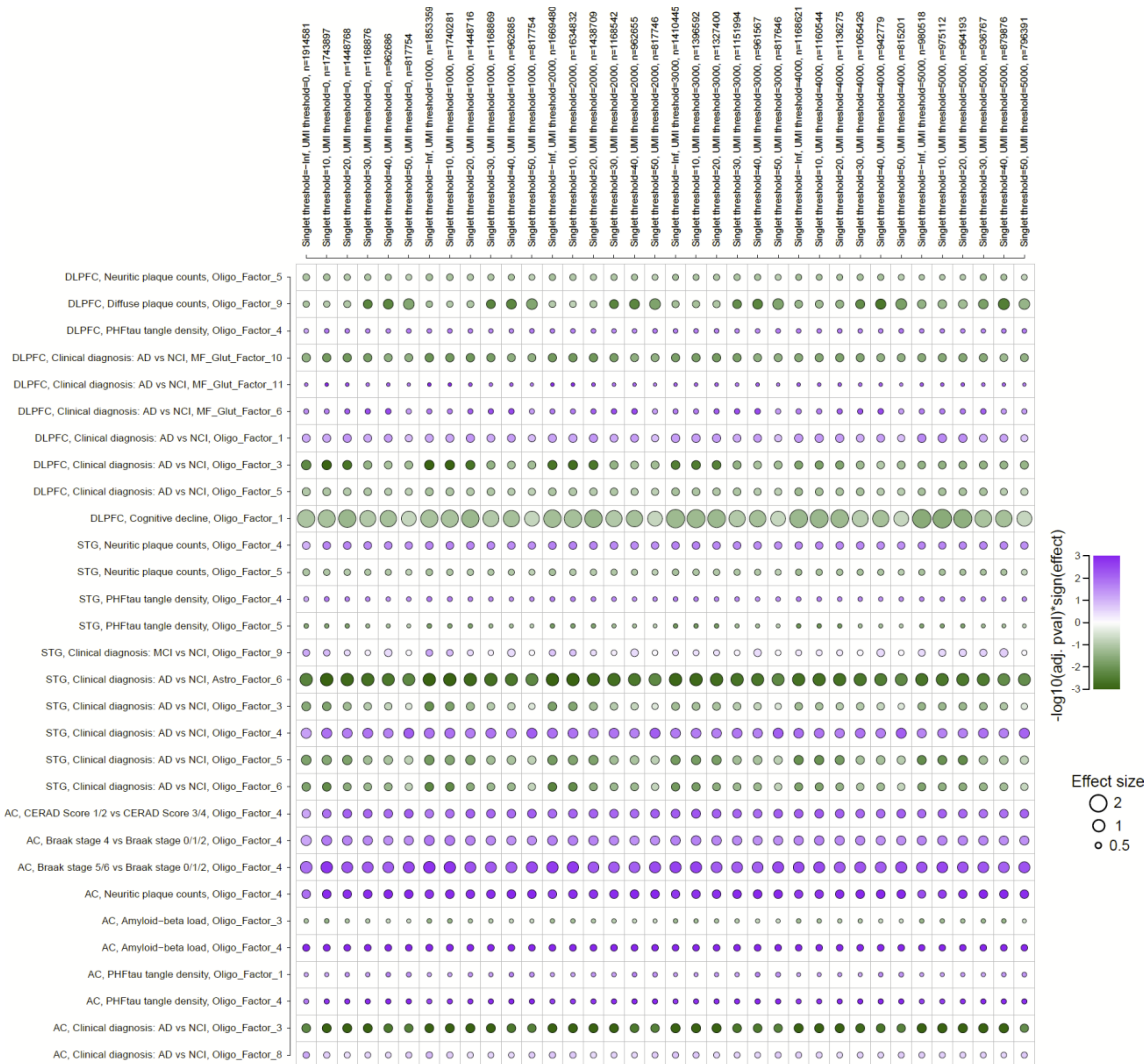

Supplemental Figure S5: Visualization of effect sizes and p-values of linear models evaluating factor associations with clinical and pathological phenotypes, across a range of parameter values for the UMI threshold and the thresholded difference in likelihood between a singlet and doublet (sngdbldiff) using the freemuxlet package. Row labels indicate the combination of brain region, factor, and phenotype of interest; these include only the significant combinations outlined in Figure 1. Column labels indicate the singlet threshold (where -Infinity is the default), UMI threshold, and total number of nuclei retained after applying these thresholds. Note that UMI=0 corresponds to the default thresholding of 500 UMIs, so the first column shows values reported in the main figure.

6. On a more general note, the manuscript will benefit from clarity in handling and interpretation of region-specific effects when not all individuals contribute all brain regions.

We have now included additional mentions in the text of the following:

1) A new figure panel in Figure 1 showing the breakdown of population group samples by donor, highlighting the variability in region sampling in the study and its associated caveats.
2) An additional section on region-specific effects, described in more detail, with particular emphasis on which findings are likely to be due to sampling and power issues, and which findings are likely to be region-specific effects. This includes a new supplemental figure (S6) showing the relative differences in significance and effect sizes across pairs of regions (reproduced below).

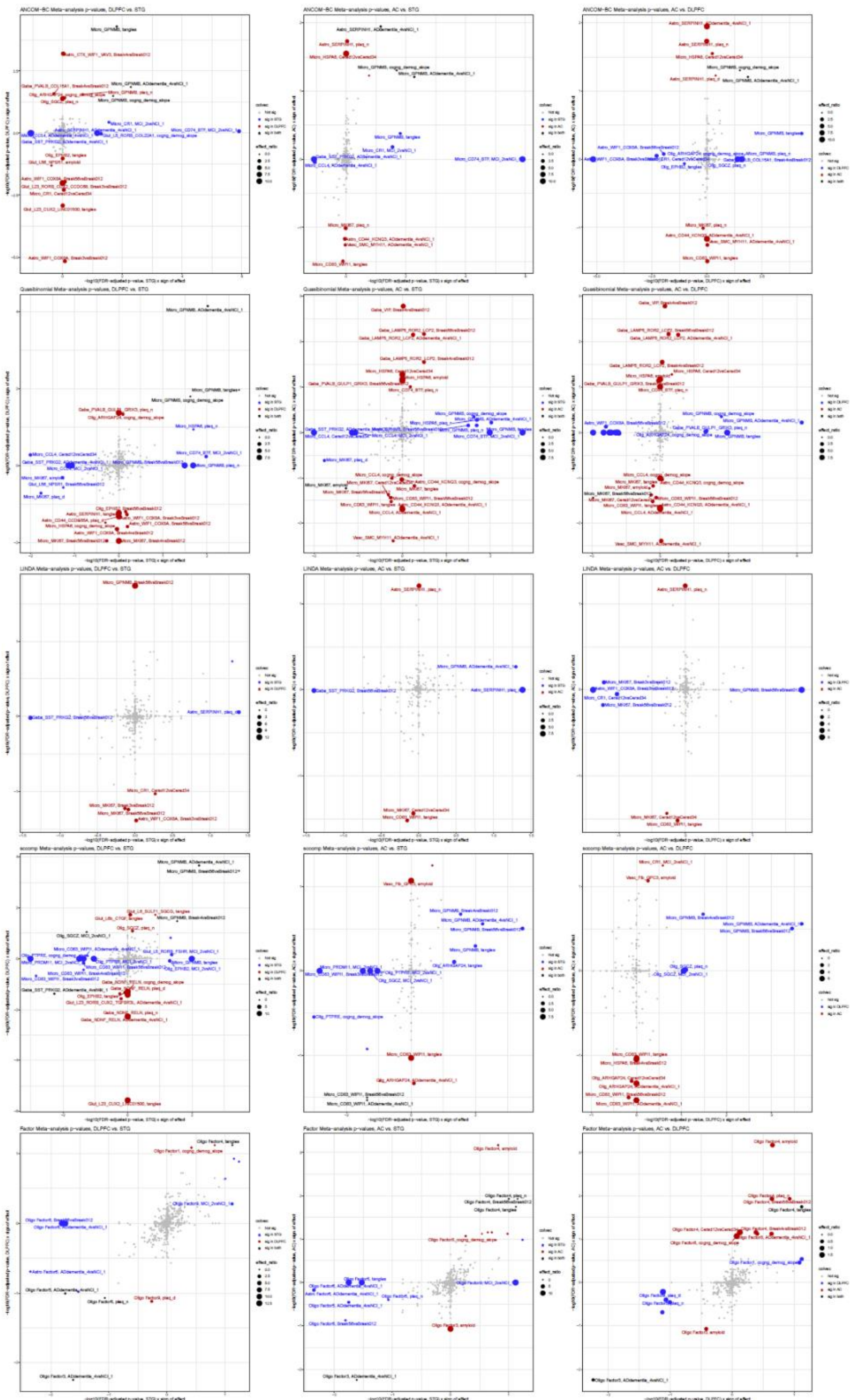

Supplemental Figure S6: Visualization comparing region-specific p-values and effect size ratios for ANCOM-BC cluster associations (top row), the quasibinomial regression model cluster associations (second row), LINDA cluster associations (third row), sccomp cluster associations (fourth row), and linear model for factor associations (bottom row). The first column compares STG (x-axis) and DLPFC (y-axis) p-values. The middle column compares STG (x-axis) and AC (y-axis) p-values. The third column compares DLPFC (x-axis) and AC (y-axis). The size of each dot represents the absolute value of the log2-transformed ratio between effect sizes in the two regions being compared. Colors indicate whether a specific cell type/factor association with a phenotype was significant in both regions (black), only in one region (red or blue), or in neither region (grey).

Reviewer 3

1. Authors employ self-reported race and ethnicity to account for effects of life experience, as they may act as either confounders or disease-driving factors in a polygenic and multifactorial disease such as sporadic AD. Considering multi-ancestry cohort studies such as GWAS or TWAS, where ancestry is genetically inferred with haplotypes, here, race/ethnicity is self-reported. The authors do not explain how self-reported race and ethnicity are different to genetic ancestry. They do explain that self-reported race is used to account for gene-environment interactions during life. How different is the self-reported race from the genetic ancestry for these individuals? Would that impact the whole analysis? This is not outlined in the introduction, methods, or discussion. This benchmarking is crucial, as linkage of the data to ancestry/race is the foundation of the paper. Authors for instance could reconstruct partial genotypes from sn-RNAseq and ATAC-seq data. Alternatively, some (or all) of the donors should be benchmarked by SNP genotyping. More compelling evidence is required to prove face-validity of race/ethnicity self reporting.

This is a key point raised, and brought up by all reviewers. We have now added a substantial new portion of the manuscript, which is the analysis based on genetic ancestry. Fortunately, WGS data has been generated by other groups for a subset of individuals (n=151), and we were able to aggregate this data from the AD Knowledge Portal and NIAGADS to perform the analyses suggested by the reviewer. The results of the analyses, as well as comparisons between self-reported and genetic ancestry cell type association results, are included as Supplemental Figures S7 and S8, with full results included in Supplemental Tables 5-8. Overall, there is strong concordance in the cell type-phenotype associations shared across self-reported groups versus genetic ancestry groups, and this point is highlighted in the updated manuscript. However, there are important caveats to the interpretation (and possible over-interpretation) of results now described in a new paragraph in the Discussion.

To avoid repetition, we refer to the responses to Reviewer 1, point 3 and Reviewer 2, point 2 above, which include the reproduced figures and address the comments from this Reviewer as well.

2. Nuclear suspensions were pooled and then demultiplexed. A supplement to explain the compositions of the suspensions is missing. Authors point to the data availability statement that contains a link to Synapse. However, it cannot be found there. How does this pooling strategy look? Were they balanced by population group or brain region (3 of each)? Nevertheless, this information should be included in the manuscript as a Supplementary Figure or Table and included with more detail in the Methods (Although there are some aspects in the Methods section, it is insufficient).

This is an omission, and we thank the reviewer's careful examination of the deposited data in identifying this discrepancy. The pooling strategy file is kept in a different folder on Synapse (https://www.synapse.org/Synapse:syn53649030) and was not linked to the rest of the dataset. We have now included a direct link in the Data Availability Statement to the pooling strategy file on Synapse to ensure easy access, and mentioned that the pooling strategy can also be reverse-engineered from the metadata file linking donor identity to the pooled batch at the individual nucleus level. We have also added more details to the methods about the pooling strategy: briefly, each pool contained, as far as possible, one sample from each of the three regions and they were balanced across population groups to minimize batch effects.

3. The Xenium validation figure is hard to understand. What is the added value of the gradient of the colored sections? From the visual inspection of the plots with the red dots, Micro_GPNMB does not seem to increase in individuals with more cognitive decline. Negative slope as a continuous variable in such plots makes the analysis convoluted and not intuitive. Authors need to present these data in a more accessible fashion to allow proper interpretation, instead of hard to interpret and integrate spatial plots.

We attempted to show the Xenium data at a high level of granularity to highlight the variability for readers who may not be as familiar with this data modality. However, we agree that showing each individual section in the main figure is overwhelming. We have now modified Figure 4 to show only the summary plots of phenotype versus cell type proportion, and moved the individual tissue section plots to Supplemental Figure S11, in case readers are curious to see the cell-level data. We have also flipped the sign on the slope of the cognitive decline, as suggested by the reviewer - this is now more in line with the notion of “worse phenotype” being towards the right on the x-axis.

If, after these changes, the reviewer still deems the supplemental figure showing every section overwhelming, we can remove the Supplemental Figure.

4. In Figure 5, performing only hierarchical clustering of cell proportions and factors, obtained in the same data seems circular as a standalone analysis. Seems that the same data is “represented” in different fashions, which would not be an orthogonal validation. Orthogonal analyses, for instance with WGCNA or similar strategies, will strengthen these associations and support the claims by the authors.

There are multiple ways to represent snRNA-seq data in reduced dimensions: clusters and factors (gene programs) are one approach, and WGCNA (a different workflow to identify gene modules) would be another approach. We elected to use clusters and factors to investigate the molecular donor subtypes because they complementarily capture discrete states and continuous variation, with some degree of overlap, as noted by the reviewer.

The heterogeneity analysis in Figure 5 is not meant to be a validation of the cell cluster and factor associations in Figures 1, 2, and 4, but rather asks a complementary question: are there non-universal signatures associated with cognitive impairment that are only present in subsets of individuals (distinct from self-reported or genetic population groups), which would not be detected by our standard association analyses aimed at finding global signatures? We have now included additional explanatory text at the beginning of the corresponding Results sections to highlight the distinct question being asked by the analysis in Figure 5.

In addition, as suggested by the reviewer, we also ran two forms of WGCNA to identify gene modules for clustering donors, to see if there were alternative groupings of individuals that showed potential molecular subtypes of dementia. The first approach generated modules within each major cell class (105 total modules), whereas the second approach generated modules using all cell types simultaneously. In both cases (see figure below), we then used these modules to group impaired donors into putative AD subtypes using the same cluster robustness approach described in the methods: a systematic search over the clustering height, distance metrics, and agglomeration strategy, with the objective of optimizing the Silhouette score.

In our optimal grouping of individuals, only one donor subgroup showed enrichment for a specific clinical phenotype (MCI) using the cell type-specific WGCNA modules as features, and one donor subgroup showed enrichment for dementia in the all-cell-types WGCNA module case. Thus, while

these approaches did identify potential molecular subgroups of donors, it was more difficult to interpret what these subgroups mean in the context of cognitive phenotypes and molecular subtypes of MCI and AD. As a result, we have retained our original clustering approach using clusters and factors, with additional text outlining the purpose of the donor clustering problem. In other words, not as validation of our cross-population group associations, but rather to identify molecular heterogeneity among impaired individuals that may cross different racial and ethnic population groups.

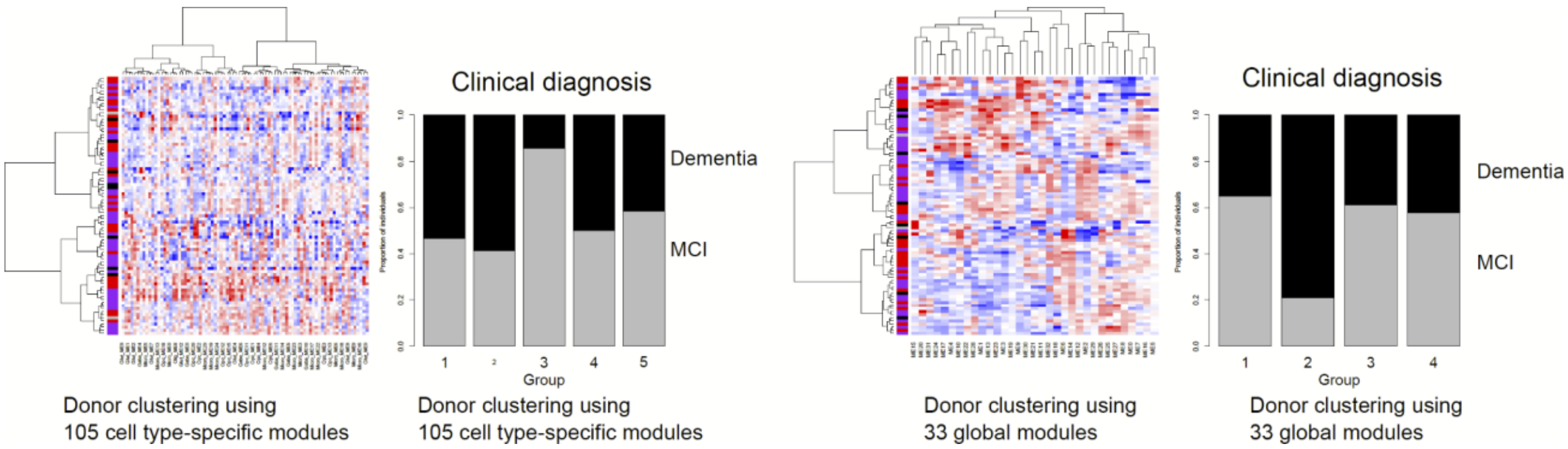

Review Figure 3: Donor clustering using WGCNA modules instead of clusters and factors (from Main Figure 5). The left two panels show donor clustering based on 105 cell type-specific WGCNA modules, where WGCNA was run separately on each broad class to identify modules, and all modules then concatenated together as features to cluster donors. The right two panels shows donor clustering based on 33 WGCNA modules identified using all broad classes together. In the 105 cell type-specific module case, donors group into 5 clusters, with only one cluster showing enrichment for individuals with MCI. In the global module case, donors group into 4 clusters, with only one cluster showing enrichment for individuals with dementia.

5. Comprehensive rationale behind MCI and AD selection only for the definition of subgroups is missing. The motivation towards further dissection of pathological states is clear, but how do controls behave? Or how do they load these factors? It is anticipated that controls also contain a wealth of information on non-pathological data across ancestries.

This is an excellent point that we did not describe in sufficient detail in our original submission. We initially selected only MCI and AD individuals because of the focus on putative molecular subtypes associated with cognitive impairment; both MCI and AD were needed to ascertain whether our molecular subgroups had clinical significance in terms of enrichment of different degrees of severity in the clinical phenotype. As the reviewer points out, the controls themselves may harbor additional information, which could include two scenarios, as outlined below. These results are now included as a new Supplemental Figure (S13) in the manuscript, reproduced below.

1) The groupings of features (cell clusters & factors) may be the same in controls as in the MCI/AD individuals, suggesting that these coordinated changes reflect human heterogeneity rather than disease-specific subgroupings. By comparing the feature cluster overlap between controls and MCI/AD groups (left panel), we identified only a handful of features that showed coordinated variation in the controls and in the MCI/AD groups, suggesting that variation in control populations is along different axes as compared to variation within the cognitively impaired populations.

2) Despite differences in these feature modules, it is possible that donor clustering of the controls would result in groups that have similar overall molecular profiles to those in the MCI and AD groups. We therefore clustered our control donors using a) control donor feature groupings and b) MCI/AD donor feature groupings, to investigate whether the control donor clusters then showed some similarity to the MCI/AD donor clusters. Our results (right panel) show that there may be a weak relationship between a specific control donor cluster (with either set of feature groupings) and two of our AD-enriched donor clusters, suggesting that a subset of control donors may be more likely to follow along a specific trajectory of dementia; however, with the lack of longitudinal data, this remains too speculative to draw further conclusions.

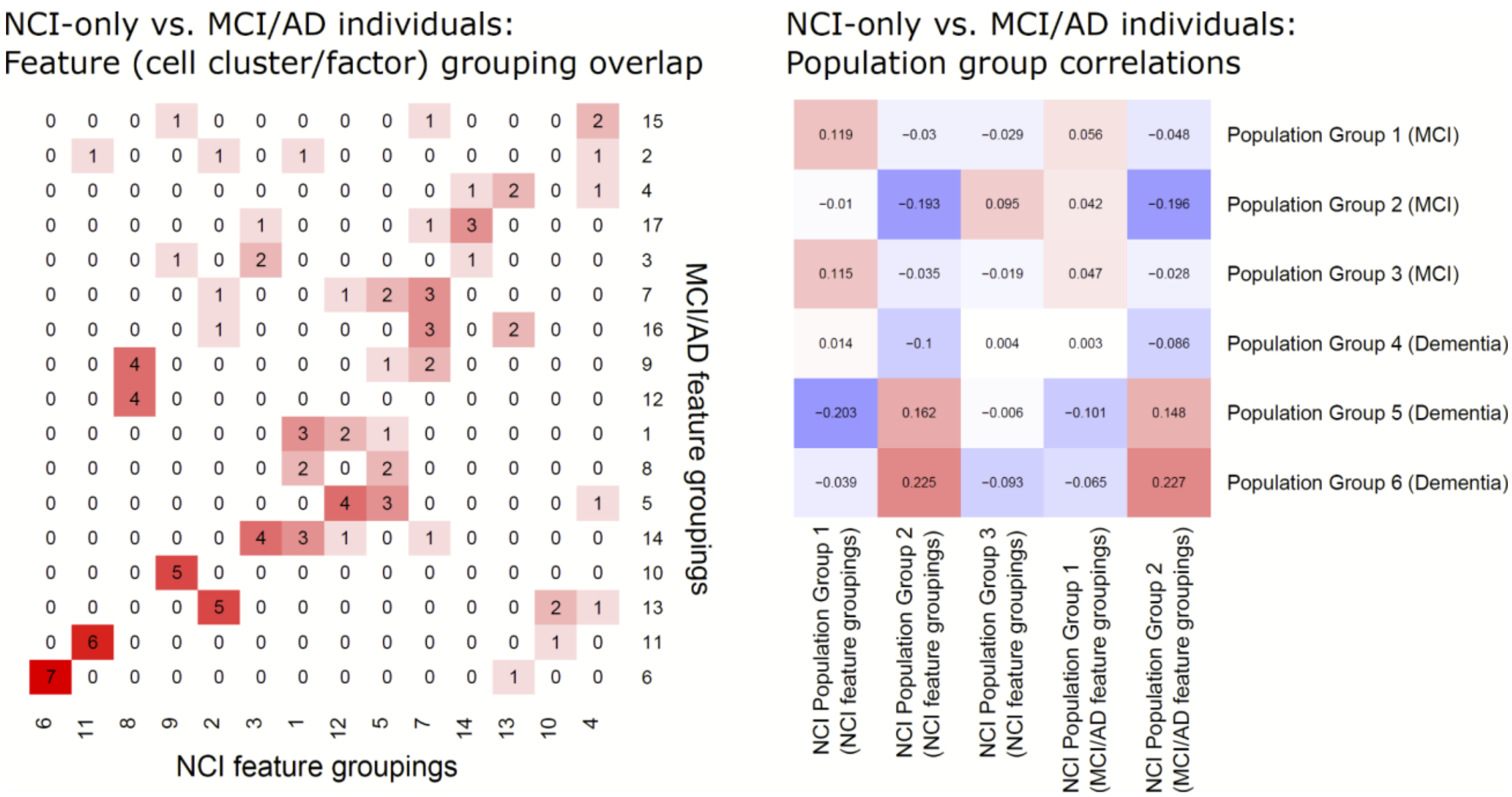

Supplemental Figure S13: Comparison of NCI individual clustering with MCI/AD individual clustering. Left panel: confusion matrix of de novo clustering of features (cluster proportions and factors) in NCI individuals versus in MCI/AD individuals. Whereas some groupings of cluster proportions/factors are similar across NCI individuals and MCI/AD individuals, most cluster proportions/factors show differential correlations in these two sets of individuals, suggesting that coordinated variation in molecular singatures is different in NCI individuals versus individuals with cognitive impairment. Right panel: Overall correlation of mean feature values in MCI/AD population clusters (rows) with NCI population clusters. NCI individuals were clustered in two ways: 1) using de novo groupings of features (cluster proportions/factors) only in NCI individuals, resulting in three NCI groups, and 2) using the groupings of features based on coordinated variation in MCI/AD individuals, resulting in two NCI groups. In both cases, the groups of NCI individuals do not show strong similarities with the MCI/AD groups, based on the correlation of mean feature values. Overall, this suggests that the subgroups of MCI/AD individuals do not reflect heterogeneity found in NCI individuals, but are rather more likely to be distinct molecular subtypes of disease.

6. Presenting the concept of “transcriptomically defined subgroups” is problematic. What is the biological or practical relevance of defining them? It is evident that the interpretation of these groups transcends other categories such as race or pathological features. However, an individual suffering from AD will not be further sub-classified as Group 4, 5 or 6 unless they load heavily on Oligodendrocyte factor 1, Astro_CD44, or Astrocyte Factor 1, respectively. It needs to be clarified. The field of genomics has employed the term “endophenotypes” to propose further dissection of diseases to a higher resolution. Please note that this is an evolving field, and these definitions are always subject to revision. These considerations of further dissection of disease signatures should be addressed at the analysis, interpretation, and discussion of the findings.
We agree with the reviewer that this is a nascent and dynamic field, especially in the context of molecular endophenotypes. Specifically, we state that our grouping of clusters and factors carries biological meaning, as it can cut across expected axes of variation (such as race/ethnicity or APOE genotype) and inform potential clinical interventions targeting these specific clusters or factors, or the endophenotypes they might represent. In other words, we attempt to convey that our donor grouping carries biological meaning and that this biology can have clinical impacts. However, we also recognize the preliminary nature of our grouping and instead suggest to focus on the potential endophenotypes it might represent rather than taking it as a final donor grouping. We have now included text outlining this in both the Results and in the Discussion, with a particular focus on the caveats associated with these findings.

7. Is a polygenic structure available to possibly dissect the data further? Are the polygenic risk scores of these individuals calculated and used for interpretation of these subgroups?
This is an excellent point. From the genetic data that has now been included and analyzed in the updated manuscript, we derived polygenic risk scores and have now plotted them across the 6 population groups. There is no clear distinction in PRS across the groups, suggesting that the molecular distinctions are not driven by the overall genetic risk. Although it is possible that specific variants may segregate across groups, this study is not well-powered in terms of donor numbers per group, consistent with the idea that this grouping is preliminary and needs to be validated in larger studies.

8. The reporting of enrichments is overwhelming in the supplementary material as it seems that it was largely data deposition. These enrichments lack directionality, and how these gene programs polarize (enrich/deplete) across populations will assist in interpretation of the plethora of cell populations reported here.
We agree - there were some places where the data visualization was overwhelming and not carefully organized. This includes the supplemental figure showing all enrichment plots (and also the Xenium results in Figure 4, as described above). To make this analysis easier to parse, we have now condensed all pathway enrichment analyses into a Supplemental Table (Table 2) that can be filtered by cluster, factor, or broad cell class in order to highlight specific enrichments. In addition, the Methods section explicitly mentions that this table only includes signatures that are positively regulated (higher gene expression in clusters, top 100 gene weights in factors), and individual pathway terms, where relevant, include directionality as well. We elected not to include signatures that are depleted in clusters or factors because of potential interference due to substantial repetition of the same signatures across

clusters; upregulation of a set of genes in one cluster also corresponds with downregulation of that signature in all other clusters, leading to a proliferation of terms for those latter clusters. If the reviewer feels strongly that depleted signatures should also be included, we would be willing to add a second, much larger, supplementary table with pathways based on downregulated genes.

We thank the reviewers for their attention to detail and comments regarding the analytical and interpretational aspects of the manuscript. Below, we address the four points raised in the second round of review and outline additional changes to the manuscript. We also note additional changes in the text (visible in the tracked manuscript) to bring text, caption, and title lengths to within journal guidelines. Four of the Extended Data Figures have also been condensed and merged to bring the total (with the inclusion of a new WGCNA figure, see below) to ten figures.

1) Figure 4 requires a clearer interpretation. The negative slope of cognitive decline remains still difficult to follow. The authors report having revised the Figure and scatterplots for GPNM and ATHGAP24. It is not clear what specifically changed compared to the original submission. Signs were flipped, but the axes appear similar while the plotted data differ, additional clarifications are needed to understand whether the changes are interpretative or analytical.

We agree that interpreting the slope of cognitive decline can be unintuitive. To improve clarity, we have changed the x-axis titles for the Micro_GPNMB and Olig_ARHGAP24 scatterplots in Figure 4d from “negative slope of cognitive decline” to “cognitive decline” to match the descriptor in Figures 1 and 2, and in Figure 4e. To ensure there is no confusion about this measure, we have expanded its interpretation in the figure captions, pointed the reader to the Methods section where the measure and references are fully described, and added notes in the main text, outlining that, for reporting associations, we use the negative of the slope of cognitive ability over time.
In case the confusion is on how the measure is itself defined, we include below the plot of individual trajectories (left) and the slope (right). In the original measure reported in Wilson et al. 2019 and Wilson et al. 2020 (as referenced in the Methods), a more negative value of the slope (sharper downward trajectory) is indicative of worse decline. However, for pathological measures (amyloid and tau quantification), a more positive value is associated with worse outcome; for ease of interpretation, therefore, we decided to present maintain a more positive value to be associated with worse cognitive decline, leading to the sign switch on the x-axis in Figure 4.

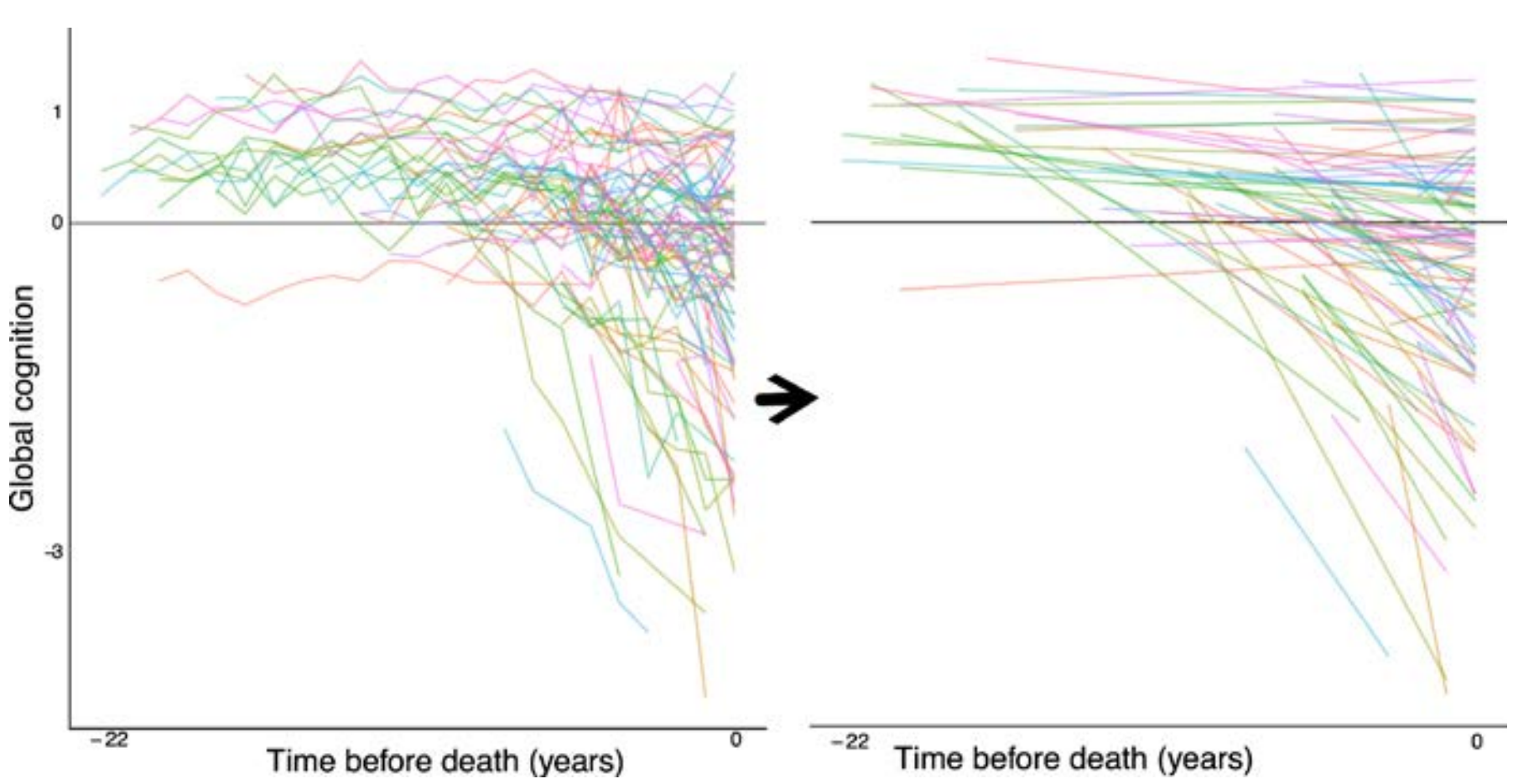

Regarding the differences in the plotted data in Figure 4d, the reviewers are correct that the x-axis values (cognitive decline phenotype) are the same as in the previous version of the manuscript, but were flipped in sign, as explained above. As the reviewers point out, the y-values are also indeed different from the original version in the manuscript, and this was due to our discovery of an error in our original submission: we had run RCTD with the default minimum UMI threshold (min_UMI=100) instead of the reported threshold (min_UMI=20) in the Methods. In the revised version of the manuscript sent to the reviewers, we therefore reran RCTD with the reported threshold (min_UMI=20), regenerated the plots and reran the association analysis, resulting in slight changes in the proportions; although the proportions differ with this re-running, the overall associations remain qualitatively the same. We note that the extended data figure comparing snRNA-seq and Xenium proportions was also altered in the previous revision because of this re-running. For this round of revision, we double-checked that the RCTD estimates were run with the correct UMI threshold (min_UMI=20), and thus the plots and associations remain concordant with the methods.

2) The rationale for cell types shown in Figure 4 should be explicit. It is not clear why now Oligo_EPHB2 is shown now instead of Gaba_SST_CHODL, which was included in the original submission. Selection of examples strongly influences the interpretation of the figure, the authors must explain the rationale for these choices.

We thank the reviewers for bringing up this concern. For Figure 4d, the full granular validation plots shown are for a subset of continuous phenotypes that passed our stringent requirements in Figure 1. Based on Reviewer 2's previous concerns about the robustness of our findings, we re-evaluated all cell type associations using two additional methods (LINDA and sccomp) and included a stricter heterogeneity requirement (based on I2) in the meta-analysis. The inclusion of these thresholds removed several associations reported in the original version of the manuscript, including that between Gaba_SST_CHODL and cognitive decline in DLPFC. As a result, there was no reason to validate this specific association in the Xenium data any more.

As shown in Figure 4e, of the snRNA-seq associations that were significant in the revised manuscript (Figure 1), only three clusters associated with a continuous trait were replicated in the Xenium data: Micro_GPNMB (with cognitive slope and PHFtau tangle density), Olig_ARHGAP24 (with cognitive slope), and Olig_EPHB2 (with PHFtau tangle density). We therefore opted to highlight each of these clusters in the more granular plot in Fig. 4d. We of course continue to include the full results for all clusters (replicated and non-replicated) in Figure 4e and in Supplemental Table 13, as in all previous versions of the manuscript.

To reinforce the notion that only the findings in Figure 1 are being validated in Figure 4e, and that only a subset of these are shown in Figure 4d, we have added text explicitly to the main text and the figure caption linking Figures 4 and 1.

3) Changes in Figure 4e associations are not described. Oligo_EPHB2, which previously was not significant is now significantly associated. Glut_L23_CUX2_CCDC68 was previously significant and it appears no longer significant in the current analyses. These

layout revisions and altered associations were not outlined in the rebuttal for this reviewer or any other reviewer. This needs clarification.

We apologize for the confusion around this issue. This is related to point 2 above - with the additional criteria for snRNA-seq cell cluster associations to ensure robustness, several cluster associations dropped out in Figure 1, whereas Olig_EPHB2 association with tau reached significance because of consistency found with the addition of two compositional methods (Figure 1). In our original manuscript, we did not test the Xenium data for Oligo_EPHB2's association with tau, since it was not included in our original Figure 1 results. In our original manuscript, Oligo_EPHB2 was tested in the Xenium for association with other clinical variables, but these associations no longer passed the snRNA-seq robustness requirements in our revision (Figure 1), so were not tested in the Xenium data in the new version of the manuscript.

Glut_L23_CUX2_CCDC68 remains significant in the current snRNA-seq analysis (Figure 1), and is also significant in the Xenium analysis (Figure 4E). It was actually not significant in the Xenium replication in the original manuscript, and we attribute the crossing into significance to the correct implementation of the min_UMI parameter in RCTD, described above.

As described in the response to point 2 above, we have now explicitly mentioned in the text and the caption that all replication/validation results from Figure 4 are based on the robust findings (i.e., those that pass the requirements for the minimum number of methods and meet the I2 heterogeneity criteria) in Figure 1.

4) The addition of WGCNA should be reported more systematically. The addition of WGCNA is useful as a comparative analysis. However, in its current form, it only supports the preference of the authors for factor analysis and hierarchical clustering for interpretation purposes. The WGCNA should be reported more systematically in the manuscript or supplement.

We appreciate the reviewers' assessment of the WGCNA. In the previous round of review, WGCNA was mentioned in the context of Figure 5 (disease heterogeneity and subgroups), so the implementation and interpretation of the WGCNA results in our revision were focused specifically on the heterogeneity aspect and hierarchical clustering presented in Figure 5. From this comment, it appears that the intention was to include the WGCNA modules in the associations with clinical phenotypes directly, as was done for the factors in Figure 2. We have therefore included additional discussion of the WGCNA results in the main text, together with a new Extended Data Figure 5, a new Supplementary Table with gene module memberships, and a new section in the Methods. Briefly, the significant modules from WGCNA include two oligodendrocyte modules with opposite directions of association, matching those of specific oligodendrocyte factors, as well as astrocyte modules in the AC corresponding to the SERPINH1+ cluster and the CD44+ clusters. We thank the reviewers for clarifying the purpose of the WGCNA comment here, and how it could be used to strengthen the results of the paper.

We thank the reviewers for their assessment of our additional revisions to the manuscript. Regarding the one remaining point below, we have confirmed that the manuscript contained a typo and have fixed it appropriately.

1) **In Figure 4e “Xenium proportions (DLPFC, n=33)” is stated, however, the legend states that the Xenium results come from n=36. The methods section from the previous version states that n=33 were used while the figure states 36. From the last version to this latest version, we believe there is a typo. Please verify, as removing 3 subjects from one version to the next one should be justified and outlined.**

We thank the reviewer for catching this discrepancy. We had not noticed this typo (n=36), and have fixed it in the figure legend to be n=33, which is consistent with the methods and the data.
