# Supplementary material for: Cell-type signatures of Alzheimer’s disease shared across population groups
Source: Nature. 2026 Jul 15;657(8130):191–201. doi: 10.1038/s41586-026-10793-0 (PMC13538069; doi:10.1038/s41586-026-10793-0)
Supplement: Supplementary file 4 [file 41586_2026_10793_MOESM1_ESM.zip › 2025-11-30680C-s1/SuppTableLegends.docx]

**Supplemental Tables**

**Supplemental Table 1**

Effect sizes and p-values of ANCOM-BC, LINDA, sccomp, and quasibinomial models to evaluate cluster proportion differences associated with clinical and pathological phenotypes across self-reported racial and ethnic groups. Columns represent individual self-reported population groups as well as the meta-analysis across all three groups (see Methods).

**Supplemental Table 2**

GO terms enriched in gene sets that are more highly expressed in each cell cluster (compared to all other cell clusters within the broad class) and most highly weighted in each factor.

**Supplemental Table 3**

Effect sizes and p-values of ANCOM-BC model cluster proportion associations across a range of parameter values for the UMI threshold and the thresholded difference in likelihood between a singlet and doublet (sngdbldiff) using the freemuxlet package. Columns are the same as in Supplemental Table 1, specifically only for ANCOM-BC.

**Supplemental Table 4**

Effect sizes and p-values of linear models evaluating factor associations with clinical and pathological phenotypes, across a range of parameter values for the UMI threshold and the thresholded difference in likelihood between a singlet and doublet (sngdbldiff) using the freemuxlet package.

**Supplemental Table 5**

Module gene membership for cell type-specific modules from WGCNA. Each column indicates the module (prefixed by ME) for each cell class group on which WGCNA was run. “None” indicates that a specific gene did not meet the filtering criteria for expression (CPM>10 in at least 5 samples) for that specific cell class.

**Supplemental Table 6**

Effect sizes and p-values of ANCOM-BC, LINDA, sccomp, and quasibinomial models to evaluate cluster proportion differences associated with clinical and pathological phenotypes across genetic ancestry groups. Columns represent population groups defined by dominant genetic admixture component (as opposed to self-reported race and ethnicity) as well as the meta-analysis across all three groups (see Methods).

**Supplemental Table 7**

Effect sizes and p-values of ANCOM-BC, LINDA, sccomp, and quasibinomial models to evaluate proportion differences associated with clinical and pathological phenotypes using interaction terms with genetic ancestry principal components 1 and 2 (interacting with the phenotype of interest) instead of self-reported race and ethnicity or admixture-defined population groupings (see Methods). These models incorporate the continuous nature of the PC values using all donors with WGS data, and therefore do not require a meta-analysis to identify cross-population effects.

**Supplemental Table 8**

Effect sizes and p-values of linear models evaluating factor associations with clinical and pathological phenotypes. Columns represent population groups defined by dominant genetic admixture component (as opposed to self-reported race and ethnicity) as well as the meta-analysis across all three groups (see Methods).

**Supplemental Table 9**

Effect sizes and p-values of linear models evaluating factor associations with clinical and pathological phenotypes using interaction terms with genetic ancestry principal components 1 and 2 (interacting with the phenotype of interest) instead of self-reported race and ethnicity or admixture-defined population groupings (see Methods). These models incorporate the continuous nature of the PC values using all donors with WGS data, and therefore do not require a meta-analysis to identify cross-population effects.

**Supplemental Table 10**

List of gene loci associated with AD, labeled by a representative gene within each locus.

**Supplemental Table 11**

Table of putative transcription factors whose binding sites show enrichment in the open chromatin regions for different clusters and factors.

**Supplemental Table 12**

Results of Kruskal-Wallis (KW) nonparametric test for cell differential occupancy across population groups at each locus in Supplemental Table 9, based on pseudobulked ATAC-seq data per donor per cell type. Post-hoc pairwise test p-values represent Mann-Whitney non-parametric tests between population groups.

**Supplemental Table 13**

Gene list (259 genes) used for Xenium panel for in situ hybridization validation in DLPFC tissue.

**Supplemental Table 14**

Effect sizes and p-values of ANCOM-BC models to evaluate DLPFC cluster proportion (from Xenium ISH data) association with clinical and pathological phenotypes. Cluster labels were transferred to Xenium ISH data using the snRNA-seq data as a reference. Only specific cluster and trait combinations that were found to be significant in the snRNA-seq proportion analysis were tested in the Xenium ISH-derived proportion data.

**Supplemental Table 15**

Effect sizes and p-values of ANCOM-BC models to evaluate DLPFC cluster proportion associations with clinical and pathological phenotypes in external data sets. Cluster labels were transferred from the “source dataset” to the “mapped dataset” in order assess reproducibility of findings from the current study in previous studies and from previous studies in the population groups profiled in this study.
